# Gene Expression and Functional Annotation of the Human Ciliary Body Epithelia

**DOI:** 10.1371/journal.pone.0044973

**Published:** 2012-09-18

**Authors:** Sarah F. Janssen, Theo G. M. F. Gorgels, Koen Bossers, Jacoline B. ten Brink, Anke H. W. Essing, Martijn Nagtegaal, Peter J. van der Spek, Nomdo M. Jansonius, Arthur A. B. Bergen

**Affiliations:** 1 Department of Clinical and Molecular Ophthalmogenetics, the Netherlands Institute for Neuroscience, Royal Netherlands Academy of Arts and Sciences, Amsterdam, The Netherlands; 2 Laboratory for Neuroregeneration, the Netherlands Institute for Neuroscience, Royal Netherlands Academy of Arts and Sciences, Amsterdam, The Netherlands; 3 Department of Bioinformatics, Erasmus University Medical Center, Rotterdam, The Netherlands; 4 Department of Ophthalmology, University Medical Center Groningen, University of Groningen, Groningen, The Netherlands; 5 Department of Ophthalmology, Academic Medical Centre, Amsterdam, The Netherlands; 6 Department of Clinical Genetics, Academic Medical Centre, Amsterdam, The Netherlands; University of Rochester, United States of America

## Abstract

**Purpose:**

The ciliary body (CB) of the human eye consists of the non-pigmented (NPE) and pigmented (PE) neuro-epithelia. We investigated the gene expression of NPE and PE, to shed light on the molecular mechanisms underlying the most important functions of the CB. We also developed molecular signatures for the NPE and PE and studied possible new clues for glaucoma.

**Methods:**

We isolated NPE and PE cells from seven healthy human donor eyes using laser dissection microscopy. Next, we performed RNA isolation, amplification, labeling and hybridization against 44×k Agilent microarrays. For microarray conformations, we used a literature study, RT-PCRs, and immunohistochemical stainings. We analyzed the gene expression data with R and with the knowledge database Ingenuity.

**Results:**

The gene expression profiles and functional annotations of the NPE and PE were highly similar. We found that the most important functionalities of the NPE and PE were related to developmental processes, neural nature of the tissue, endocrine and metabolic signaling, and immunological functions. In total 1576 genes differed statistically significantly between NPE and PE. From these genes, at least 3 were cell-specific for the NPE and 143 for the PE. Finally, we observed high expression in the (N)PE of 35 genes previously implicated in molecular mechanisms related to glaucoma.

**Conclusion:**

Our gene expression analysis suggested that the NPE and PE of the CB were quite similar. Nonetheless, cell-type specific differences were found. The molecular machineries of the human NPE and PE are involved in a range of neuro-endocrinological, developmental and immunological functions, and perhaps glaucoma.

## Introduction

**Figure 1:**
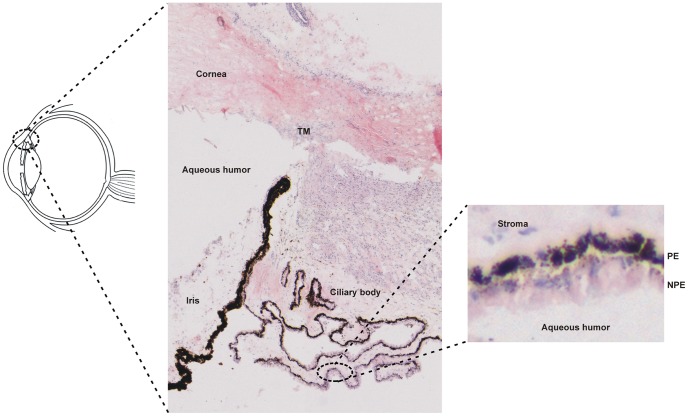
Histology of the ciliary body in the eye. Overview of the anterior part of the eye with Hematoxylin-Eosin staining, showing the cornea, trabecular meshwork (TM), iris and the ciliary body. The ciliary body consists of two epithelial cell layers, the non-pigmented and the pigmented epithelium (NPE and PE respectively). The NPE faces the aqueous humor while the PE is connected to the stroma.

The human ciliary body (CB) is a multifunctional ocular tissue, located between the ora serrata and the iris. The CB is composed of the ciliary muscle and two, partly folded, neuro-epithelial layers: the non-pigmented and pigmented epithelial layers (NPE and PE, respectively). Posterior, the NPE forms, via the ora serrata, a continuum with the neuronal retina, like the PE does with the retinal pigment epithelium (RPE). On the anterior side, the CB continues into the iris epithelium (Figure 1). The CB acts, through tight junctions between NPE cells, as a blood-aqueous barrier; it prevents the intercellular diffusion of large biomolecules from the blood into the aqueous humor [1]. One of the most important functions of the CB epithelia is the production of aqueous humor (AH). The aqueous humor is necessary to build up the intraocular pressure (IOP), which maintains the eye shape, and it nourishes avascular tissues, like the lens and the cornea. The ciliary muscle is involved in lens accommodation. The CB epithelia have also been implicated in a number of other functionalities such as neuro-developmental processes, neuro-endocrine properties, the ocular immune privilege, and the turnover of the vitreous.

The interesting, yet still controversial neuro-developmental function of the CB concerns the recent discovery of retinal progenitor cells in the pars plana and the ciliary marginal zone of the CB. Several authors report that PE cells of human, primate, porcine, rodent (mice and rats) and chicken express a number of retinal stem cell or progenitor cell markers, such as NES, MITF, PAX6, SIX3, Rx, FGF2 and CHX10 [2]–[8]. These findings are corroborated by proliferation and differentiation of isolated retinal stem cells (of human, porcine and rodent tissue) into neural spheres and possible photoreceptor-like cells [5], [7], [9]–[11]. Subsequent reports also describe this proliferation of PE derived cells (of human and rodent tissue) including expression of some retinal progenitor markers, but they fail to observe a differentiation into neurons or photoreceptors [12], [13]. Further investigations are warranted to fully resolve this issue.

Several studies report on the expression and synthesis of multiple (neuro-)endocrine proteins in the CB. These include different neuropeptides, such as neurotensin, natriuretic peptides, and somatostatin, steroid-converting enzymes, transferrin, transthyretin, angiotensin, and growth factors (reviewed by Coca-Prados and Escribano [14]). The synthesized endocrine molecules are most likely important in the pressure regulation and composition of the aqueous humor.

The ocular microenvironment behind the NPE blood-aqueous barrier is immunosuppressive and anti-inflammatory. This minimizes ocular tissue damage and preserves clarity of vision [15]. This immune privilege is maintained by restricting inflow of immunological molecules and cells from the innate an adaptive immune system into the eye. In addition, the aqueous humor is rich in soluble immunomodulatory factors produced by the PE cells such as CD86, TGF-beta and TSP1 [16].

Finally, a number of additional functions have been attributed to the CB, including the macromolecule production and turnover of constituents of the vitreous [17], [18] and accommodation of the lens by the ciliary muscle [19].

The CB is also involved in several pathologies. The most important are glaucoma, anterior uveïtis/iridocyclitis, (pseudo)exfoliation syndrome and uveal melanoma. Since the CB is involved in IOP regulation, it might play a role in primary open angle glaucoma (POAG). POAG is a neurodegenerative disease characterized by abnormal cupping of the optic nerve head and loss of retinal ganglion cells. The pathogenesis of POAG is largely unknown, but a major risk factor is an increased IOP. Moreover, previously several genes are associated with POAG (MYOC, OPTN and WDR36), but only a minor portion of the cases can be explained by genetic variations in these genes [20]. In addition, several biomolecules are previously identified, which showed a pathophysiological relationship with POAG. For example, specific mRNA or protein expression changes are observed in tissue(s) or fluids involved in IOP and/or POAG (like TM, CB, AH, retina, or optic nerve). Examples of these entries are RHO [21], AQP1 [22] and LTBP1 [23].

The aim of this study was to generate the gene expression profiles and functional annotations of the human NPE and PE cells of the CB, using microarrays. Our interpretation of these data was guided by the major functionalities previously described for the CB, being development, neural nature, endocrine and metabolic functions as well as immunological functions. Previous molecular analysis of the entire CB, using a range of techniques, provided some insight in the expression profile and functionalities of the two epithelia. However, a comprehensive analysis of gene expression and functional annotation of two epithelial layers of the CB was not available yet. In this study, we also determined molecular signatures of the NPE and PE and, finally, we analyzed the expression of genes previously implicated in glaucoma.

## Methods

### Ethics Statement

This study was performed in agreement with the declaration of Helsinki on the use of human material for research. Human donor eyes were provided by the Corneabank Beverwijk, the Netherlands. In accordance with Dutch law, the Corneabank ensured none of the donors objected to the use of their eyes for scientific purposes. Approval of the medical ethics committee was not required as the data were analyzed anonymously.

### Human Donor Eyes, Tissue Processing and Cell Sampling

We selected seven eyes following previously published strict protocols [24]. We now selected adult donors as young possible, to avoid possible undiagnosed POAG [25]. Table S1 outlines the characteristics of the seven donor eyes. The medical histories revealed no prolonged disease state or medication use that could influence RNA expression or quality. Furthermore, the medical histories displayed neither glaucoma or other eye diseases nor any malignancies. Inspection of the eyes with light microscope revealed no abnormalities. Globes were enucleated 10–21 hours post mortem and were frozen within 24 hours total post mortem time.

After removal of the cornea, the donor eyes were transferred to our department and visually inspected. Subsequently, they were snap frozen in isopentane in liquid nitrogen and stored at minus 80°C. Next, twenty 20 µm cryosections of the CB were cut and mounted on slides covered with 1.0 mm PEN-membrane (PALM Carl Zeiss, MicroImaging GmbH, Munich, Germany). The slides were dehydrated and stained with Cresyl Violet (1 gram of Kresyl Violett (Merck, Frankfurt, Germany, art.5235), added to 1 L of 100% ethanol). Cresyl Violet staining was necessary to distinguish NPE. Next, the NPE and PE were separately isolated with a laser microdissection system (PALM Carl Zeiss, Microlaser Technologies AG, Germany) and captured in adhesive caps (AdhesiveCap 500 opaque, Carl Zeiss, Germany). Figure S1 shows the various steps.

### RNA Isolation and Amplification

Total RNA was isolated using RNeasy Micro Kit (Qiagen Benelux, Venlo, The Netherlands). Subsequently, the mRNA component was amplified with Amino Allyl MessageAmp II aRNA Amplification kit (#1753, Ambion Applied Biosystems Europe B.V.). Quantification of tRNA and aRNA was carried out with nanodrop measurement (NanoDrop 1000, Isogen Life Science B.V., the Netherlands) and the quality was checked with a BioAnalyzer assay (Agilent Technologies, Amstelveen, the Netherlands, RNA 6000 Pico Kit). RNA integrity numbers (RIN’s) of tRNA ranged from 6.5 to 9.2 and the peak of the fragment length of aRNA samples varied between 700 and 900 nt. Next, all experimental aRNA samples were labeled with a Cy5 fluorescent probe (Cy5 mono-reactive dye pack, GE Healthcare UK, Little Chalfont, Buckinghamshire, UK). In short, aRNA was coupled to Cy5 (dissolved in DMSO) in 0.5×M NaHCO_3_ (pH 9.0) for one hour. Free dyes were quenched by the addition of 4×M hydroxylamine and removed by filtration over a Chroma Spin-30 column (DEPC-H20 Columns, Clontech Laboratories) with glycogen as a carrier. Incorporation and yield were measured with Nanodrop on a microscale spectrophotometer (Isogen Life Science B.V., the Netherlands). In our microarray studies, we used a common reference design. As common reference sample we used RNA from human RPE/choroid that was used in all previous and ongoing gene expression analyses in our lab [24], [26], [27]. The common reference sample was amplified and labeled with Cy3 (Cy3 mono-reactive dye pack, GE Healthcare UK, Little Chalfont, Buckinghamshire, UK), following the previously described protocols.

### Microarray Hybridization

Seven NPE RNA vs. common reference sample RNA and seven PE RNA vs. common reference sample RNA hybridizations were carried out using catalogue human 4×44×k microarrays (Agilent Technologies, Amstelveen, the Netherlands). Hybridizations, washings and scanning were essentially performed according to the Agilent protocol for hybridization.

### Data Analysis: Gene-expression, Sub-datasets and Functional Annotation

After hybridization, the microarray image files were analyzed and processed by Agilent Feature Extraction Software (Agilent Technologies). Further data processing was performed with the computer programs R (version 2.14.0 for Windows, R Development Core Team, 2009), Windows Excel® (2007 Microsoft, Redmond, WA, USA) and the Ingenuity Knowledge Base (Ingenuity® Systems, version 11631407 (date of analysis June 20 2012), www.ingenuity.com). In R, we normalized the mean expression intensity data against the common reference sample [28], [29]. The raw data and the normalized data can be found in the GEO database (GSE37957).

Next, we prepared different sub-datasets for further specific analyses.

For the first pair of sub-datasets, we sorted the gene expression data of the NPE and PE according to the mean expression intensity (µ-int) [24]. We used the 90^th^, 50^th^ and 10^th^ percentile of the µ-int to categorize our data into groups with high (>90^th^), moderate (50^th^-90^th^), low (10^th^-50^th^) and very low/absent (<10^th^) expression. We focused our analysis on the gene group with high mean expression (µ-int >90^th^ percentile) which reflects the molecular machinery of at least a number of important features [24]. We analyzed the NPE and PE separately and made a comparison between the two cell layers. We called these two sub-datasets the *Highly Expressed NPE and PE sub-datasets*.

For the third sub-dataset, we investigated the differences between NPE and PE by making a statistical comparison with a paired t-test between the expression values of NPE and PE (R package LIMMA, including Bayesian statistics) [30]. Cut-off value for statistical significant difference was a p-value of less than 0.01 after Benjamini-Hochberg correction for multiple testing. The new sub-dataset contained all genes that differed statistically significant between NPE and PE, the so called *Significantly Different Expressed sub-dataset*. We made the comparison of PE versus NPE, so the entries with positive fold changes were higher expressed in PE, while the entries with negative fold changes were higher expressed in NPE. Since it is difficult to attribute specific biological functions in a set of up- or down regulated genes to a specific layer of NPE and PE, we chose to functionally analyze the total *Significantly Different Expressed sub-dataset* in Ingenuity. In this way, we obtained information about differences in functionalities between NPE and PE.

The three sub-datasets were functionally analyzed using the knowledge database IPA (Ingenuity® Systems, version 11631407 (date of analysis June 20 2012), www.ingenuity.com). Ingenuity is a knowledge database bioinformatics program. An Ingenuity ‘core analysis’ typically generates biological functions, canonical pathways and functional molecular networks pertaining to the dataset in question. This analysis takes into account the number and type of genes that the dataset contains, and is based on all available relevant gene, RNA, protein and functional data in the literature and on the World Wide Web. We set the relevant statistical parameters in Ingenuity to Benjamini-Hochberg multiple testing correction and statistical significance was set from p-values <0.01. We first selected the statistically significant biological functions and canonical pathways and grouped these into the most important functionalities of the NPE and PE. We also analyzed the functional molecular networks that were built by Ingenuity. Networks are collections of interconnected molecules assembled by a network algorithm. In other words, it is a graphical representation of molecular and functional relationships. Ingenuity designs molecular networks based on previously published data and also assigns the most important functionalities to these networks. Therefore we call them functional molecular networks. We set Ingenuity to build networks with maximum 35 molecules and a maximum of 25 networks. Sometimes, Ingenuity adds a number of extra molecules from the knowledge database to accomplish a more complete and logical molecular network.

Next, we determined a molecular signature for the NPE and PE. For this, we took the *Significantly Different Expressed sub-dataset* and selected the genes with a fold change >2.5 for the NPE versus the PE, or vice versa, according to previously published methodology [26]. We assumed that these genes represent local cell-specific markers for the NPE or PE.

Finally, we looked for CB expressed genes previously attributed to glaucoma. Using a variety of WWW sources (Pubmed, Uniprot, Ingenuity), we explored the presence of genes previously implicated in glaucoma in the *Highly Expressed NPE and PE sub-datasets* and *Significantly Different Expressed sub-dataset.* Genes that differed statistically significant between NPE and PE, but had a low expression level (<50^th^ percentile), were not selected. Subsequently, we subdivided these genes in three groups: (1) genes with genetic association with glaucoma, (2) genes with changed tissue expression in glaucoma and (3) genes associated with secondary glaucoma or used in animal models for glaucoma.

### Conformation of Microarray Results

To validate and confirm our microarray data, we initially searched the literature for genes already known to be specifically expressed in the NPE and/or PE. We used PubMed (www.ncbi.nlm.nih.gov/pubmed/) with the terms ‘ciliary body’, ‘gene’, ‘expression’, ‘immunohistochemistry’, ‘immunolocalization’, ‘immunofluorescence’, ‘pigmented epithelium’ or ‘non pigmented epithelium’ in our search criteria. We only selected those articles in which immunolocalization provided reliable information about the layer (NPE and PE) where the protein is or is not (preferentially) expressed (see Table S2).

Next, we performed a semi-quantitative RT-PCR of 10 genes that differed statistically significant between PE and NPE, with higher expression in NPE and on 10 genes with statistically significant difference between PE and NPE, with higher expression in PE. The procedure of the sQRT-PCR was previously described by Booij et al. [26]. In short, sQRT-PCR was carried out using intron spanning primers on cDNA from laser dissection microscopy derived samples of NPE and PE. Since we worked with human post-mortem donor material which consists of intrinsic shorter and partly degenerated RNA fragments, we could not use catalogues RT-PCR assays, and we had to generate primers near the 3′ end of the gene. Because of this sub-optimal primer design, PCR was not always optimal and some cDNA’s showed no PCR product at all. Primer sequences used are available on request.

Finally, we performed immunohistochemistry of selected targets. Selection was based on both high RNA expression in NPE and PE and presence in a statistical significant biological or canonical pathway. We selected two entries for every main specific functionality of the CB, relevant in the context of this study (progenitor, neural, endocrinological, and immunological). FGFR3 and NOTCH1 immunostainings were performed to highlight the developmental properties of the CB. To highlight the neural nature of the CB, we stained for ROBO1 and GRIN2C.

For the endocrine signaling of the CB, we selected EDNRB and MNAR. Finally, we selected TLR3 and SERPING1 proteins to demonstrate some of the immunological properties of the CB. All primary antibodies were purchased from Abcam (Cambridge, UK). The total list is presented in Table S3. The protocol for immunostaining was essentially as follows: Fresh frozen CB tissue was cut in 8 µm cryosections and mounted on polylysine coated slides. We mounted an experimental and a control section on the same slide. After drying 1 hour at room temperature, sections were fixated with either acetone or PFA (see Table S3). The primary antibodies were incubated for 90 min at room temperature or overnight at 4°C (Table S3) on the experimental section. The control sections were incubated with the same solution, time and temperature, but without the primary antibody. After incubation the slides were washed in PBS for 20 min. Next, the secondary antibody was incubated on both experimental and control sections for 60 min. The concentrations and types of secondary antibodies per primary antibody are summarized in Table S3. After 60 min, the slides were washed in PBS for 20 min. Then, sections were mounted in Vectashield with Dapi. Fluorescent images were taken with an Axioplan2 microscope (Carl Zeiss, MicroImaging GmbH, Munich, Germany).

## Results

### 1.a. Gene Expression and Functionalities of the NPE

The mean gene expression values of the NPE after normalization ranged from 2.09 to 18.88 (log2 transformed absolute expression levels). These data can be found in the GEO database (GSE37957).We selected the highly expressed genes, which mean expression levels ranged from 13.70 to 18.88. In total, 2457 genes were highly expressed in the NPE after subtraction of technical replicates and we called this the *Highly Expressed NPE sub-dataset.* We functionally analyzed this sub-dataset in Ingenuity. The knowledge database program assigned 52 statistically significant biological functions, 23 canonical pathways and 25 functional molecular networks to this dataset. The total list of genes of this sub-dataset is given in Table S4.

#### Biological functions of the NPE

The list of statistically significant biological functions is given in Table S5. We subdivided the biological functions in five major categories. The first category contained developmental functionalities, like tissue development, embryonic development and hereditary disorders. In the second category, we assembled neurological diseases, for example Huntington’s and Alzheimer’s disease and tauopathies. The third category consisted of endocrine and metabolic functionalities, such as metabolism of nucleic acid and energy production, as well as metabolic disorders, such as experimentally induced diabetes and iron overload. In the fourth category, we assembled different immunological functionalities and related pathologies, like inflammatory response, autoimmune disease and arthritis. Finally, the fifth category contained the remaining and less specific functions such as basic cellular functions, protein synthesis, cell cycle and cancer.

#### Canonical pathways of the NPE

**Figure 2:**
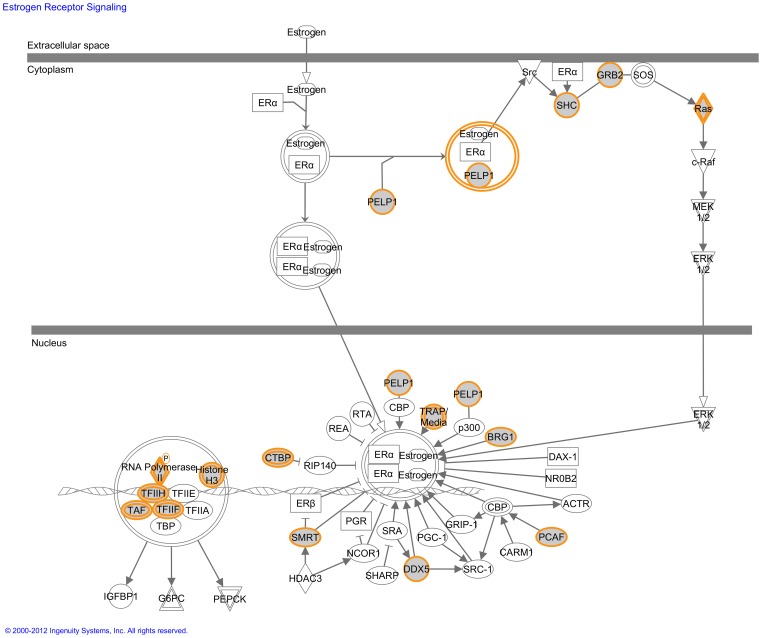
The estrogen receptor signaling pathway identified by the Ingenuity software. This is one of the canonical pathways that contained statistically significantly more genes than expected by chance in the group of genes that are highly expressed in the non-pigmented epithelium (NPE) of the ciliary body. Gray fields indicate their presence among the highest expressed genes of the NPE; uncolored genes are added by the Ingenuity software to form the pathway. Solid lines between molecules indicate direct physical relationships between molecules (such as regulating and interacting protein dom;ains). Abbreviations of gene names are according to standard abbreviations used in Genbank. This pathway is an example of the endocrine signaling pathways found in the NPE.

The list of statistically significant canonical pathways is presented in Table S6. We subdivided all these pathways in different functional themes, which in part overlap with the biological functions generated above. We did not find any canonical pathway within the first theme of developmental cell properties in the NPE. The second theme concerned neurological function and disease and we found the canonical pathway ‘Huntington’s disease signaling’ (Figure S2). Third, we observed a large number of significant endocrine signaling pathways. They included ‘Estrogen receptor signaling’ (Figure 2), ‘Androgen signaling’, ‘Glucocorticoid receptor signaling’ and ‘ Ephrin receptor signaling’ (respectively Figures S3–5). We also found some pathways concerning metabolism, like ’Glycolysis/gluconeogenesis’ (Figure S6). The fourth theme included several significant canonical pathways in immunological and inflammatory process, like the ‘Antigen presentation pathway’ (Figure S7) and ‘IGF-1 signaling’ (Figures S8). Finally, we found three pathways concerning oxidative stress mechanisms, namely ‘Oxidative phosphorylation’, ‘NRF2-mediated oxidative stress response’ and ‘Hypoxia signaling in the cardiovascular system’ (Figures S9 and S10). In general, these pathways may be of interest, since there is increasing evidence that oxidative stress in the CB occurs under normal physiological conditions and accumulates during the development of glaucoma [31].

#### Molecular networks of the NPE

The list of functional molecular networks produced by Ingenuity for the NPE is given in Table S7. Again, we subdivided the molecular networks according to four major NPE functionalities, namely development, neural nature, metabolic and endocrine signaling, and immunological properties, and a rest group containing ‘basic cellular (dys)functions’.

We observed genes related to developmental cell properties in four functional molecular networks (9, 10, 16, and 21). Network 16 (Figure S11) was build around the FOS gene in the center. FOS proteins were implicated as regulators of cell proliferation, differentiation and transformation during development [32]. Other genes involved in developmental processes were MEIS2, MAML1 and FOXP1 (network 9; Figure S12); FGFR1, FGFR2, SFRP1 and CCNH (network 10; Figure S13) and AES and SIX6 (network 21; Figure S14).

**Figure 3:**
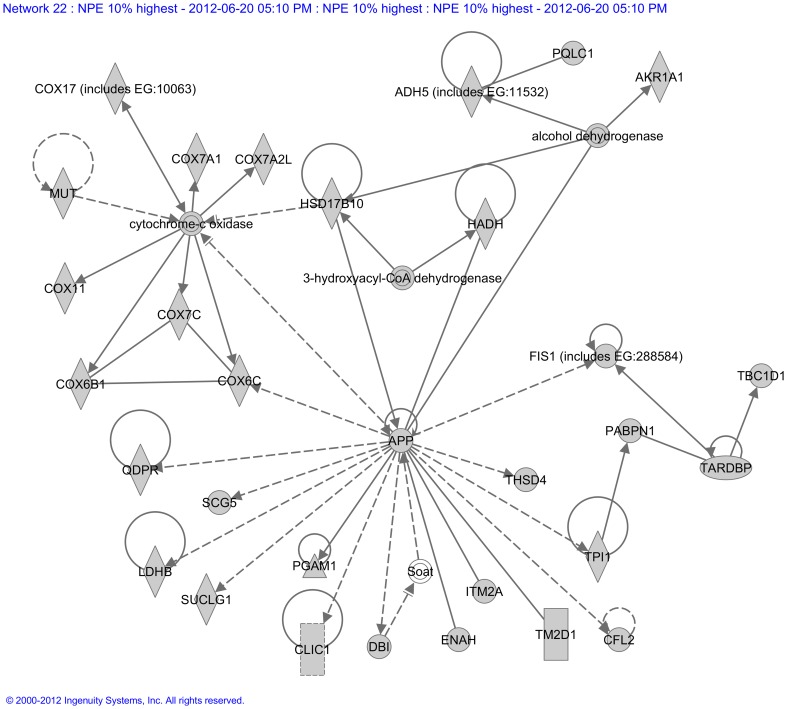
Molecular network generated by the Ingenuity software from the highest expressed genes of the non-pigmented epithelium (NPE). Molecular network generated from our microarray *Highest expressed NPE sub-dataset*. Grey symbols represent genes that are highly expressed in the NPE of the ciliary body. Transparent entries are molecules from the knowledge database, inserted to connect all relevant molecules in a single network. Solid lines between molecules indicate direct physical relationships between molecules (such as regulating and interacting protein domains); dotted lines indicate indirect functional relationships (such as co-regulation of expression of both genes in cell lines). Abbreviations of gene names are according to standard abbreviations used in Genbank. The main functionalities given by Ingenuity for this molecular network are ‘Metabolic disease, cellular assembly and organization, cell death’. This network contained the gene APP and TM2D1. Both genes code for proteins involved in amyloid beta process, involved in neuronal cell death and Alzheimer’s disease.

Next, five networks (11, 13, 20, 22 and 24) pointed to the neuronal nature of the NPE. Network 11 (Figure S15) contained the gene MAP1LC3B, whose protein product is involved in neurogenesis. Also three genes coding for GABA receptor associated proteins were present in this network, commonly mediating inhibition of neurotransmission and receptor sorting and targeting. In Network 13 (Figure S16), we observed the UCHL1 gene, which is a neuron specific gene, previously implicated in Parkinson’s disease. Network 20 (Figure S17) contained several genes that were previously associated with neurological diseases. For example: Huntington’s disease (NDUFA7, NDUFC1, NDUFB2, NDUFB3, NDUFA8) [33], [34], atrophy of optic nerve (NDUFS4) [35] and Leigh syndrome (NDUFS7, NDUFA2, NDUFS4) [36]–[38]. Network 22 (Figure 3) contained the gene APP and TM2D1. Both genes code for proteins involved in amyloid beta processing, which cause neuronal cell death and Alzheimer’s disease. Network 24 (Figure S18) contained also several genes previously associated with neurological diseases, like ZFYVE27 (hereditary spastic paraplegia) [39], ATB1B1 (neurodegeneration of photoreceptors) [40] and SPG21 (Mast syndrome) [41]. Also, genes involved in nervous system development were found, for example ATP1B1 (involved in aggregation of neurons), TRIM2 (neuroprotection of cortical neurons) and VAPA, SGK1 and ZFYVE27 (involved in morphogenesis of neuritis).

Thirdly, we identified several networks (7, 17, and others) related to metabolic and endocrine signaling functionalities. For example Network 7 (Figure S19), which contained several genes involved in metabolic pathways, like LPL and CYP1B1 (lipid metabolism), IDH1 (energy production), GSTM3, GSTP1, GSTO1 and MT2A (detoxification and drug metabolism), SLC23A2 (transport of vitamin C) and SLC39A8 (zinc transport). Network 17 (Figure S20) contained several subtypes of aldehyde dehydrogenase (ALDH1A1, ALDH1A3, ALDH4A1, ALDH6A1 and ALDH9A1) that are involved in alcohol, retinol and lipid metabolism. The other networks also contained interesting endocrine and/or metabolic genes, namely FOLR1 (folate receptor), PPAP2A (de novo synthesis of glycerolipids) and SLC19A2 (thiamin transporter), NR3C1 (glucocorticoid receptor) and SLC38A1 (glutamine transporter) and the gene ESRRA (estrogen-related receptor). Finally, we found two functional molecular networks (21, 25) that contained genes involved in immunological process. Network 21 contained the gene TNFRSF14, which protein is involved in viral entry mechanisms. Network 25 (Figure S21) contained several genes which protein products are involved in immunosuppressive process and HIV infection, namely PPIA, PPIB, FKBP3 and FKBP9. The rest group contained several networks involved in cytoskeleton and extracellular matrix assembly, ribosomal regulations and mitochondrial process. Of interest, we found several genes previously associated with glaucoma in the molecular networks. These genes were TMCO1 (Network 6; Figure S22), OPTC (Network 9; Figure S12) and LTBP2 (Network 11; Figure S15).

### 1.b. Gene Expression and Functionalities of the PE – Similarity with the NPE

We analyzed the PE gene expression data in the same way as the NPE gene expression (described above). The total mean expression values ranged from 1.80 to 18.82 (log2 transformed absolute expression levels). These data can be found in the GEO database (GSE37957). There were 2458 genes highly expressed in the PE (after subtraction of technical replicates), the so called *Highly expressed PE sub-dataset.* The mean expression level of these highly expressed genes ranged from 13.71 to 18.82 (log2 transformed absolute expression levels). The total list of genes of this sub-dataset is given in Table S8.

We compared the *Highly expressed PE and NPE sub-datasets* with each other and found a high similarity: 88% of the entries overlapped. Analysis of other gene expression groups, as defined by their percentile (see Methods) in both NPE and PE yielded similar results (not shown).

Again, we also conducted a functional analysis on the *Highly expressed PE sub-dataset* in Ingenuity. Since the gene expression datasets of the PE and NPE were very similar, it was not an unexpected finding that the molecular and functional annotations generated by Ingenuity were also highly comparable (Tables S5 and S6). Again, we found the four major themes, namely developmental functionalities, neurological background, endocrine and metabolic pathways, and immunological properties. The total list of functional molecular networks of the PE is given in Table S9.

### 2. Differences between PE and NPE

We examined *the differences* between NPE and PE, and conducted a statistical comparison between the mean expression values of the PE and NPE (see Methods section). In total, 1576 out of the 19,596 genes were statistically significantly different expressed between the NPE and PE cell, the so called *Significantly Different Expressed sub-dataset*. Within this list, 360 entries are higher expressed in the NPE compared to the PE and 1216 entries higher in the PE compared to the NPE. In Table S10, the total list of genes of this sub-dataset is given.

Next, we functionally annotated the whole *Significant Different Expressed sub-dataset* in Ingenuity. Ingenuity assigned 74 statistically significant biological functions, 23 statistically significant canonical pathways and 25 functional molecular networks to be different between NPE and PE.

#### Biological functions different between NPE and PE

We subdivided the statistically significantly different biological functions in major categories, which resulted in the same five major categories (development, neural nature, neuro-endocrine, immune privilege, and a rest category) as described in the *Highly Expressed NPE and PE sub-datasets* (Table S11). We found a variety of developmental functionalities which were different between NPE and PE. These included embryonic development, developmental disorders and development of, amongst others, visual, nervous and renal and urological system. In the second category, of neural function and disease, we found (N)PE differences in molecular process related to neurodegenerative disorders, schizophrenia and neuromuscular disease. In the third category, endocrine and metabolic (dis)functions, we found functions within metabolism of lipid, carbohydrate, drug, nucleic acid, vitamins and minerals. In the fourth category, we found a variety of immunological functions that were different between NPE and PE. For example, immune cell trafficking, antigen presentation, entrance of virus and autoimmune diseases. In the fifth category, we grouped all remaining functionalities. Many of these were basic cellular functions, comparable with those that we found in the *Highly Expressed NPE and PE sub-datasets*. Additionally, we found functionalities regarding cardiovascular and hematological disease, including arteriosclerosis, hypertension, hypertrophy and cyanosis.

#### Canonical pathways different between NPE and PE

Functional annotation of our dataset suggested that 23 canonical pathways differed significantly between NPE and PE (Table S12).

**Figure 4:**
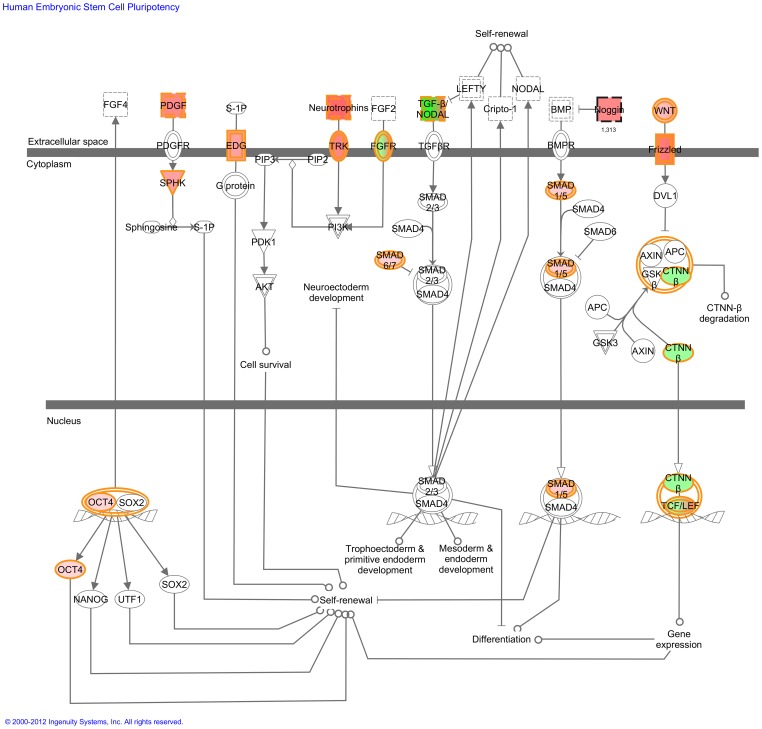
Human embryonic stem cell pluropotency pathway identified by the Ingenuity software. This is one of the canonical pathways that contain statistically significantly more genes than expected by chance in the group of genes that differ statistically significantly between non-pigmented (NPE) and pigmented epithelium (PE). Red fields indicate genes statistically significantly higher expressed in PE compared to NPE whereas green fields indicate genes statistically significantly higher expressed in NPE compared to PE. Fields with both green and red color represent gene groups, of which some genes are significantly higher expressed in NPE (green) and other in PE (red). Uncolored genes are added by the software to form the pathway. Solid lines between molecules indicate direct physical relationships between molecules (such as regulating and interacting protein domains). Abbreviations of gene names are according to standard abbreviations used in Genbank.

For example, with regard to progenitor cell properties (Theme 1), we found that the pathway of ‘Human embryonic stem cell pluripotency’ (Figure 4) and ‘Wnt/β-catenin signaling’ (Figure S23) differed significantly between the (N)PE. Within the second theme, neural function and disease, three canonical pathways were found. These were ‘Axonal guidance signaling’, ‘Glioblastoma multiforme signaling’ and ‘Neuropathic pain signaling in dorsal horn neurons’ (Figures S24 and S25). Thirdly, there were eight canonical pathways pointing to different endocrine and metabolic functionalities between NPE and PE, including ‘Endothelin-1 signaling’ (Figure S26), ‘Dopamine-DARPP32 feedback in cAMP signaling’ and ‘Phospholipid degradation’. Also the canonical pathway ‘Cellular effects of sildenafil (Viagra)’ was found (Figure S27). Sildenavil is a vasodilator agent and oral administration in sheep resulted in an increased AH production and increased IOP [42]. Immunological differences (Theme 4) included the canonical pathways ‘Antigen presentation pathway’ and ‘Hepatic fibrosis/hepatic stellate cell activation’ (Figure S28). We also found two additional interesting canonical pathways that differed between NPE and PE, namely ‘Caveolar-mediated endocytosis signaling’ (Figure S29) and ‘Gap junction signaling’ (Figure S30). Finally, we found several basic cellular pathways of cellular (dis-)function related to cancer.

#### Molecular networks different between NPE and PE

**Figure 5:**
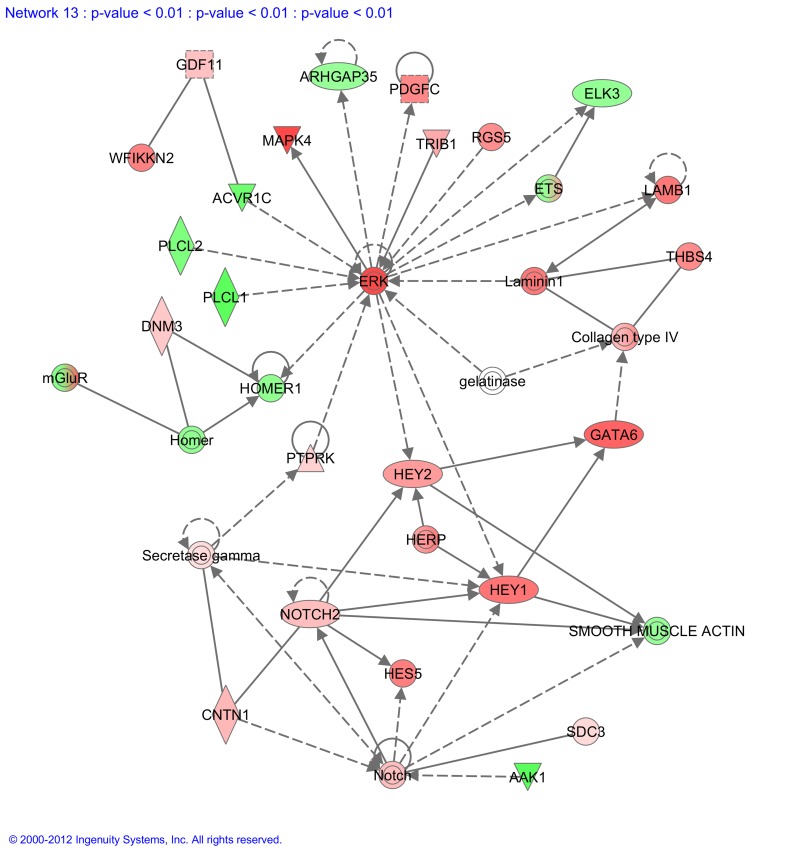
Molecular network generated by Ingenuity software from the statistically significantly different expressed genes. Molecular network generated from our microarray *Significantly Different Expressed sub-dataset.* Red fields indicate genes statistically significantly higher expressed in PE compared to NPE whereas green fields indicate genes statistically significantly higher expressed in NPE compared to PE. Solid lines between molecules indicate direct physical relationships between molecules (such as regulating and interacting protein domains); dotted lines indicate indirect functional relationships (such as co-regulation of expression of both genes in cell lines). Abbreviations of gene names are according to standard abbreviations used in Genbank. The main functionalities given by Ingenuity for this molecular network are ‘Connective tissue development and function, skeletal and muscular system development and function, cardiovascular disease’. Highlight in this network is the presence of many genes that are involved in developmental process, namely NOTCH2, GATA6, HEY1, HEY2 and GDF11.

The functional molecular networks different between NPE and PE are summarized in Table S13. We found several molecular networks (4, 12–14) with functionalities related to development. For example Network 13 (Figure 5) contained the genes NOTCH2, GATA6, HEY1, HEY2 and GDF11, all important during development. Also Network 14 (Figure S31) contained several genes known to be involved in embryonic development, namely genes SOX8, SOX11, MSX1, FRZB and WNT2B. Network 4 (Figure S32) contained the gene SIX6, whose protein product is involved in eye development and Network 12 (Figure S33) contained the gene CLRN1 that is involved in development of the retina. The genes SIX6 and CLRN1 are higher expressed in NPE compared to PE, whereas the other genes described were all statistically significantly higher expressed in PE compared to NPE.

We also found molecular networks (1, 15, 16) containing neural features different between the NPE and PE. In Network 1 (Figure S34) we found centrally the gene DISC1. The protein of this gene is involved in neurite outgrowth and cortical development. Also, this gene is associated with schizophrenia. The gene DST is involved in transport of axons and coalignment of neurofilaments and the gene OLFM1 is neural tissue specific. In Network 15 (Figure S35) we found two genes that were previously associated with neurodegeneration, namely PTGS2 and PSEN1. The protein products of PSEN1 and ABCA1 are involved in amyloid-beta metabolism. Moreover, the genes NOS1 and PTGS2 have been associated with glaucoma. In Network 16 (Figure S36) we found the genes GRIN2C and GRIN3A, both coding for NMDA glutamate receptors that are important for neural signaling, and the genes EPH1, EPH2 and FOXP2, that are involved in morphogenesis of the eye.

Next, we observed neuro-endocrine signaling and metabolic differences between NPE and PE in several networks (3, 20). Network 20 (Figure S37) contained several molecules involved in lipid metabolism, namely APOE, LDL, OSBP and ELOVL3. Moreover, we found a gene in this network involved in drug metabolism (CES1) and glucose transport (SLC2A2). Network 3 (Figure S38) was constructed with many G-coupled receptors that differed statistically significant between NPE and PE. G-coupled receptors are activated by extracellular stimuli, like neurotransmitters, hormones and light, and activate intracellular process. Apparently, some of these receptors differ between NPE and PE, for example GRM1 and GRM8 (glutamate receptor), ADRA2A (adrenergic alpha receptor), HTR2C (serotonin receptor), GPER (estrogen receptor) and EDNRB (endothelin B receptor).

Subsequently, we found immunological network functionalities (2, 19), that differed between NPE and PE. In Network 2 (Figure S39) we found several genes whose protein products are involved in immunological process, including IFNG, HAVCR2 and KLRB1 (activation of macrophages and natural killer cells), CD48 and CRTAM (T-cell mediated immune response), HCP5 and HLA-DQ (HLA complex subtypes). In Network 19 (Figure S40) we identified the genes STAT4 whose protein product mediates responses to IL12 lymphocytes and regulate the differentiation of T-helper cells. Moreover, this network contained the gene TLR3 (fundamental role in pathogen recognition and activation of innate immunity), IGSF21 (an immunoglobulin) and CD2 and CD69 (activation of T-lymphocytes).

Finally, we identified several networks that contained genes coding for cytoskeleton and matrix assembly proteins that seem to differ between NPE and PE. For example Network 6 (Figure S41) contained genes coding for cadherin (CDH6, CDH11), catenin (CTNNA2, CTNND2), collagen (COL13A1, COL15A1) and extracellular matrix protein (ECM1).

### 3. Molecular Signature of NPE and PE

We found three genes locally specifically expressed by the NPE, namely CDR1, A_24_P530977 and PDE11A (Table 1). CDR1 with systemic name AK054921 is a cDNA clone highly similar to 40 s ribosomal protein S15A. Moreover, the CDR1 gene codes for the cerebellar degeneration-related antigen 1 protein, which is involved in auto-immune diseases. A_24_P530977 codes for a cDNA fragment with yet unknown function. PDE11A codes for a phosphodiesterase that catalyze the hydrolysis of cAMP and cGMP.

**Table 1:** Molecular Signature of the NPE.

Gene Name	Systemic Name
A_24_P530977	A_24_P530977
CDR1	AK054921
PDE11A	NM_016953

For the PE, we found 143 locally specifically expressed genes (Table 2). Around forty genes coded for cDNA clones with unknown functions or predicted genes. Also, around forty additional genes coded for proteins involved in a broad variety of basic cellular functions. Data driven, we identified six PE genes whose protein products were involved in pigmentation (DCT, GPNMB, MLANA, SERPINF1, TYR and TYRP). Furthermore, we found fourteen genes involved in cytoskeleton and extracellular matrix assembly, namely CTRP5, CHRDL2, CTSK, GJA4, HS6ST2, ITGAV, KAZALD1, LAMB1, MYLK, PCDH8, PCOLCE2, PDLIM3, SOSTDC1 and SPON1. We found sixteen genes coding for proteins involved in (neuro)endorcrine and metabolic process. Interestingly, these included AGT and CPA6 that are involved in blood pressure regulation and CALCA whose protein product induces vasodilatation. Possibly these entries are related to regulation of the aqueous humor production. Also we found a gene coding for the gastrin-releasing peptide (GRP) that might have neuro-regulatory functions, a gene coding for follestatin (FST) that inhibits FSH release, a gene involved in thyroid hormone synthesis (DUOX1) and one in prostaglandin biosynthesis (PTGS2). Furthermore, we identified genes coding for a receptor of glutamate (GRM8) and serotonin (HTR2C). Genes coding for proteins involved in metabolic processes included, ABCA1, ABCC1, LIPI and LPL (lipid metabolism), MOCOS (xanthine and aldehyde metabolism), SLC23A2 (vitamin C uptake) and SLC40A1 (iron uptake). Finally, we identified seventeen genes whose proteins play a role in embryonic and developmental process. These were FRZB, GDPD5, HES5, HEY1, HEYL, ID2, IRX3, LMO2, MDK, MSX1, MSX2, MSX2P, SP5, TDRD9, TFEC, TLL1 and TNFRSF19. In conclusion, compared to the NPE, the PE expressed many specific genes. The majority of the proteins that they are coding for play a role in pigmentation, cytoskeleton and extracellular matrix assembly, (neuro)endocrine and metabolic process and developmental pathways.

**Table 2:** Molecular Signature of the PE.

Gene Name	Systemic Name	Gene Name	Systemic Name	Gene Name	Systemic Name
A_23_P140454	A_23_P140454	GJA4	NM_002060	NTRK3	NM_002530
A_23_P421323	A_23_P421323	GNA14	ENST00000341700	P2RY6	NM_176798
A_24_P910504	A_24_P910504	GNG11	NM_004126	PAQR9	NM_198504
A_32_P171043	A_32_P171043	GNGT1	NM_021955	PCDH8	NM_002590
A_32_P178241	A_32_P178241	GPM6B	NM_001001996	PCOLCE2	NM_013363
A_32_P68205	A_32_P68205	GPNMB	NM_001005340	PDLIM3	NM_014476
ABCA1	NM_005502	GPR4	NM_005282	PRAP1	NM_145202
ABCC1	NM_019862	GRM8	NM_000845	PRDM16	NM_022114
ACP5	NM_001611	GRP	NM_002091	PRKCB1	NM_002738
AGT	NM_000029	HES5	NM_001010926	PTGS2	NM_000963
AK094786	AK094786	HEY1	NM_001040708	PTPRG	NM_002841
AK094929	AK094929	HEYL	NM_014571	RAB3C	NM_138453
ATP1B1	NM_001677	HS6ST2	NM_001077188	RAMP1	NM_005855
ATP6V1C2	NM_001039362	HTR2C	NM_000868	RBM20	ENST00000369519
B3GAT1	NM_054025	ID2	NM_002166	RP11–301I17.1	NM_017993
BC036599	BC036599	IGF1	NM_000618	RRAD	NM_004165
BC042026	BC042026	IGSF21	NM_032880	SEMA6D	NM_153618
BC047380	BC047380	IRX3	NM_024336	SERPINF1	NM_002615
BM802662	BM802662	ITGAV	NM_002210	SLC23A2	NM_203327
BSPRY	NM_017688	KAZALD1	NM_030929	SLC26A7	NM_052832
BX956036	BX956036	KCNB2	NM_004770	SLC40A1	NM_014585
C10orf107	NM_173554	KCND2	NM_012281	SLC6A15	NM_182767
C21orf15	NM_015645	KIAA0367	NM_015225	SORCS3	NM_014978
C4orf18	AY040090	KLHL2	NM_007246	SOSTDC1	NM_015464
C8orf12	NM_016613	LAMB1	NM_002291	SP5	NM_001003845
C8orf13	BC080558	LGALS3	NM_002306	SPARCL1	NM_004684
CALCA	NM_053279	LGI1	NM_005097	SPON1	NM_006108
CB995946	NM_001033953	LIPI	NM_198996	TACSTD1	NM_002354
CBFA2T3	CB995946	LMO2	NM_005574	TDRD9	NM_153046
CDH13	NM_005187	LOC439949	AY007155	TFEC	NM_012252
CHRDL2	NM_001257	LOC654346	NM_001040078	TGFBI	NM_000358
CPA6	NM_015424	LPL	NM_000237	THC2609493	THC2609493
CPNE5	NM_020361	LRRC3B	NM_052953	THC2643086	THC2643086
CR618615	NM_020939	LRRTM2	NM_015564	THC2675163	THC2675163
CTRP5	CR618615	LTK	NM_002344	THC2693923	THC2693923
CTSK	NM_000396	MAMDC2	NM_153267	TLL1	NM_012464
DCT	NM_001922	MDK	NM_001012334	TMEM132B	NM_052907
DUOX1	NM_017434	MLANA	NM_005511	TMEM176A	NM_018487
FAM129A	NM_052966	MOCOS	NM_017947	TMEM178	NM_152390
FLJ14213	NM_024841	MSX1	NM_002448	TMEM20	NM_153226
FLJ20366	NM_017786	MSX2	NM_002449	TNFRSF19	NM_148957
FLJ39822	NM_173512	MSX2P	NR_002307	TRA@	BC063385
FRMD6	NM_001042481	MTSS1	NM_014751	TRIM9	NM_015163
FRZB	NM_001463	MYLK	NM_053025	TYR	NM_000372
FST	NM_013409	NCALD	NM_001040630	TYRP1	NM_000550
FXYD6	NM_022003	NCF2	NM_000433	VEGFC	NM_005429
FZD10	NM_007197	NFE2L3	NM_004289	WFIKKN2	NM_175575
GDPD5	NM_030792	NTF3	NM_002527		

### 4. (N)PE Expression of Genes Previously Implicated in Glaucoma

In our *Highly expressed NPE and PE* and *Significant different expressed sub-datasets* of the CB epithelia, we observed in total 35 genes that were previously implicated in the genetics or patho-physiology of glaucoma (Table 3). We observed expression of seven genes which were previously genetically associated with POAG (included normal tension glaucoma (NTG)), namely APOE, CAV1, EDNRA, MYOC, OPTC and TMCO1. We also observed expression of eighteen genes, that previously showed differential expression in eye tissues affected by glaucoma, for example increased expression of RHO mRNA in the optic nerve [21], AQP1 in the retina [22] and LTBP1 in ciliary body, trabecular meshwork and aqueous humor [23] (physiological association). We observed (N)PE expression of four genes that (also) cause optic nerve damage in mutated mouse, namely GPNMB, SOD1, TYR and TYRP1.

**Table 3:** Glaucoma genes highly expressed in the ciliary body.

Gene name	Genetic Association	Changed tissue expression	Secondary glaucoma/Animal models
AKAP2		Increased in eye of PEX syndrome [79]	
APOE*	NTG [80]		
APP		Increased in eye in experimental OHT [81]	
AQP1		Decreased in retina and optic nerve in experimental OHT [22]	
BMP7		Increased in eye of glaucoma patients [82]	
CARD10*	Optic disc areas [83]		
CAV1*	POAG [84]		
CDKN2A*	NTG, POAG and optic disc parameters [20], [85], [86]		
CRYAB		Increased in TM in experimental OHT [87]	
CTGF		Increased in AH of POAG patients [88]	
CTSA		Increased in AH of POAG patients [71]	
CYP1B1	Congenital glaucoma [89]		
EDNRA*	NTG [90], [91]		
GJA1*			Oculodentodigital dysplasia with secondary glaucoma [92], [93]
GPNMB*			Mutations in mouse cause pigmentary glaucoma [94]
HSPB1		Increased in retina in experimental OHT [95]	
LTBP1		Increased in CB of glaucoma patients [23]	
LTBP2	Congenital glaucoma [96]		
MGP		Increased in TM of POAG patients [97]	
MYOC	POAG [98]–[100]		
NOS1*		Increased in optic nerve of experimental OHT [101]	
NR3C1			Glaucoma might develop after cortesteroid usage [48]
OPTC	Possible POAG [102], [103]		
PAX6			Aniridia with secondary glaucoma [104]
RHO		Increased in optic nerve of glaucoma patients [21]	
SLC4A4*	Glaucoma [105], [106]		
SOD1		Increased in retina in experimental OHT [107]	Mutations in mouse cause retinal ganglion cell los [108]
TF		Increased in AH of POAG patients [71], [109]	
TGFB1*		Increased in AH of POAG patients [110]	
TGFB2*		Increased in eye of glaucoma patients [111]	
TIMP2*		Increased in optic nerve in experimental OHT [112]	
TMCO1	POAG [85], [86], [113]		
TTR		Increased in AH of POAG patients [71]	Amyloidosis transthyretin-related disorder with secondary glaucoma [73], [74]
TYR*			Mutations in mouse cause glaucoma [114]
TYRP1*			Mutations in mouse cause pigmentary glaucoma [94]

* Gene is statistically significant different expressed between NPE and PE, with medium or high expression level.

Abbreviations: NTG: normal tension glaucoma; POAG: primary open angle glaucoma; PEX: pseudoexfoliation syndrome; OHT: ocular hypertension; CB: ciliary body; TM: trabecular meshwork; AH: aqueous humor.

### 5. Confirmation of Microarray Results

We confirmed in total 80 entries in the literature that matched our criteria (Table S2). Thirty-four entries were examined in human CB; 27 genes/proteins (79%) confirmed our findings and seven proteins (21%) were expressed in possible conflict with our gene expression data. According to the literature, the latter proteins were only detectable in the NPE, whereas our data indicated that at least the RNA was produced in both cell layers.

With semi-QPCR, we confirmed the differential expression pattern between the NPE and PE for 15 out of 15 genes on the microarray. Five additional cDNAs (THC2675163, MAPK4, FAM43B, NR0B1 and CCND1) did not PCR amplify well, even after several tries. Figures S42A and S42B present the results.

**Figure 6:**
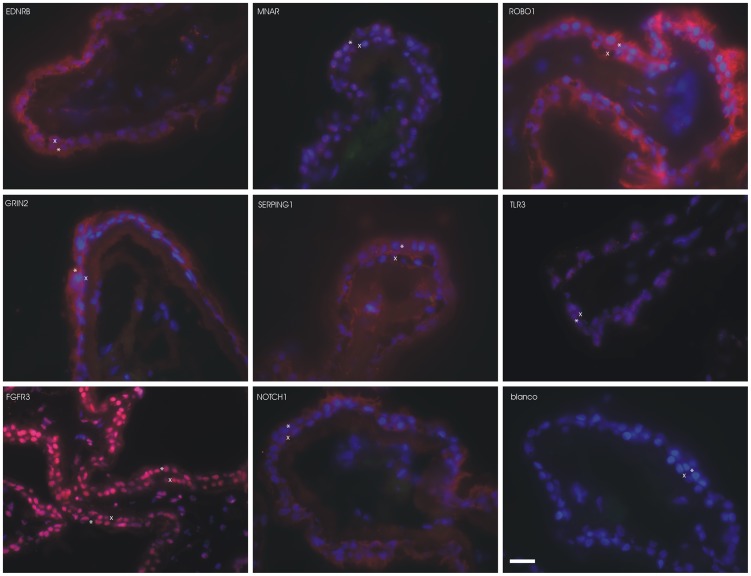
Immunohistochemistry of the ciliary body. Immunohistochemistry of eight proteins involved in the four major functionalities of the ciliary body. For the progenitor cell properties, we stained FGFR3 and NOTCH1. For the neural background, we stained ROBO1 and GRIN2C. We chose EDNRB and MNAR for the endocrine signaling function, and SERPING1 and TLR3 as examples for the immunological functions. Red staining is the secondary antibody with Cy3 that binds to the specific primary antibody, indicated in the figures. Blue staining is Dapi which stains the cell nucleus. Green staining is FITC channel to look for autofluorescence. We found minimal autofluorescence. One negative control (blanco) for ROBO1 is shown. For the other antibodies, the negative controls were also negative (not shown). We found staining for all proteins in both the non-pigmented (NPE) and pigmented epithelium (PE). The white scale bare indicates 20 µm. The symbol * indicates NPE and x indicates PE.

Finally, we performed immunohistochemistry. We could confirm the presence of all eight proteins in the CB. For all proteins, we found staining both in the NPE as in the PE. All negative controls did not stain. In Figure 6, a compilation of all different immunofluorescence stainings is given.

## Discussion

Using microarray studies, we provided a comprehensive analysis and functional annotation of the CB transcriptome, uniquely based on the individual expression profiles of NPE and PE cells and guided by the functionalities previously attributed to the CB (development, neural nature, neuro-endocrinological and metabolic functions and immune/privilege). Besides these functionalities, we also found many pathways in basic cellular functions that were highly expressed in the CB. Since these are not tissue specific, we will not go in detail into these pathways. We started our analysis on the highest expressed genes of the NPE and PE and found that the two cell layers resembled a lot. Yet, we also found differences in gene expression between NPE and PE: 1576 genes were differentially expressed between the two layers. Functional analysis of these differentially expressed genes showed many overlapping functionalities with the *highly expressed sub-datasets* functionalities. While this seems surprising, a most plausible interpretation of our results is that the PE and the NPE have the same basic biological features, which differ in detail. Next, we studied the specific molecular signatures of the NPE and PE. It turned out that especially the PE expressed locally cell-specific genes. Many of them were involved in pigmentation, cytoskeleton and extracellular matrix assembly, (neuro)endocrine and metabolic process and developmental pathways. It is likely that a number of genes mentioned are expressed also to a certain extend somewhere else in cells of the human body, for example in the epithelial cells of the kidney or intestine. However, the similar proteins expressed elsewhere do not, or hardly, affect the local ocular function of the CB. We were therefore interested in locally specific expressed signatures genes.

Finally, we looked also for genes previously attributed to glaucoma. These data give us more insight in the complex etiology of glaucoma and the possible biological mechanisms of these genes in the ciliary body implicated in IOP regulation.

Our microarray approach has several strengths and limitations, as extensively discussed elsewhere [24], [26], [27].

### 1. Functionalities of the Ciliary Body

#### Developmental properties

Our data-driven analysis and functional annotation showed that both the NPE and the PE highly express genes that are involved in (neuro)-developmental processes. Ingenuity derived the statistically significant canonical pathway named ‘Human embryonic stem cell pluropotency’ out of the *Significantly Different sub-dataset* (Figure 4). These findings suggest that the CB has possible (retinal) stem cell properties and that there is a difference in this function between the PE and NPE. We immunostained for NOTCH1 and FGFR3, two proteins that differed statistically in gene expression level between NPE and PE. We were not able to confirm the observed RNA difference on the protein level (Figure 6). However, protein quantification on histological slides is difficult, especially when one cell layer contains melanin granules.

The apparent embryonic stem cell nature of the human CB is of interest for stem cell biologists and visual researchers; and an issue of current literature debate. Although we do not solve that issue with our current study, we do provide molecular clues concerning the apparent differentiation state of the (N)PE in the human adult. Further and more specific investigations are warranted to what extend the human (N)PE contains (properties of) suitable retinal progenitor cells useful for therapeutic purposes.

#### Neural nature

Functional annotation of our expression data yielded multiple clues which point to the neural nature of the human CB. Several genes involved in neural functions and pathologies are highly expressed in the NPE and PE and multiple biological pathways concerning neural functions were found with Ingenuity. Protein expression of two of these genes was confirmed with immunohistochemistry (namely ROBO1 and GRIN2C, Figure 6). Indeed, both CB epithelia are derived from the neural ectoderm during development, so these observations may not be surprising. Interestingly, we also found many genes highly expressed in the CB that have been previously associated with age-related neurological diseases. To our knowledge, the disease genes and corresponding neurological disorders (Alzheimer, Huntington and Parkinson disease), do not directly affect aqueous humor production. However, some clinical studies do show an increased incidence of glaucoma in patients with Alzheimer or Parkinson’s disease [43]–[46]. We did not find studies showing potential associations between Huntington’s disease and glaucoma. This might be explained by the early onset of symptoms in Huntington's disease compared to the onset of glaucoma, hampering the diagnosis of glaucoma.

#### (Neuro-) endocrine function

We found in our data that the NPE and PE highly express many endocrine receptors. The most striking are receptors for glutamate, GABA, serotonin, leptin, glucocorticoids, estrogen, endothelin A and B, folate and adrenergic alpha. Indeed, the signaling pathways related to these receptors were, in part, functionally annotated by Ingenuity on the basis of our CB expression data. To further highlight the endocrine signaling functions of the CB, we immunostained two receptors: EDNRB and MNAR. Our expression data also showed high expression of different (neuro)-endocrine molecules, including MYOC (myocilin), GRP (gastrin-releasing peptide), TF (transferrin), TTR (transthyretin), AGT (angiotensinogen) and different growth factors (amongst others IGF2; IGFBP2,-5,-6,-7; FGF3,-13; FGFBP2; VEGFB, VEGFC; TGFB1–3).

These endocrine receptor and neuro-endocrine molecules in the CB could play a role in regulating the IOP by influencing the AH production, or the TM outflow facility. Indeed, a number of previous literature studies addressed this hormonal regulation of IOP. Kas and Sears found that the administration of estrogen and progesterone leads to a decrease in IOP whereas the administration of testosterone yields an increase in IOP [47]. Furthermore, glucocorticoid use leads to ocular hypertension in certain people, and a high percentage of POAG patients has an increased cellular sensitivity to glucocorticoids (so called ‘steroid responders’) [48]. It is well known that the increased IOP after steroid use is due to decreased outflow of the aqueous humor through the TM [49], [50]. However, also the CB contains a high amount of glucocorticoid receptors [51], which we also found in our expression data. Further evidence for a role of the CB in hormonal regulation of IOP came from Southren and co-workers, who showed translocation of glucocorticoid receptors from the cytoplasm to the nucleus in the iris-ciliary body after topical administration of steroids in rabbits [52]. This translocation is the basic mechanism for steroid hormone to regulate differential gene expression in the nucleus and possible changed cell activity as a consequence. Interestingly, oral corticosteroid therapy seem to increase the rate of aqueous humor production in humans [53] and the aqueous is slightly more alkalic after long-term topical steroid administration [54]. In line with these findings, we suggest that steroids might also influence the human CB in such a way that not the production, but the composition of the aqueous humor alters. This may ultimately affect the molecular interactions with the TM and increase the outflow resistance.

Besides the endocrinological signaling pathways, we also found several pathways of metabolism and disease. These molecular mechanisms included metabolism of glucose, lipid and vitamins and the disease diabetes mellitus. The pathways and involved genes concerning glucose metabolism are interesting in light of the function of the aqueous humor. The AH is responsible for the nourishment of avascular tissue, like the lens and cornea. Previously, Chan and co-workers found leads for active glucose transport in the bovine CB [55]. Our expression data also point to that functionality and might give more insight in these mechanisms of glucose concentration in the AH.

#### Immunological properties

We found that many genes of the immune system were highly expressed in the NPE and PE. Apparently, the NPE and PE are involved in different types of innate and adaptive immune responses, including (chronic) auto-immune reactions and viral infections. Some major groups of entries which were highly expressed included chemokines, complement components, CD molecules, HLA subtypes, heat shock proteins, interleukins and integrin subtypes (see Table S4, S8 and S10).

We confirmed the presence of the SERPING1 (C1 inhibitor) protein in the NPE and PE with immunostaining. Amongst others SERPING1 is involved in the down regulation of the classic complement cascade via regulation of components 2 and 4. Moreover, SERPING1 also decreases the activity of other proteins, including kallikrein, plasmin and coagulation factors XIa and XIIa. Next, we demonstrated that TLR3 protein is expressed in both NPE and PE. TLR3 is involved in pathogen recognition and activation of innate immunity.

Our finding that genes involved in viral infection pathways are highly expressed in the NPE and PE, is also of interest. The CB is not only involved in anterior uveitis [56], but it was also previously described as the ocular entry portal for the CMV virus [57]. Our current data may serve as entry point for further detailed studies about the mechanisms of viral transport over the CB, which are (so far) lacking in the literature.

### 2. Molecular Signature of NPE and PE

We constructed a molecular signature of the NPE and PE of the ciliary body by selecting genes from the *Significant different sub-dataset* with fold-changes more than 2.5 between NPE and PE. For the NPE, only three genes appeared to be cell layer specific (Table 1). One of these genes, PDE11A, codes for the cAMP and cGMP phosphodiesterase 11A protein, involved in signal transduction. Mutations in PDE11A can cause Cushing disease and adrenocortical hyperplasia. Next, CDR1 is an intronless gene coding for cerebellar degeneration-related antigen 1. Against this protein autoantibodies were found in some patients with paraneoplastic cerebellar degeneration, which occurs in association with several forms of cancer. This protein contains tandem hexapeptide repeats and is expressed in brain, predominantly in normal neuroectodermal tissues and in certain malignant tumors (LocusLink, Swiss-Prot and NCI). Expression of these proteins in the CB has neither reported nor studied yet.

For the PE we found 143 specific genes (Table 2). Besides many genes coding for clones and yet unknown proteins, or proteins with broad and more basic cellular functions, we found genes coding for proteins involved in pigmentation, cytoskeleton and extracellular matrix assembly, (neuro)endocrine and metabolic process and developmental pathways. The fact that we found several genes and pathways involved in pigmentation in the PE (DCT, GPNMB, MLANA, SERPINF1, TYR and TYRP) versus the NPE served as an internal control that our (other) data are meaningful. Indeed, some of these genes were already found to be expressed in the CB (GPNMB and TYR [58], [59]). For the other genes, specific studies of their localization and function in the ciliary body are still lacking.

Next, we found several genes involved in cytoskeleton and extracellular matrix (ECM) assembly. Apparently, the PE expresses these ECM genes significantly higher than the NPE. A possible explanation is that the PE lines the CB stroma, whereas the more internally located NPE does not. Most likely, the PE contributes to the formation or turnover of the CB stroma. Part of our data confirms the studies of Ortego and coworkers [60] and Mandel [61], who found high and specific expression of CTSK and CTRP5 in the PE. Of interest, mutations in CTRP5 are associated with early-onset anterior zonules (LAZ) and late-onset retinal degeneration (L-ORD) and some of these patients also developed secondary glaucoma at a late stage of their disease [62]. The PE specific expressed genes with (neuro)endorcrine functionalities provide possibly more insight in local cell-specific regulation of the AH production by the CB. For example, we confirmed the expression of AGT and CPA6 in the PE. Both entries are involved in blood pressure regulation, which, in turn, is associated with IOP pressure. AGT codes for angiotensinogen in the CB [63], [64] and CPA6 may convert inactive angiotensin I into the biologically active angiotensin II. Consequently, these genes may play a role in IOP regulations/sensory mechanisms. According to our current data, the PE, located adjacent to the fenestrated blood capillaries of the stroma, may play an important role in these processes.

We also found four genes involved in lipid transport and metabolism specific for the PE, namely ABCA1, ABCC1, LIPI and LPL. Of these, ABCA1 is already known to be expressed in the RPE [65] and LPL is, amongst others, found in the CB [66]. These molecules might play an important role in uptake of fatty acids by CB from the blood.

Finally, we found seventeen genes specifically expressed in the PE involved in embryonic and developmental process. More specifically, we identified five retinal progenitor markers to be specifically expressed in the PE compared to the NPE, namely HES [67], MSX1, MSX2 [68], TNFRSF19 [69] and TLL1 [70]. This finding is of interest, since the possible stem cell properties of the PE are still under debate (discussed above).

### 3. (N)PE Expression of Genes Previously Implicated in Glaucoma

We found 35 entries in our *Highly expressed NPE and PE* and *Significant different expressed sub-datasets* that were previously implicated in the genetics or patho-physiology of glaucoma. It is striking that so many genes previously implicated in glaucoma, are (also) highly expressed in healthy human ciliary body. Of interest are the seven genes that were previously been genetically associated to POAG (included NTG), namely APOE, CAV1, CDKN2A, EDNRA, MYOC, OPTC and TMCO1. High expression of these genes in the CB might implicate that the CB is actively involved in the pathogenesis of POAG, possible by influencing the AH composition. Indeed, proteomic analysis of the AH of POAG patients showed changed composition. Glaucomatous AH contained not only an increased amount of proteins, but also possible harmful substances like PGDS and CASP14 (possible inducting apoptosis of TM cells) [71]. This changed composition might affect the TM sieve, by direct molecular interaction, or by clotting of protein aggregates. These changes may subsequently cause decreased outflow facilities for the AH and increase IOP as a consequence.

Also of interest is the expression of the *TTR* gene in the (N)PE. Additionally, two previous proteomic analysis studies provided evidence that the TTR levels were increased in the AH of POAG patients compared to controls [71], [72]. The protein encoded by this *TTR* is involved in thyroxine transport. Defects in this gene cause amyloidosis transthyretin-related disorder (AMYL-TTR). This disease is characterized by depositions of polymerized mutated TTR in peripheral nerves, gastrointestinal tract, heart, and ocular tissues. Also, the disorder is associated with secondary glaucoma [73], [74]. Normal treatment of this disease is the transplantation of liver, since the pathogenic protein is predominantly produced by the liver. Interestingly, in contrast to other organs where the mutated TTR deposits disappear after liver transplantation, the risk of glaucoma does not decrease after liver transplantation in patients [75], [76]. This leads us to hypothesize that the local and not systemic regulation of TTR expression by the ciliary body could have pathological consequences.

Finally, of further interest is that our functional analysis of the CB transcriptome suggested NPE and PE involvement in a variety of metabolic process, including lipid and vitamin metabolism. Some of these processes were also associated with POAG. For example, Ramdas and coworkers showed that high intakes of vitamin A and B1 were associated with a decreased risk of POAG [77]. In yet another, more controversial study, statin, a lipid lowering drug, appeared to have a protective effect for POAG [78]. All these effects were IOP independent. These factors however might influence the CB and the AH composition, with possible harmful effects for the TM or the retinal ganglion cells over time. Further studies are needed to determine the exact role of the molecular mechanisms of the CB in increased IOP and glaucoma.

### Conclusion

Our study provides the first detailed description of the gene expression profile of the neuro-epithelia of the CB, based on a microarray study of the NPE and PE. We performed a detailed molecular and functional analysis of the NPE and PE, guided by a number of CB functionalities previously described (embryological properties, neural nature, endocrine and metabolic signaling, and immune privileges). Although the NPE and PE showed strong similarities, we were also able to deduce molecular signatures of the NPE and PE. These showed that especially the PE expressed cell-specific genes, involved in pigmentation, cytoskeleton, endocrine and metabolic pathways and neural retinal developmental properties. Finally, we found high expression in the CB of many genes previously attributed to POAG. This finding indicates a possible more prominent role of the CB in the pathogenesis of POAG than was previously thought.

## Supporting Information

Figure S1 **Separate laser dissection microscopy of the NPE and PE.** Separate cut out of the non-pigmented (NPE) and pigmented epithelium (PE) from the ciliary body by laser dissection. Frozen sections of 20 µm were stained with cresyl violet to distinguish the NPE (a). Next, the NPE was cut out with laser and collected in a tube. The green dotted line is the selection which the laser will cut. The number 62 refers to the 62th selection that was made (b). Subsequently, the PE was cut out (c). The stroma remains after laser dissection of NPE and PE (d).
(TIF)

Figure S2 **Huntington’s disease signaling pathway identified by the Ingenuity software.** This is one of the canonical pathways that contains statistically significantly more genes than expected by chance in the group of genes that are highly expressed in the non-pigmented epithelium (NPE) of the ciliary body. Gray fields indicate their presence among the highest expressed genes of the NPE; uncolored genes are added by the Ingenuity software to form the pathway. Solid lines between molecules indicate direct physical relationships between molecules (such as regulating and interacting protein domains); dotted lines indicate indirect functional relationships (such as co-regulation of expression of both genes in cell lines). Abbreviations of gene names are according to standard abbreviations used in Genbank.
(JPG)

Figure S3 **Androgen signaling pathway identified by the Ingenuity software.** This is one of the canonical pathways that contains statistically significantly more genes than expected by chance in the group of genes that are highly expressed in the non-pigmented epithelium (NPE) of the ciliary body. For explanation of symbols on the diagrams see legend Figure S2.
(JPG)

Figure S4 **Glucocorticoid receptor signaling pathway identified by the Ingenuity software.** This is one of the canonical pathways that contains statistically significantly more genes than expected by chance in the group of genes that are highly expressed in the non-pigmented epithelium (NPE) of the ciliary body. For explanation of symbols on the diagrams see legend Figure S2.
(JPG)

Figure S5 **Ephrin receptor signaling pathway identified by the Ingenuity software.** This is one of the canonical pathways that contains statistically significantly more genes than expected by chance in the group of genes that are highly expressed in the non-pigmented epithelium (NPE) of the ciliary body. For explanation of symbols on the diagrams see legend Figure S2.
(JPG)

Figure S6 **Glycolysis/gluconeogenesis pathway identified by the Ingenuity software.** This is one of the canonical pathways that contains statistically significantly more genes than expected by chance in the group of genes that are highly expressed in the non-pigmented epithelium (NPE) of the ciliary body. For explanation of symbols on the diagrams see legend Figure S2.
(JPG)

Figure S7 **Antigen presentation pathway identified by the Ingenuity software.** This is one of the canonical pathways that contains statistically significantly more genes than expected by chance in the group of genes that are highly expressed in the non-pigmented epithelium (NPE) of the ciliary body. For explanation of symbols on the diagrams see legend Figure S2.
(JPG)

Figure S8 **IGF-1 signaling pathway identified by the Ingenuity software.** This is one of the canonical pathways that contains statistically significantly more genes than expected by chance in the group of genes that are highly expressed in the non-pigmented epithelium (NPE) of the ciliary body. For explanation of symbols on the diagrams see legend Figure S2.
(JPG)

Figure S9 **Oxidative phosphorylation pathway identified by the Ingenuity software.** This is one of the canonical pathways that contains statistically significantly more genes than expected by chance in the group of genes that are highly expressed in the non-pigmented epithelium (NPE) of the ciliary body. For explanation of symbols on the diagrams see legend Figure S2.
(JPG)

Figure S10 **Hypoxia signaling in the cardiovascular system pathway identified by the Ingenuity software.** This is one of the canonical pathways that contains statistically significantly more genes than expected by chance in the group of genes that are highly expressed in the non-pigmented epithelium (NPE) of the ciliary body. For explanation of symbols on the diagrams see legend Figure S2.
(JPG)

Figure S11 **Molecular network generated by the Ingenuity software from the highest expressed genes of the NPE.** Molecular network generated from our microarray data of the highest expressed genes of the non-pigmented epithelium (NPE). Grey symbols represent genes that are highly expressed in the NPE of the ciliary body. Transparent entries are molecules from the knowledge database, inserted to connect all relevant molecules in a single network. Solid lines between molecules indicate direct physical relationships between molecules (such as regulating and interacting protein domains); dotted lines indicate indirect functional relationships (such as co-regulation of expression of both genes in cell lines). Abbreviations of gene names are according to standard abbreviations used in Genbank. The main functionalities given by Ingenuity for this molecular network are ‘Cellular development, cellular growth and proliferation, renal proliferation’. Of interest is that this network is mainly build up around the gene FOS. FOS proteins have been implicated as regulators of cell proliferation, differentiation and transformation during development
(JPG)

Figure S12 **Molecular network generated by the Ingenuity software from the highest expressed genes of the NPE.** Molecular network generated from our microarray data of the highest expressed genes of the non-pigmented epithelium (NPE). For explanation of symbols on the diagrams see legend Figure S11. The main functionalities given by Ingenuity for this molecular network are ‘Cancer, reproductive system disease, carbohydrate metabolism’. Highlights in this network are the molecules MEIS2, MAML1 and FOXP1 that are all involved in developmental processes. Moreover, this network contained the gene OPTC, which have been previously attributed to glaucoma.
(JPG)

Figure S13 **Molecular network generated by the Ingenuity software from the highest expressed genes of the NPE.** Molecular network generated from our microarray data of the highest expressed genes of the non-pigmented epithelium (NPE). For explanation of symbols on the diagrams see legend Figure S11. The main functionalities given by Ingenuity for this molecular network are ‘Organismal development, tissue development, cellular development’. Highlights in this network are the molecules FGFR1, FGFR2, SFRP1 and CCNH that are all involved in developmental processes.
(JPG)

Figure S14 **Molecular network generated by the Ingenuity software from the highest expressed genes of the NPE.** Molecular network generated from our microarray data of the highest expressed genes of the non-pigmented epithelium (NPE). For explanation of symbols on the diagrams see legend Figure S11. The main functionalities given by Ingenuity for this molecular network are ‘Gene expression, infectious disease, embryonic development’. Highlights in this network are the molecules AES and SIX6 that are involved in developmental processes. Moreover, this network contained the gene TNFRSF14, which protein is involved in viral entry mechanisms.
(JPG)

Figure S15 **Molecular network generated by the Ingenuity software from the highest expressed genes of the NPE.** Molecular network generated from our microarray data of the highest expressed genes of the non-pigmented epithelium (NPE). For explanation of symbols on the diagrams see legend Figure S11. The main functionalities given by Ingenuity for this molecular network are ‘Cell morphology, cellular function and maintenance, connective tissue development and function’. This network contained the gene MAP1LC3B, which protein product is involved in neurogenesis. Also three genes coding for GABA receptor associated proteins were present in this network, mediating inhibition of neurotransmission. This network is an example of the neural background we found in the NPE.
(JPG)

Figure S16 **Molecular network generated by the Ingenuity software from the highest expressed genes of the NPE.** Molecular network generated from our microarray data of the highest expressed genes of the non-pigmented epithelium (NPE). For explanation of symbols on the diagrams see legend Figure S11. The main functionalities given by Ingenuity for this molecular network are ‘Post-translational modification, connective tissue development and function, embryonic development’. In this network, we found the gene UCHL1, which is a neuron specific gene and previously associated with Parkinson’s disease.
(JPG)

Figure S17 **Molecular network generated by the Ingenuity software from the highest expressed genes of the NPE.** Molecular network generated from our microarray data of the highest expressed genes of the non-pigmented epithelium (NPE). For explanation of symbols on the diagrams see legend Figure S11. The main functionalities given by Ingenuity for this molecular network are ‘Hereditary disorder, metabolic disease, cardiovascular disease’. This network contained several genes that have been associated with neurological diseases, for example Huntington’s disease (NDUFA7, NDUFC1, NDUFB2, NDUFB3, NDUFA8), atrophy of optic nerve (NDUFS4) and Leigh syndrome (NDUFS7, NDUFA2, NDUFS). This network is an example of the neural background we found in the NPE.
(JPG)

Figure S18 **Molecular network generated by the Ingenuity software from the highest expressed genes of the NPE.** Molecular network generated from our microarray data of the highest expressed genes of the non-pigmented epithelium (NPE). For explanation of symbols on the diagrams see legend Figure S11. The main functionalities given by Ingenuity for this molecular network are ‘Molecular transport, neurological disease, connective tissue disorders’. This network contained two genes previously associated with neurological diseases, namely ZFYVE27 (hereditary spastic paraplegia) and SPG21 (neurodegeneration of photoreceptors). Also, we found genes involved in nervous system development, for example ATP1B1 (involved in aggregation of neurons), TRIM2 (neuroprotection of cortical neurons) and VAPA, SGK1 and ZFYVE27 (involved in morphogenesis of neuritis). This network is an example of the neural background we found in the NPE.
(JPG)

Figure S19 **Molecular network generated by the Ingenuity software from the highest expressed genes of the NPE.** Molecular network generated from our microarray data of the highest expressed genes of the non-pigmented epithelium (NPE). For explanation of symbols on the diagrams see legend Figure S11. The main functionalities given by Ingenuity for this molecular network are ‘Lipid metabolism, small molecule biochemistry, carbohydrate metabolism’. In this network we found genes involved in metabolic pathways, like LPL and CYP1B1 (lipid metabolism), IDH1 (energy production), GSTM3, GSTP1, GSTO1 and MT2A (detoxification and drug metabolism), SLC23A2 (transport of vitamin C) and SLC39A8 (zinc transport). This network is an example of the metabolic functionalities that we found in the NPE.
(JPG)

Figure S20 **Molecular network generated by the Ingenuity software from the highest expressed genes of the NPE.** Molecular network generated from our microarray data of the highest expressed genes of the non-pigmented epithelium (NPE). For explanation of symbols on the diagrams see legend Figure S11. The main functionalities given by Ingenuity for this molecular network are ‘Embryonic development, tissue morphology, organ development’. This network contained several subtypes of aldehyde dehydrogenase (ALDH1A1, ALDH1A3, ALDH4A1, ALDH6A1 and ALDH9A1) that are involved in alcohol, retinol and lipid metabolism. Therefore, this network is an example of the metabolic functionalities that we found in the NPE.
(JPG)

Figure S21 **Molecular network generated by the Ingenuity software from the highest expressed genes of the NPE.** Molecular network generated from our microarray data of the highest expressed genes of the non-pigmented epithelium (NPE). For explanation of symbols on the diagrams see legend Figure S11. The main functionalities given by Ingenuity for this molecular network are ‘Cell death, drug metabolism, small molecule biochemistry’. This network contained several genes which protein products are involved in immunosuppressive process and HIV infection, namely PPIA, PPIB, FKBP3 and FKBP9. Therefore, this network is an example for the immunological properties of the NPE.
(JPG)

Figure S22 **Molecular network generated by the Ingenuity software from the highest expressed genes of the NPE.** Molecular network generated from our microarray data of the highest expressed genes of the non-pigmented epithelium (NPE). For explanation of symbols on the diagrams see legend Figure S11. The main functionalities given by Ingenuity for this molecular network are ‘Developmental disorder, hereditary disorder, neurological disease’. This network contained the gene TMCO1, which have been previously associated with POAG.
(JPG)

Figure S23 **Wnt/β-catenin signaling pathway identified by the Ingenuity software.** This is one of the canonical pathways that contains statistically significantly more genes than expected by chance in the group of genes that differ statistically significantly between non-pigmented (NPE) and pigmented epithelium (PE). Red fields indicate genes statistically significantly higher expressed in PE compared to NPE whereas green fields indicate genes statistically significantly higher expressed in NPE compared to PE. Fields with both green and red color represent gene groups, of which some genes are significantly higher expressed in NPE (green) and other in PE (red). Uncolored genes are added by the software to form the pathway. Solid lines between molecules indicate direct physical relationships between molecules (such as regulating and interacting protein domains); dotted lines indicate indirect functional relationships (such as co-regulation of expression of both genes in cell lines). Abbreviations of gene names are according to standard abbreviations used in Genbank.
(JPG)

Figure S24 **Glioblastoma multiforme signaling pathway identified by the Ingenuity software.** This is one of the canonical pathways that contains statistically significantly more genes than expected by chance in the group of genes that differ statistically significantly between non-pigmented (NPE) and pigmented epithelium (PE). For explanation of symbols on the diagrams see legend Figure S23.
(JPG)

Figure S25 **Neuropathic pain signaling in dorsal horn neurons pathway identified by the Ingenuity software.** This is one of the canonical pathways that contains statistically significantly more genes than expected by chance in the group of genes that differ statistically significantly between non-pigmented (NPE) and pigmented epithelium (PE). For explanation of symbols on the diagrams see legend Figure S23.
(JPG)

Figure S26 **Endothelin-1 signaling pathway identified by the Ingenuity software.** This is one of the canonical pathways that contains statistically significantly more genes than expected by chance in the group of genes that differ statistically significantly between non-pigmented (NPE) and pigmented epithelium (PE). For explanation of symbols on the diagrams see legend Figure S23.
(JPG)

Figure S27 **Cellular effects of Sildenafil (Viagra) pathway identified by the Ingenuity software.** This is one of the canonical pathways that contains statistically significantly more genes than expected by chance in the group of genes that differ statistically significantly between non-pigmented (NPE) and pigmented epithelium (PE). For explanation of symbols on the diagrams see legend Figure S23.
(JPG)

Figure S28 **Antigen presentation pathway identified by the Ingenuity software.** This is one of the canonical pathways that contains statistically significantly more genes than expected by chance in the group of genes that differ statistically significantly between non-pigmented (NPE) and pigmented epithelium (PE). For explanation of symbols on the diagrams see legend Figure S23.
(JPG)

Figure S29 **Caveolar-mediated endocytosis signaling pathway identified by the Ingenuity software.** This is one of the canonical pathways that contains statistically significantly more genes than expected by chance in the group of genes that differ statistically significantly between non-pigmented (NPE) and pigmented epithelium (PE). For explanation of symbols on the diagrams see legend Figure S23.
(JPG)

Figure S30 **Gap junction signaling pathway identified by the Ingenuity software.** This is one of the canonical pathways that contains statistically significantly more genes than expected by chance in the group of genes that differ statistically significantly between non-pigmented (NPE) and pigmented epithelium (PE). For explanation of symbols on the diagrams see legend Figure S23.
(JPG)

Figure S31 **Molecular network generated by Ingenuity software from the statistically significantly different expressed genes.** Molecular network generated from our microarray *Significantly Different Expressed sub-dataset.* Red fields indicate genes statistically significantly higher expressed in pigmented epithelium (PE) compared to non-pigmented epithelium (NPE) whereas green fields indicate genes statistically significantly higher expressed in NPE compared to PE. Transparent entries are molecules from the knowledge database, inserted to connect all relevant molecules in a single network. Solid lines between molecules indicate direct physical relationships between molecules (such as regulating and interacting protein domains); dotted lines indicate indirect functional relationships (such as co-regulation of expression of both genes in cell lines). Abbreviations of gene names are according to standard abbreviations used in Genbank. The main functionalities given by Ingenuity for this molecular network are ‘Connective tissue development and function, embryonic development, organ development’. This network contained several genes involved in embryonic development, namely genes SOX8, SOX11, MSX1, FRZB and WNT2B.
(JPG)

Figure S32 **Molecular network generated by Ingenuity software from the statistically significantly different expressed genes.** Molecular network generated from our microarray *Significantly Different Expressed sub-dataset.* For explanation of symbols on the diagrams see legend Figure S31. The main functionalities given by Ingenuity for this molecular network are ‘Carbohydrate metabolism, molecular transport, small molecule biochemistry’. Of interest, this network contained the gene SIX6, whose protein product is involved in eye development.
(JPG)

Figure S33 **Molecular network generated by Ingenuity software from the statistically significantly different expressed genes.** Molecular network generated from our microarray *Significantly Different Expressed sub-dataset.* For explanation of symbols on the diagrams see legend Figure S31. The main functionalities given by Ingenuity for this molecular network are ‘Amino acid metabolism, molecular transport, small molecule biochemistry’. Of interest, this network contained the gene CLRN1, whose protein product is involved in the development of retina.
(JPG)

Figure S34 **Molecular network generated by Ingenuity software from the statistically significantly different expressed genes.** Molecular network generated from our microarray *Significantly Different Expressed sub-dataset.* For explanation of symbols on the diagrams see legend Figure S31. The main functionalities given by Ingenuity for this molecular network are ‘Cellular assembly and organization, cellular function and maintenance, cell death’. In this network we found centrally the gene DISC1. The protein of this gene is involved in neurite outgrowth and cortical development. Also, this gene is associated with schizophrenia. The gene DST is involved in transport of axons and coalignment of neurofilaments and the gene OLFM1 is neural tissue specific gene.
(JPG)

Figure S35 **Molecular network generated by Ingenuity software from the statistically significantly different expressed genes.** Molecular network generated from our microarray *Significantly Different Expressed sub-dataset.* For explanation of symbols on the diagrams see legend Figure S31. The main functionalities given by Ingenuity for this molecular network are ‘Connective tissue development and function, embryonic development, nervous system development and function’. In this network we found two genes that have been associated with neurodegeneration, namely PTGS2 and PSEN1. The protein products of PSEN1 and ABCA1 are involved in amyloid-beta metabolism. Moreover, the genes NOS1 and PTGS2 have been associated with glaucoma.
(JPG)

Figure S36 **Molecular network generated by Ingenuity software from the statistically significantly different expressed genes.** Molecular network generated from our microarray *Significantly Different Expressed sub-dataset.* For explanation of symbols on the diagrams see legend Figure S31. The main functionalities given by Ingenuity for this molecular network are ‘Cell morphology, cellular assembly and organization, cellular development’. This network contained the genes GRIN2C and GRIN3A, both coding for NMDA glutamate receptors that are important for neural signaling, and the genes EPH1, EPH2 and FOXP2, that are involved in morphogenesis of the eye.
(JPG)

Figure S37 **Molecular network generated by Ingenuity software from the statistically significantly different expressed genes.** Molecular network generated from our microarray *Significantly Different Expressed sub-dataset.* For explanation of symbols on the diagrams see legend Figure S31. The main functionalities given by Ingenuity for this molecular network are ‘Lipid metabolism, molecular transport, small molecule biochemistry’. This network contained several molecules involved in lipid metabolism, namely APOE, LDL, OSBP and ELOVL3. Moreover, we found a gene in this network involved in drug metabolism (CES1) and glucose transport (SLC2A2).
(JPG)

Figure S38 **Molecular network generated by Ingenuity software from the statistically significantly different expressed genes.** Molecular network generated from our microarray *Significantly Different Expressed sub-dataset.* For explanation of symbols on the diagrams see legend Figure S31. The main functionalities given by Ingenuity for this molecular network are ‘Carbohydrate metabolism, molecular transport, small molecule biochemistry’. This network was constructed with many G-coupled receptors that differed statistically significant between NPE and PE. G-coupled receptors are activated by extracellular stimuli, like neurotransmitters, hormones and light, and activate intracellular process. Apparently, some of these receptors differ between NPE and PE, for example GRM1 and GRM8 (glutamate receptor), ADRA2A (adrenergic alpha receptor), HTR2C (serotonin receptor), GPER (estrogen receptor) and EDNRB (endothelin B receptor).
(JPG)

Figure S39 **Molecular network generated by Ingenuity software from the statistically significantly different expressed genes.** Molecular network generated from our microarray *Significantly Different Expressed sub-dataset.* For explanation of symbols on the diagrams see legend Figure S31. The main functionalities given by Ingenuity for this molecular network are ‘Cell-to-cell signaling and interaction, inflammatory response, carbohydrate metabolism’. In this network we found several genes whose protein products are involved in immunological process, including IFNG, HAVCR2 and KLRB1 (activation of macrophages and natural killer cells), CD48 and CRTAM (T-cell mediated immune response), HCP5 and HLA-DQ (HLA complex subtypes).
(JPG)

Figure S40 **Molecular network generated by Ingenuity software from the statistically significantly different expressed genes.** Molecular network generated from our microarray *Significantly Different Expressed sub-dataset.* For explanation of symbols on the diagrams see legend Figure S31. The main functionalities given by Ingenuity for this molecular network are ‘Cell-to-cell signaling and interaction, inflammatory response, cellular function and maintenance’. In this network we identified the genes STAT4 whose protein product mediates responses to IL12 lymphocytes and regulate the differentiation of T-helper cells. Moreover, this network contained the gene TLR3 (fundamental role in pathogen recognition and activation of innate immunity), IGSF21 (an immunoglobulin) and CD2 and CD69 (activation of T-lymphocytes).
(JPG)

Figure S41 **Molecular network generated by Ingenuity software from the statistically significantly different expressed genes.** Molecular network generated from our microarray *Significantly Different Expressed sub-dataset.* For explanation of symbols on the diagrams see legend Figure S31. The main functionalities given by Ingenuity for this molecular network are ‘Connective tissue disorders, cellular movement, skeletal and muscular system development and function’. This network contained genes coding for cadherin (CDH6, CDH11), catenin (CTNNA2, CTNND2), collagen (COL13A1, COL15A1) and extracellular matrix protein (ECM1).
(JPG)

Figure S42 **Confirmation of microarray results by s-QPCR.** A: significantly different expressed genes with higher expression in NPE. Beta actin, a household gene, was used to normalize gene expression in the non-pigmented (NPE) and pigmented epithelium (PE) of the ciliary body. Seven genes that differed statistically significant between NPE and PE, with higher expression in NPE were confirmed by s-QPCR and outlined here. The grey bars indicate NPE expression and the black bars PE expression. Standard deviation is calculated. Three genes (CCND1, FAM43B, NR0B1) did not give any PCR products, even after repetitive attempts. B. Confirmation of microarray results by s-QPCR: significantly different expressed genes with higher expression in PE. Beta actin, a household gene, was used to normalize gene expression in the non-pigmented (NPE) and pigmented epithelium (PE) of the ciliary body. Eight genes that differed statistically significant between NPE and PE, with higher expression in PE were confirmed by s-QPCR and outlined here. The grey bars indicate NPE expression and the black bars PE expression. Standard deviation is calculated. Two genes (MAPK4, THC2675163) did not give any PCR products, even after repetitive attempts.
(TIF)

Table S1 **Donor eye data.**
(DOC)

Table S2 **Previously studied immune-localization of proteins in the ciliary body; A literature study.**
(DOC)

Table S3 (DOC)
**Overview of the proteins and their protocols for immunohistochemistry.**

Table S4 **10%H NPE.**
(XLS)

Table S5 **Biological functions assigned by Ingenuity to the NPE and PE.**
(DOCX)

Table S6 **Canonical pathways assigned by Ingenuity to the NPE and PE.**
(DOCX)

Table S7 **Molecular networks.**
(XLS)

Table S8 **10%H PE.**
(XLS)

Table S9 **Molecular networks.**
(XLS)

Table S10 **Significant different genes.**
(XLS)

Table S11 **Biological functions different between NPE and PE assigned by Ingenuity.**
(DOCX)

Table S12 **Canonical pathways different between NPE and PE assigned by Ingenuity.**
(DOC)

Table S13 **Functional molecular networks different between NPE and PE.**
(XLS)

## References

[1] Cunha-Vaz J (1979) The blood-ocular barriers. Surv Ophthalmol 23: 279–296. 380030 10.1016/0039-6257(79)90158-9

[2] Bhatia B, Singhal S, Lawrence JM, Khaw PT, Limb GA (2009) Distribution of Muller stem cells within the neural retina: Evidence for the existence of a ciliary margin-like zone in the adult human eye. Experimental Eye Research 89: 373–382. 19379736 10.1016/j.exer.2009.04.005

[3] Fischer AJ, Reh TA (2001) Transdifferentiation of Pigmented epithelial cells: A source of retinal stem cells? Developmental Neuroscience 23: 268–276. 11756742 10.1159/000048710

[4] Lord-Grignon J, Abdouh M, Bernier G (2006) Identification of genes expressed in retinal progenitor/stem cell colonies isolated from the ocular ciliary body of adult mice. Gene Expression Patterns 6: 992–999. 16765103 10.1016/j.modgep.2006.04.003

[5] Ahmad I, Tang L, Pham H (2000) Identification of neural progenitors in the adult mammalian eye. Biochem Biophys Res Commun 270: 517–521. 10753656 10.1006/bbrc.2000.2473

[6] Das AV, James J, Rahnenfuhrer J, Thoreson WB, Bhattacharya S, et al (2005) Retinal properties and potential of the adult mammalian ciliary epithelium stem cells. Vision Res 45: 1653–1666. 15792841 10.1016/j.visres.2004.12.017

[7] MacNeil A, Pearson RA, MacLaren RE, Smith AJ, Sowden JC, et al (2007) Comparative analysis of progenitor cells isolated from the iris, pars plana, and ciliary body of the adult porcine eye. Stem Cells 25: 2430–2438. 17600111 10.1634/stemcells.2007-0035

[8] Martinez-Navarrete GC, Angulo A, Martin-Nieto J, Cuenca N (2008) Gradual morphogenesis of retinal neurons in the peripheral retinal margin of adult monkeys and humans. J Comp Neurol 511: 557–580. 18839410 10.1002/cne.21860

[9] Inoue T, Coles BL, Dorval K, Bremner R, Bessho Y, et al (2010) Maximizing functional photoreceptor differentiation from adult human retinal stem cells. Stem Cells 28: 489–500. 20014120 10.1002/stem.279 PMC2933833

[10] Tropepe V, Coles BL, Chiasson BJ, Horsford DJ, Elia AJ, et al (2000) Retinal stem cells in the adult mammalian eye. Science 287: 2032–2036. 10720333 10.1126/science.287.5460.2032

[11] Xu H, Sta Iglesia DD, Kielczewski JL, Valenta DF, Pease ME, et al (2007) Characteristics of progenitor cells derived from adult ciliary body in mouse, rat, and human eyes. Invest Ophthalmol Vis Sci 48: 1674–1682. 17389499 10.1167/iovs.06-1034

[12] Cicero SA, Johnson D, Reyntjens S, Frase S, Connell S, et al (2009) Cells previously identified as retinal stem cells are pigmented ciliary epithelial cells. Proc Natl Acad Sci U S A 106: 6685–6690. 19346468 10.1073/pnas.0901596106 PMC2672506

[13] Gualdoni S, Baron M, Lakowski J, Decembrini S, Smith AJ, et al (2010) Adult Ciliary Epithelial Cells, Previously Identified as Retinal Stem Cells with Potential for Retinal Repair, Fail to Differentiate into New Rod Photoreceptors. Stem Cells 28: 1048–1059. 20506130 10.1002/stem.423

[14] Coca-Prados M, Escribano J (2007) New perspectives in aqueous humor secretion and in glaucoma: The ciliary body as a multifunctional neuroendocrine gland. Progress in Retinal and Eye Research 26: 239–262. 17321191 10.1016/j.preteyeres.2007.01.002

[15] Taylor AW (2009) Ocular immune privilege. Eye 23: 1885–1889. 19136922 10.1038/eye.2008.382 PMC4698145

[16] Sugita S (2009) Role of ocular pigment epithelial cells in immune privilege. Archivum Immunologiae et Therapiae Experimentalis 57: 263–268. 19568919 10.1007/s00005-009-0030-0

[17] Bishop PN, Takanosu M, Le GM, Mayne R (2002) The role of the posterior ciliary body in the biosynthesis of vitreous humour. Eye (Lond) 16: 454–460. 12101453 10.1038/sj.eye.6700199

[18] Rodrigues ML, Filho RB, Laicine EM, Haddad A (1998) Transferrin production by the ciliary body of rabbits: a biochemical and immunocytochemical study. Curr Eye Res 17: 694–699. 9678414

[19] Toates FM (1972) Accommodation function of the human eye. Physiological Reviews 52: 828–863. 4345068 10.1152/physrev.1972.52.4.828

[20] Ramdas WD, van Koolwijk LM, Cree AJ, Janssens AC, Amin N, et al (2011) Clinical implications of old and new genes for open-angle glaucoma. Ophthalmology 118: 2389–2397. 21872936 10.1016/j.ophtha.2011.05.040

[21] Goldhagen B, Proia AD, Epstein DL, Rao PV (2012) Elevated Levels of RhoA in the Optic Nerve Head of Human Eyes with Glaucoma. J Glaucoma. 10. 10.1097/IJG.0b013e318241b83c 22495072

[22] Naka M, Kanamori A, Negi A, Nakamura M (2010) Reduced expression of aquaporin-9 in rat optic nerve head and retina following elevated intraocular pressure. Invest Ophthalmol Vis Sci 51: 4618–4626. 20357197 10.1167/iovs.09-4712

[23] Schlotzer-Schrehardt U, Zenkel M, Kuchle M, Sakai LY, Naumann GOH (2001) Role of transforming growth factor-beta 1 and its latent form binding protein in pseudoexfoliation syndrome. Experimental Eye Research 73: 765–780. 11846508 10.1006/exer.2001.1084

[24] Booij JC, van Soest S, Swagemakers SMA, Essing AHW, Verkerk AJMH, et al.. (2009) Functional annotation of the human retinal pigment epithelium transcriptome. Bmc Genomics 10. 10.1186/1471-2164-10-164 PMC2679759 19379482

[25] Friedman DS, Wolfs RCW, O’Colmain B, Klein BE, Taylor HR, et al (2004) Prevalence of open-angle glaucoma among adults in the United States. Archives of Ophthalmology 122: 532–538. 15078671 10.1001/archopht.122.4.532 PMC2798086

[26] Booij JC, ten Brink JB, Swagemakers SM, Verkerk AJ, Essing AH, et al (2010) A new strategy to identify and annotate human RPE-specific gene expression. Plos One 5: e9341. 20479888 10.1371/journal.pone.0009341 PMC2866542

[27] van Soest SS, de Wit GM, Essing AH, ten Brink JB, Kamphuis W, et al (2007) Comparison of human retinal pigment epithelium gene expression in macula and periphery highlights potential topographic differences in Bruch’s membrane. Mol Vis 13: 1608–1617. 17893662

[28] Smyth GK, Speed T (2003) Normalization of cDNA microarray data. Methods 31: 265–273. 14597310 10.1016/s1046-2023(03)00155-5

[29] Smyth GK (2005) Limma: linear models for microarray data. In: R.Gentleman, V.Carey, S.Dudoit, R.Irizarry, W.Huber, editors. Bioinformatics and Computational Biology Solutions using R and Bioconductor. New York: Springer. 397–420.

[30] Smyth GK (2004) Linear models and empirical Bayes methods for assessing dierential expression in microarray experiments. Statistical Applications in Ge-netics and Molecular Biology 3: art3. 10.2202/1544-6115.1027 16646809

[31] Siegfried CJ, Shui YB, Holekamp NM, Bai F, Beebe DC (2010) Oxygen Distribution in the Human Eye: Relevance to the Etiology of Open-Angle Glaucoma after Vitrectomy. Investigative Ophthalmology & Visual Science 51: 5731–5738. 20720218 10.1167/iovs.10-5666 PMC3061509

[32] Zhang Y, Feng XH, Derynck R (1998) Smad3 and Smad4 cooperate with c-Jun/c-Fos to mediate TGF-beta-induced transcription. Nature 394: 909–913. 9732876 10.1038/29814

[33] Hodges A, Strand AD, Aragaki AK, Kuhn A, Sengstag T, et al (2006) Regional and cellular gene expression changes in human Huntington’s disease brain. Hum Mol Genet 15: 965–977. 16467349 10.1093/hmg/ddl013

[34] Weydt P, Pineda VV, Torrence AE, Libby RT, Satterfield TF, et al (2006) Thermoregulatory and metabolic defects in Huntington’s disease transgenic mice implicate PGC-1alpha in Huntington’s disease neurodegeneration. Cell Metab 4: 349–362. 17055784 10.1016/j.cmet.2006.10.004

[35] Genini S, Zangerl B, Slavik J, Acland GM, Beltran WA, et al (2010) Transcriptional profile analysis of RPGRORF15 frameshift mutation identifies novel genes associated with retinal degeneration. Invest Ophthalmol Vis Sci 51: 6038–6050. 20574030 10.1167/iovs.10-5443 PMC3061521

[36] Hoefs SJ, Dieteren CE, Distelmaier F, Janssen RJ, Epplen A, et al (2008) NDUFA2 complex I mutation leads to Leigh disease. Am J Hum Genet 82: 1306–1315. 18513682 10.1016/j.ajhg.2008.05.007 PMC2427319

[37] Lebon S, Rodriguez D, Bridoux D, Zerrad A, Rotig A, et al (2007) A novel mutation in the human complex I NDUFS7 subunit associated with Leigh syndrome. Mol Genet Metab 90: 379–382. 17275378 10.1016/j.ymgme.2006.12.007

[38] Quintana A, Kruse SE, Kapur RP, Sanz E, Palmiter RD (2010) Complex I deficiency due to loss of Ndufs4 in the brain results in progressive encephalopathy resembling Leigh syndrome. Proc Natl Acad Sci U S A 107: 10996–11001. 20534480 10.1073/pnas.1006214107 PMC2890717

[39] Martignoni M, Riano E, Rugarli EI (2008) The role of ZFYVE27/protrudin in hereditary spastic paraplegia. Am J Hum Genet 83: 127–128. 18606302 10.1016/j.ajhg.2008.05.014 PMC2443834

[40] Weber P, Bartsch U, Schachner M, Montag D (1998) Na,K-ATPase subunit beta1 knock-in prevents lethality of beta2 deficiency in mice. J Neurosci 18: 9192–9203. 9801359 10.1523/JNEUROSCI.18-22-09192.1998 PMC6792892

[41] Simpson MA, Cross H, Proukakis C, Pryde A, Hershberger R, et al (2003) Maspardin is mutated in mast syndrome, a complicated form of hereditary spastic paraplegia associated with dementia. Am J Hum Genet 73: 1147–1156. 14564668 10.1086/379522 PMC1180493

[42] Gerometta R, Alvarez LJ, Candia OA (2010) Effects of Sildenafil and Tadalafil on Intraocular Pressure in Sheep: Implications for Aqueous Humor Dynamics. Investigative Ophthalmology & Visual Science 51: 3139–3144. 20089876 10.1167/iovs.09-4862 PMC2891473

[43] Bayer AU, Keller ON, Ferrari F, Maag KP (2002) Association of glaucoma with neurodegenerative diseases with apoptotic cell death: Alzheimer’s disease and Parkinson’s disease. American Journal of Ophthalmology 133: 135–137. 11755850 10.1016/s0002-9394(01)01196-5

[44] Bayer AU, Ferrari F, Erb C (2002) High occurrence rate of glaucoma among patients with Alzheimer’s disease. European Neurology 47: 165–168. 11914555 10.1159/000047976

[45] Tamura H, Kawakami H, Kanamoto T, Kato T, Yokoyama T, et al (2006) High frequency of open-angle glaucoma in Japanese patients with Alzheimer’s disease. Journal of the Neurological Sciences 246: 79–83. 16564058 10.1016/j.jns.2006.02.009

[46] Yenice O, Onal S, Midi I, Ozcan E, Temel A, et al (2008) Visual field analysis in patients with Parkinson’s disease. Parkinsonism & Related Disorders 14: 193–198. 17888714 10.1016/j.parkreldis.2007.07.018

[47] Kass MA, Sears ML (1977) Hormonal-Regulation of Intraocular-Pressure. Survey of Ophthalmology 22: 153–176. 413203 10.1016/0039-6257(77)90053-4

[48] Bigger JF, Becker B, Palmberg PF (1972) Increased Cellular Sensitivity to Glucocorticoids in Primary Open-Angle Glaucoma. Investigative Ophthalmology 11: 832-&. 5071841

[49] Armaly MF (1963) Effect of corticosteroids on intraocular pressure and fluid dynamics I. The effect of dexamethasone in the normal eye. Arch Ophthalmol 70: 482–491. 14078870 10.1001/archopht.1963.00960050484010

[50] Miller D, Peczon JD, Whitworth CG (1965) Corticosteroids and functions in the anterior segment of the eye. Am J Ophthalmol 59: 31–34. 14259937 10.1016/0002-9394(65)95015-4

[51] McCarty GR, Schwartz B (1982) Increased concentration of glucocorticoid receptors in rabbit iris–ciliary body compared to rabbit liver. Invest Ophthalmol Vis Sci 23: 525–528. 6288612

[52] Southren AL, Gordon GG, Yeh HS, Dunn MW, Weinstein BI (1979) Nuclear translocation of the cytoplasmic glucocorticoid receptor in the iris–ciliary body of the rabbit. Invest Ophthalmol Vis Sci 18: 517–521. 437953

[53] Kimura R, Honda M (1982) Effect of orally administered hydrocortisone on the rate of aqueous flow in man. Acta Ophthalmol (Copenh) 60: 584–589. 7158320 10.1111/j.1755-3768.1982.tb00604.x

[54] Virno M, Schirru A, Pecori-Giraldi J, Pellegrino N (1974) Aqueous humor alkalosis and marked reduction in ocular ascorbic acid content following long-term topical cortisone (9alpha-fluoro-16alpha-methylprednisolone). Ann Ophthalmol 6: 983–992. 4429318

[55] Chan CY, Guggenheim JA, To CH (2007) Is active glucose transport present in bovine ciliary body epithelium? Am J Physiol Cell Physiol 292: C1087–C1093. 17020938 10.1152/ajpcell.00048.2006

[56] Peizeng Y, Qianli M, Xiangkun H, Hongyan Z, Li W, et al (2009) Longitudinal study of anterior segment inflammation by ultrasound biomicroscopy in patients with acute anterior uveitis. Acta Ophthalmol 87: 211–215. 18811638 10.1111/j.1755-3768.2008.01194.x

[57] Gao EK, Yu XH, Lin CP, Zhang H, Kaplan HJ (1995) Intraocular Viral Replication After Systemic Murine Cytomegalovirus-Infection Requires Immunosuppression. Investigative Ophthalmology & Visual Science 36: 2322–2327. 7558728

[58] Bachner D, Schroder D, Gross G (2002) mRNA expression of the murine glycoprotein (transmembrane) nmb (Gpnmb) gene is linked to the developing retinal pigment epithelium and iris. Brain Res Gene Expr Patterns 1: 159–165. 12638126 10.1016/s1567-133x(02)00012-1

[59] Hayasaka S, Nakazawa M, Ishiguro S, Mizuno K (1986) Presence of tyrosinase activity in human ciliary body. Jpn J Ophthalmol 30: 32–35. 3088305

[60] Ortego J, Escribano J, Coca-Prados M (1997) Gene expression of proteases and protease inhibitors in the human ciliary epithelium and ODM-2 cells. Exp Eye Res 65: 289–299. 9268597 10.1006/exer.1997.0333

[61] Mandal MN, Vasireddy V, Reddy GB, Wang X, Moroi SE, et al (2006) CTRP5 is a membrane-associated and secretory protein in the RPE and ciliary body and the S163R mutation of CTRP5 impairs its secretion. Invest Ophthalmol Vis Sci 47: 5505–5513. 17122142 10.1167/iovs.06-0312

[62] Ayyagari R, Mandal MN, Karoukis AJ, Chen L, McLaren NC, et al (2005) Late-onset macular degeneration and long anterior lens zonules result from a CTRP5 gene mutation. Invest Ophthalmol Vis Sci 46: 3363–3371. 16123441 10.1167/iovs.05-0159

[63] Murata M, Nakagawa M, Takahashi S (1997) Angiotensinogen mRNA is synthesized locally in rat ocular tissues. Ophthalmologica 211: 301–304. 9286806 10.1159/000310813

[64] Sramek SJ, Wallow IH, Tewksbury DA, Brandt CR, Poulsen GL (1992) An ocular renin-angiotensin system. Immunohistochemistry of angiotensinogen. Invest Ophthalmol Vis Sci 33: 1627–1632. 1559760

[65] Duncan KG, Hosseini K, Bailey KR, Yang H, Lowe RJ, et al (2009) Expression of reverse cholesterol transport proteins ATP-binding cassette A1 (ABCA1) and scavenger receptor BI (SR-BI) in the retina and retinal pigment epithelium. Br J Ophthalmol 93 1116–1120: bjo.2008. 10.1136/bjo.2008.144006 PMC3541028 19304587

[66] Casaroli-Marano RP, Peinado-Onsurbe J, Reina M, Staels B, Auwerx J, et al (1996) Lipoprotein lipase in highly vascularized structures of the eye. J Lipid Res 37: 1037–1044. 8725155

[67] Yanagi Y, Inoue Y, Kawase Y, Uchida S, Tamaki Y, et al (2006) Properties of growth and molecular profiles of rat progenitor cells from ciliary epithelium. Exp Eye Res 82: 471–478. 16198338 10.1016/j.exer.2005.08.005

[68] Liu H, Xu S, Wang Y, Mazerolle C, Thurig S, et al (2007) Ciliary margin transdifferentiation from neural retina is controlled by canonical Wnt signaling. Dev Biol 308: 54–67. 17574231 10.1016/j.ydbio.2007.04.052

[69] Ha A, Perez-Iratxeta C, Liu H, Mears AJ, Wallace VA (2012) Identification of Wnt/beta-catenin modulated genes in the developing retina. Mol Vis 18: 645–656. 22509096 PMC3324352

[70] Decembrini S, Cananzi M, Gualdoni S, Battersby A, Allen N, et al (2011) Comparative analysis of the retinal potential of embryonic stem cells and amniotic fluid-derived stem cells. Stem Cells Dev 20: 851–863. 20939691 10.1089/scd.2010.0291

[71] Duan XM, Xue P, Wang NL, Dong Z, Lu QJ, et al (2010) Proteomic analysis of aqueous humor from patients with primary open angle glaucoma. Molecular Vision 16: 2839–2846. 21203405 PMC3012650

[72] Grus FH, Joachim SC, Sandmann S, Thiel U, Bruns K, et al (2008) Transthyretin and complex protein pattern in aqueous humor of patients with primary open-angle glaucoma. Mol Vis 14: 1437–1445. 18682810 PMC2493027

[73] Nelson GA, Edward DP, Wilensky JT (1999) Ocular amyloidosis and secondary glaucoma. Ophthalmology 106: 1363–1366. 10406623 10.1016/S0161-6420(99)00726-5

[74] Kimura A, Ando E, Fukushima M, Koga T, Hirata A, et al (2003) Secondary glaucoma in patients with familial amyloidotic polyneuropathy. Arch Ophthalmol 121: 351–356. 12617705 10.1001/archopht.121.3.351

[75] Ando Y, Ando E, Tanaka Y, Yamashita T, Tashima K, et al (1996) De novo amyloid synthesis in ocular tissue in familial amyloidotic polyneuropathy after liver transplantation. Transplantation 62: 1037–1038. 8878404 10.1097/00007890-199610150-00028

[76] Sandgren O, Kjellgren D, Suhr OB (2008) Ocular manifestations in liver transplant recipients with familial amyloid polyneuropathy. Acta Ophthalmol 86: 520–524. 18435819 10.1111/j.1600-0420.2007.01098.x

[77] Ramdas WD, Wolfs RC, Kiefte-de Jong JC, Hofman A, de Jong PT, et al.. (2012) Nutrient intake and risk of open-angle glaucoma: the Rotterdam Study. Eur J Epidemiol. 10.1007/s10654-012-9672-z PMC3374099 22461101

[78] Marcus MW, Muskens RP, Ramdas WD, Wolfs RC, de Jong PT, et al (2012) Cholesterol-lowering drugs and incident open-angle glaucoma: a population-based cohort study. Plos One 7: e29724. 22238644 10.1371/journal.pone.0029724 PMC3251600

[79] Zenkel M, Poschl E, von der MK, Hofmann-Rummelt C, Naumann GO, et al (2005) Differential gene expression in pseudoexfoliation syndrome. Invest Ophthalmol Vis Sci 46: 3742–3752. 16186358 10.1167/iovs.05-0249

[80] Vickers JC, Craig JE, Stankovich J, McCormack GH, West AK, et al (2002) The apolipoprotein epsilon4 gene is associated with elevated risk of normal tension glaucoma. Mol Vis 8: 389–393. 12379839

[81] McKinnon SJ, Lehman DM, Kerrigan-Baumrind LA, Merges CA, Pease ME, et al (2002) Caspase activation and amyloid precursor protein cleavage in rat ocular hypertension. Invest Ophthalmol Vis Sci 43: 1077–1087. 11923249

[82] Boehm N, Wolters D, Thiel U, Lossbrand U, Wiegel N, et al (2012) New insights into autoantibody profiles from immune privileged sites in the eye: a glaucoma study. Brain Behav Immun 26: 96–102. 21843631 10.1016/j.bbi.2011.07.241

[83] Khor CC, Ramdas WD, Vithana EN, Cornes BK, Sim X, et al (2011) Genome-wide association studies in Asians confirm the involvement of ATOH7 and TGFBR3, and further identify CARD10 as a novel locus influencing optic disc area. Hum Mol Genet 20: 1864–1872. 21307088 10.1093/hmg/ddr060

[84] Thorleifsson G, Walters GB, Hewitt AW, Masson G, Helgason A, et al.. (2010) Common variants near CAV1 and CAV2 are associated with primary open-angle glaucoma. Nature Genetics 42: 906-+. 10.1038/ng.661 PMC3222888 20835238

[85] Burdon KP, Macgregor S, Hewitt AW, Sharma S, Chidlow G, et al (2011) Genome-wide association study identifies susceptibility loci for open angle glaucoma at TMCO1 and CDKN2B-AS1. Nat Genet 43: 574–578. 21532571 10.1038/ng.824

[86] Gibson J, Griffiths H, De SG, Cole M, Jacob A, et al (2012) Genome-wide association study of primary open angle glaucoma risk and quantitative traits. Mol Vis 18: 1083–1092. 22605921 PMC3351427

[87] Comes N, Borras T (2009) Individual molecular response to elevated intraocular pressure in perfused postmortem human eyes. Physiological Genomics 38: 205–225. 19401404 10.1152/physiolgenomics.90261.2008 PMC2712225

[88] Browne JG, Ho SL, Kane R, Oliver N, Clark AF, et al.. (2011) Connective Tissue Growth Factor is increased in Pseudoexfoliation Glaucoma. Invest Ophthalmol Vis Sci. iovs.10–7091 10.1167/iovs.10-5209 21330667

[89] Martin SN, Sutherland J, Levin AV, Klose R, Priston M, et al (2000) Molecular characterisation of congenital glaucoma in a consanguineous Canadian community: a step towards preventing glaucoma related blindness. J Med Genet 37: 422–427. 10851252 10.1136/jmg.37.6.422 PMC1734606

[90] Ishikawa K, Funayama T, Ohtake Y, Kimura I, Ideta H, et al (2005) Association between glaucoma and gene polymorphism of endothelin type A receptor. Mol Vis 11: 431–437. 15988412

[91] Kim S, Kim J, Kim D, Ko H, Kim S, et al (2006) Investigations on the association between normal tension glaucoma and single nucleotide polymorphisms of the endothelin-1 and endothelin receptor genes. Molecular Vision 12: 1016–1021. 16971893

[92] Musa FU, Ratajczak P, Sahu J, Pentlicky S, Fryer A, et al (2009) Ocular manifestations in oculodentodigital dysplasia resulting from a heterozygous missense mutation (L113P) in GJA1 (connexin 43). Eye (Lond) 23: 549–555. 18425059 10.1038/eye.2008.77

[93] Vasconcellos JP, Melo MB, Schimiti RB, Bressanim NC, Costa FF, et al (2005) A novel mutation in the GJA1 gene in a family with oculodentodigital dysplasia. Arch Ophthalmol 123: 1422–1426. 16219735 10.1001/archopht.123.10.1422

[94] Anderson MG, Smith RS, Hawes NL, Zabaleta A, Chang B, et al (2002) Mutations in genes encoding melanosomal proteins cause pigmentary glaucoma in DBA/2J mice. Nat Genet 30: 81–85. 11743578 10.1038/ng794

[95] Kalesnykas G, Niittykoski M, Rantala J, Miettinen R, Salminen A, et al (2007) The expression of heat shock protein 27 in retinal ganglion and glial cells in a rat glaucoma model. Neuroscience 150: 692–704. 17993247 10.1016/j.neuroscience.2007.09.078

[96] Ali M, McKibbin M, Booth A, Parry DA, Jain P, et al (2009) Null mutations in LTBP2 cause primary congenital glaucoma. Am J Hum Genet 84: 664–671. 19361779 10.1016/j.ajhg.2009.03.017 PMC2680998

[97] Xue W, Comes N, Borras T (2007) Presence of an established calcification marker in trabecular meshwork tissue of glaucoma donors. Investigative Ophthalmology & Visual Science 48: 3184–3194. 17591888 10.1167/iovs.06-1403 PMC1994153

[98] Angius A, De GE, Loi A, Fossarello M, Sole G, et al (1998) A novel mutation in the GLC1A gene causes juvenile open-angle glaucoma in 4 families from the Italian region of Puglia. Arch Ophthalmol 116: 793–797. 9639450 10.1001/archopht.116.6.793

[99] Shimizu S, Lichter PR, Johnson AT, Zhou Z, Higashi M, et al (2000) Age-dependent prevalence of mutations at the GLC1A locus in primary open-angle glaucoma. Am J Ophthalmol 130: 165–177. 11004290 10.1016/s0002-9394(00)00536-5

[100] Vasconcellos JP, Melo MB, Costa VP, Tsukumo DM, Basseres DS, et al (2000) Novel mutation in the MYOC gene in primary open glaucoma patients. J Med Genet 37: 301–303. 10819638 10.1136/jmg.37.4.301 PMC1734562

[101] Wang X, Sam-Wah TS, Ng YK (2000) Nitric oxide, microglial activities and neuronal cell death in the lateral geniculate nucleus of glaucomatous rats. Brain Res 878: 136–147. 10996144 10.1016/s0006-8993(00)02727-x

[102] Acharya M, Mookherjee S, Bhattacharjee A, Thakur SK, Bandyopadhyay AK, et al (2007) Evaluation of the OPTC gene in primary open angle glaucoma: functional significance of a silent change. BMC Mol Biol 8: 21. 17359525 10.1186/1471-2199-8-21 PMC1838427

[103] Kumar A, Basavaraj MG, Gupta SK, Qamar I, Ali AM, et al (2007) Role of CYP1B1, MYOC, OPTN, and OPTC genes in adult-onset primary open-angle glaucoma: predominance of CYP1B1 mutations in Indian patients. Mol Vis 13: 667–676. 17563717 PMC2765475

[104] Wawrocka A, Budny B, Debicki S, Jamsheer A, Sowinska A, et al (2012) PAX6 3′ deletion in a family with aniridia. Ophthalmic Genet 33: 44–48. 21985185 10.3109/13816810.2011.615076

[105] Dinour D, Chang MH, Satoh J, Smith BL, Angle N, et al (2004) A novel missense mutation in the sodium bicarbonate cotransporter (NBCe1/SLC4A4) causes proximal tubular acidosis and glaucoma through ion transport defects. J Biol Chem 279: 52238–52246. 15471865 10.1074/jbc.M406591200

[106] Igarashi T, Inatomi J, Sekine T, Seki G, Shimadzu M, et al (2001) Novel nonsense mutation in the Na+/HCO3- cotransporter gene (SLC4A4) in a patient with permanent isolated proximal renal tubular acidosis and bilateral glaucoma. J Am Soc Nephrol 12: 713–718. 11274232 10.1681/ASN.V124713

[107] Miyara N, Shinzato M, Yamashiro Y, Iwamatsu A, Kariya K, et al (2008) Proteomic analysis of rat retina in a steroid-induced ocular hypertension model: potential vulnerability to oxidative stress. Jpn J Ophthalmol 52: 84–90. 18626730 10.1007/s10384-007-0507-5

[108] Yuki K, Ozawa Y, Yoshida T, Kurihara T, Hirasawa M, et al (2011) Retinal ganglion cell loss in superoxide dismutase 1 deficiency. Invest Ophthalmol Vis Sci 52: 4143–4150. 21421868 10.1167/iovs.10-6294

[109] Tripathi RC, Borisuth NS, Tripathi BJ, Gotsis SS (1992) Quantitative and qualitative analyses of transferrin in aqueous humor from patients with primary and secondary glaucomas. Invest Ophthalmol Vis Sci 33: 2866–2873. 1526736

[110] Takai Y, Tanito M, Ohira A (2012) Multiplex cytokine analysis of aqueous humor in eyes with primary open-angle glaucoma, exfoliation glaucoma, and cataract. Invest Ophthalmol Vis Sci 53: 241–247. 22159018 10.1167/iovs.11-8434

[111] Fuchshofer R, Tamm ER (2012) The role of TGF-beta in the pathogenesis of primary open-angle glaucoma. Cell Tissue Res 347: 279–290. 22101332 10.1007/s00441-011-1274-7

[112] Agapova OA, Kaufman PL, Lucarelli MJ, Gabelt BT, Hernandez MR (2003) Differential expression of matrix metalloproteinases in monkey eyes with experimental glaucoma or optic nerve transection. Brain Res 967: 132–143. 12650974 10.1016/s0006-8993(02)04234-8

[113] van Koolwijk LM, Ramdas WD, Ikram MK, Jansonius NM, Pasutto F, et al (2012) Common Genetic Determinants of Intraocular Pressure and Primary Open-Angle Glaucoma. PLoS Genet 8: e1002611. 22570627 10.1371/journal.pgen.1002611 PMC3342933

[114] Libby RT, Smith RS, Savinova OV, Zabaleta A, Martin JE, et al (2003) Modification of ocular defects in mouse developmental glaucoma models by tyrosinase. Science 299: 1578–1581. 12624268 10.1126/science.1080095

[115] Pelis RM, Shahidullah M, Ghosh S, Coca-Prados M, Wright SH, et al (2009) Localization of Multidrug Resistance-Associated Protein 2 in the Nonpigmented Ciliary Epithelium of the Eye. Journal of Pharmacology and Experimental Therapeutics 329: 479–485. 19201990 10.1124/jpet.108.149625 PMC2672870

[116] Sonsino J, Gong HY, Wu P, Freddo TF (2002) Co-localization of junction-associated proteins of the human blood-aqueous barrier: Occludin, ZO-1 and F-actin. Experimental Eye Research 74: 123–129. 11878825 10.1006/exer.2001.1100

[117] Hasegawa H, Lian SC, Finkbeiner WE, Verkman AS (1994) Extrarenal tissue distribution of CHIP28 water channels by in situ hybridization and antibody staining. Am J Physiol 266: C893–C903. 7513954 10.1152/ajpcell.1994.266.4.C893

[118] Wetzel RK, Sweadner KJ (2001) Immunocytochemical localization of NaK-ATPase isoforms in the rat and mouse ocular ciliary epithelium. Investigative Ophthalmology & Visual Science 42: 763–769. 11222539

[119] Ghosh S, Freitag AC, Martinvasallo P, Cocaprados M (1990) Cellular-Distribution and Differential Gene-Expression of the 3 Alpha-Subunit Isoforms of the Na,K-Atpase in the Ocular Ciliary Epithelium. Journal of Biological Chemistry 265: 2935–2940. 1689295

[120] Zhang Y, Patil RV, Marmorstein AD (2010) Bestrophin 2 is expressed in human non-pigmented ciliary epithelium but not retinal pigment epithelium. Molecular Vision 16: 200–206. 20157619 PMC2820107

[121] Zenkel M, Kruse FE, Junemann AG, Naumann GOH, Schlotzer-Schrehardt U (2006) Clusterin deficiency in eyes with pseudoexfoliation syndrome may be implicated in the aggregation and deposition of pseudoexfoliative material. Investigative Ophthalmology & Visual Science 47: 1982–1990. 16639006 10.1167/iovs.05-1580

[122] Saito K, Yonezawa T, Minaguchi J, Kurosaki M, Suetsugu S, et al.. (2010) Distribution of alpha(IV) collagen chains in the ocular anterior segments of adult mice. Connective Tissue Research 1–10. 10.3109/03008207.2010.492062 20672978

[123] Ostojic J, Grozdanic S, Syed NA, Hargrove MS, Trent JT, et al (2008) Neuroglobin and Cytoglobin Distribution in the Anterior Eye Segment: A Comparative Immunohistochemical Study. Journal of Histochemistry & Cytochemistry 56: 863–872. 18574250 10.1369/jhc.2008.951392 PMC2516955

[124] Bejjani BA, Xu L, Armstrong D, Lupski JR, Reneker LW (2002) Expression patterns of cytochrome P4501B1 (Cyp1b1) in FVB/N mouse eyes. Experimental Eye Research 75: 249–257. 12384088

[125] Doshi M, Marcus C, Bejjani BA, Edward DP (2006) Immunolocalization of CYP1B1 in normal, human, fetal and adult eyes. Experimental Eye Research 82: 24–32. 15979611 10.1016/j.exer.2005.04.016

[126] Calera MR, Topley HL, Liao YB, Duling BR, Paul DL, et al (2006) Connexin43 is required for production of the aqueous humor in the murine eye. Journal of Cell Science 119: 4510–4519. 17046998 10.1242/jcs.03202

[127] Coca-Prados M, Ghosh S, Gilula NB, Kumar NM (1992) Expression and Cellular-Distribution of the Alpha-1 Gap Junction Gene-Product in the Ocular Pigmented Ciliary Epithelium. Current Eye Research 11: 113–122. 10.3109/02713689209000061 1374005

[128] Coffey KL, Krushinsky A, Green CR, Donaldson PJ (2002) Molecular profiling and cellular localization of connexin isoforms in the rat ciliary epithelium. Experimental Eye Research 75: 9–21. 12123633 10.1006/exer.2002.1187

[129] Tserentsoodol N, Shin BC, Suzuki T, Takata K (1998) Colocalization of tight junction proteins, occludin and ZO-1, and glucose transporter GLUT1 in cells of the blood-ocular barrier in the mouse eye. Histochemistry and Cell Biology 110: 543–551. 9860252 10.1007/s004180050316

[130] Wang TH, Lindsey JD, Weinreb RN (1994) Laminin Subtype Distribution in the Human Ciliary Body. Investigative Ophthalmology & Visual Science 35: 3776–3782. 8088965

[131] Lan J, Kumar RK, Di Girolamo N, McCluskey P, Wakefield D (2003) Expression and distribution of matrix metalloproteinases and their inhibitors in the human iris and ciliary body. British Journal of Ophthalmology 87: 208–211. 12543753 10.1136/bjo.87.2.208 PMC1771518

[132] Bertazolli-Filho R, Coca-Prados M, Haddad A, Laicine EM (2007) Molecular analysis of neurolysin expression in the rat and bovine ciliary body. Current Eye Research 32: 751–756. 17882707 10.1080/02713680701573381

[133] Ortego J, Coca- Prados M (1997) Molecular characterization and differential gene induction of the neuroendocrine-specific genes neurotensin, neurotensin receptor, PC1, PC2, and 7B2 in the human ocular ciliary epithelium. Journal of Neurochemistry 69: 1829–1839. 9349525 10.1046/j.1471-4159.1997.69051829.x

[134] Wu P, Gong HY, Richman R, Freddo TF (2000) Localization of occludin, ZO-1, and pan-cadherin in rabbit ciliary epithelium and iris vascular endothelium. Histochemistry and Cell Biology 114: 303–310. 11131095 10.1007/s004180000195

[135] Ortego J, Escribano J, Becerra SP, Cocaprados M (1996) Gene expression of the neurotrophic pigment epithelium-derived factor in the human ciliary epithelium - Synthesis and secretion into the aqueous humor. Investigative Ophthalmology & Visual Science 37: 2759–2767. 8977492

[136] Gerashchenko DY, Beuckmann CT, Marcheselli VL, Gordon WC, Kanaoka Y, et al (1998) Localization of lipocalin-type prostaglandin D synthase (beta-trace) in iris, ciliary body, and eye fluids. Investigative Ophthalmology & Visual Science 39: 198–203. 9430563

[137] Bertazolli-Filho R, Ghosh S, Huang WH, Wollmann G, Coca-Prados M (2001) Molecular evidence that human ocular ciliary epithelium expresses components involved in phototransduction. Biochemical and Biophysical Research Communications 284: 317–325. 11394879 10.1006/bbrc.2001.4970

[138] Martin-Alonso JM, Ghosh S, Hernando N, Crabb JW, Cocaprados M (1993) Differential Expression of the Cellular Retinaldehyde-Binding Protein in Bovine Ciliary Epithelium. Experimental Eye Research 56: 659–669. 8595808 10.1006/exer.1993.1083

[139] Salvador-Silva M, Ghosh S, Bertazolli R, Boatright JH, Nickerson JM, et al (2005) Retinoid processing proteins in the ocular ciliary epithelium. Molecular Vision 11: 356–365. 15928609

[140] Ortego J, Escribano J, Crabb J, Cocaprados M (1996) Identification of a neuropeptide and neuropeptide-processing enzymes in aqueous humor confers neuroendocrine features to the human ocular ciliary epithelium. Journal of Neurochemistry 66: 787–796. 8592153 10.1046/j.1471-4159.1996.66020787.x

[141] Rauz S, Walker EA, Hughes SV, Coca-Prados M, Hewison M, et al (2003) Serum- and glucocorticoid-regulated kinase isoform-1 and epithelial sodium channel subunits in human ocular ciliary epithelium. Investigative Ophthalmology & Visual Science 44: 1643–1651. 12657604 10.1167/iovs.02-0514

[142] Hochgesand DH, Dunn JJ, Crook RB (2001) Catecholaminergic regulation of Na-K-Cl cotransport in pigmented ciliary epithelium: differences between PE and NPE. Exp Eye Res 72: 1–12. 11133177 10.1006/exer.2000.0927

[143] Dunn JJ, Lytle C, Crook RB (2001) Immunolocalization of the Na-K-Cl cotransporter in bovine ciliary epithelium. Investigative Ophthalmology & Visual Science 42: 343–353. 11157865

[144] Bok D, Schibler MJ, Pushkin A, Sassani P, Abuladze N, et al (2001) Immunolocalization of electrogenic sodium-bicarbonate cotransporters pNBC1 and kNBC1 in the rat eye. American Journal of Physiology-Renal Physiology 281: F920–F935. 11592950 10.1152/ajprenal.2001.281.5.F920

[145] Gao B, Huber RD, Wenzel A, Vavricka SR, Ismair MG, et al (2005) Localization of organic anion transporting polypeptides in the rat. and human ciliary body epithelium. Experimental Eye Research 80: 61–72. 15652527 10.1016/j.exer.2004.08.013

[146] Bertazolli R, Laicine EM, Haddad A (2003) Synthesis and secretion of transferrin by isolated ciliary epithelium of rabbit. Biochemical and Biophysical Research Communications 305: 820–825. 12767904 10.1016/s0006-291x(03)00825-8

[147] Peress NS, Perillo E (1994) Tgf and Tgf-Beta-3 Immunoreactivity Within the Ciliary Epithelium. Investigative Ophthalmology & Visual Science 35: 453–457. 8112993

