# Supplementary figures and images for: Gene Expression and Functional Annotation of the Human Ciliary Body Epithelia
Source: PLoS One. 2012 Sep 18;7(9):e44973. doi: 10.1371/journal.pone.0044973 (PMC3445623; doi:10.1371/journal.pone.0044973)

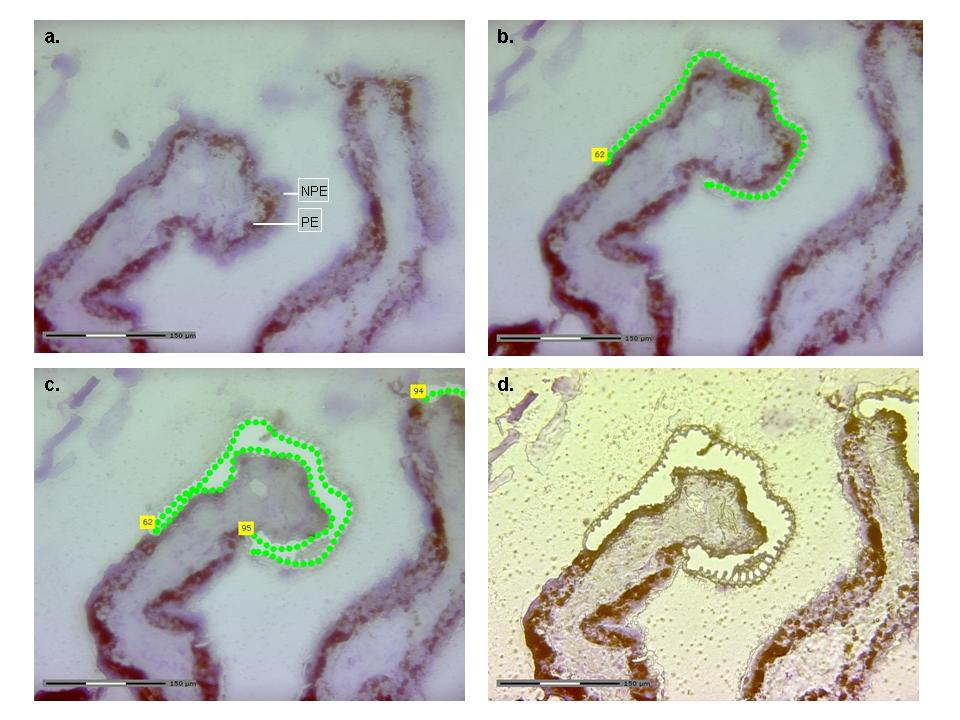

Supplement: Figure S1 — Separate laser dissection microscopy of the NPE and PE. Separate cut out of the non-pigmented (NPE) and pigmented epithelium (PE) from the ciliary body by laser dissection. Frozen sections of 20 µm were stained with cresyl violet to distinguish the NPE (a). Next, the NPE was cut out with laser and collected in a tube. The green dotted line is the selection which the laser will cut. The number 62 refers to the 62th selection that was made (b). Subsequently, the PE was cut out (c). The stroma remains after laser dissection of NPE and PE (d). (TIF) [file pone.0044973.s001.tif]

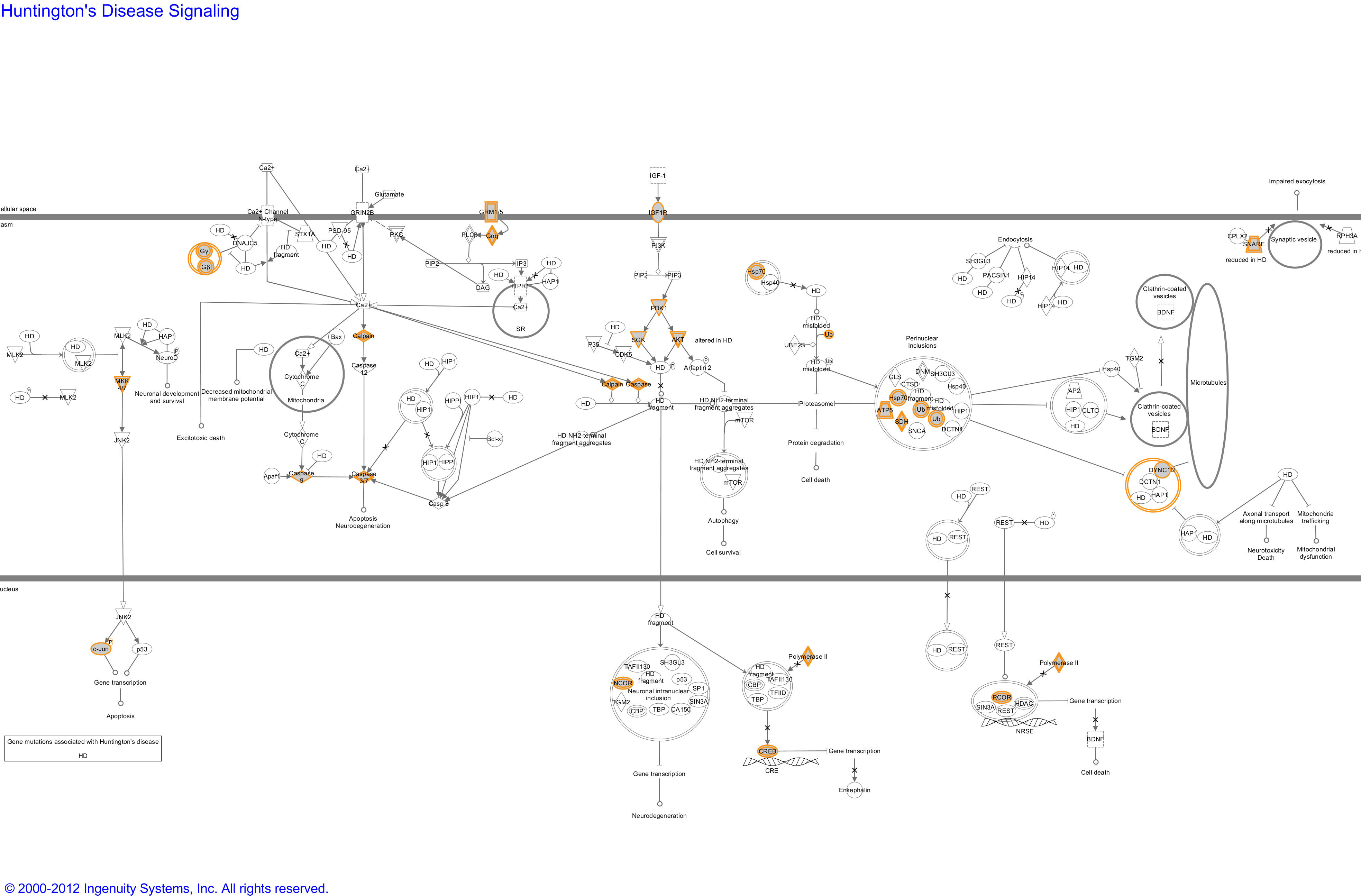

Supplement: Figure S2 — Huntington’s disease signaling pathway identified by the Ingenuity software. This is one of the canonical pathways that contains statistically significantly more genes than expected by chance in the group of genes that are highly expressed in the non-pigmented epithelium (NPE) of the ciliary body. Gray fields indicate their presence among the highest expressed genes of the NPE; uncolored genes are added by the Ingenuity software to form the pathway. Solid lines between molecules indicate direct physical relationships between molecules (such as regulating and interacting protein domains); dotted lines indicate indirect functional relationships (such as co-regulation of expression of both genes in cell lines). Abbreviations of gene names are according to standard abbreviations used in Genbank. (JPG) [file pone.0044973.s002.jpg]

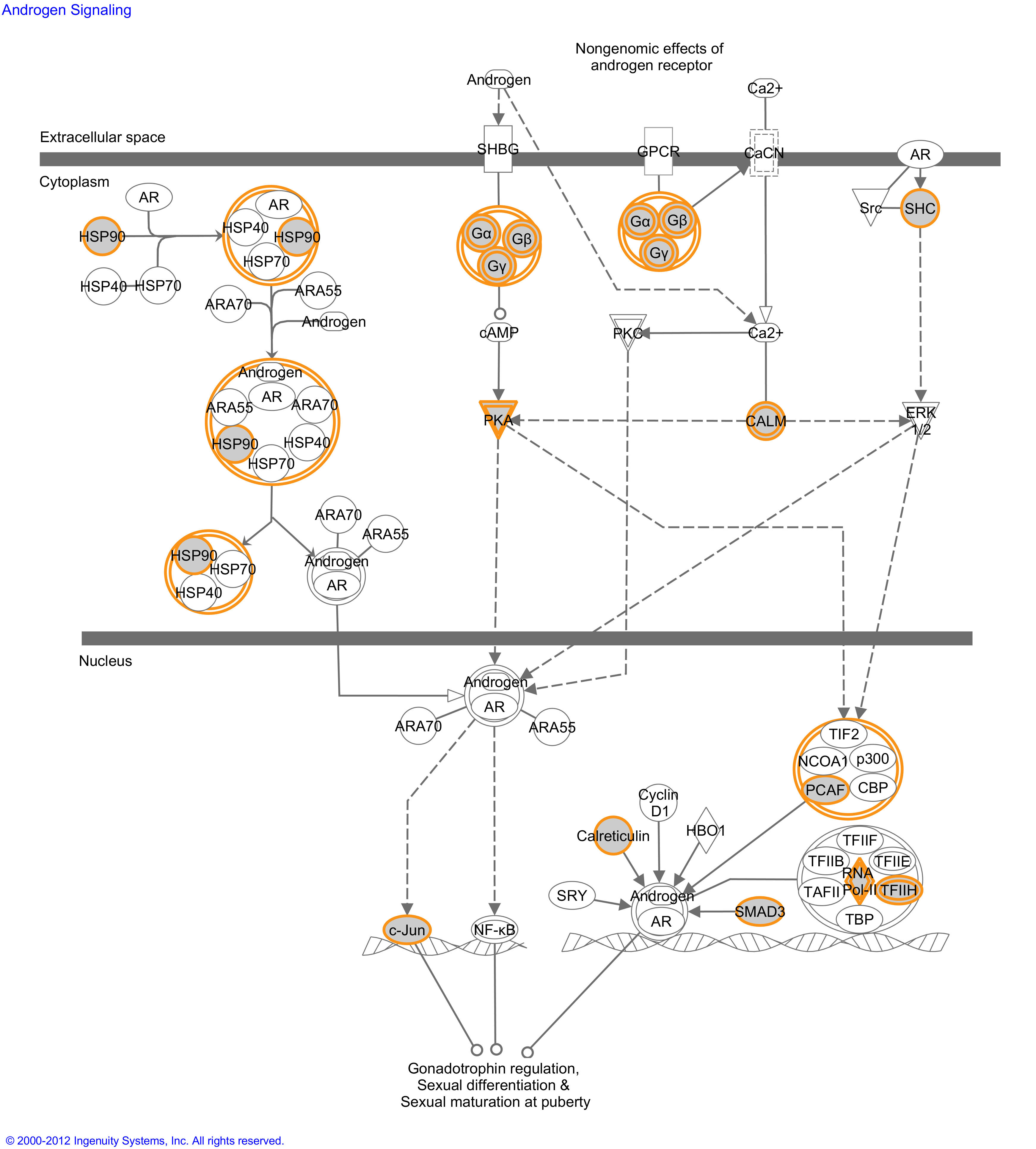

Supplement: Figure S3 — Androgen signaling pathway identified by the Ingenuity software. This is one of the canonical pathways that contains statistically significantly more genes than expected by chance in the group of genes that are highly expressed in the non-pigmented epithelium (NPE) of the ciliary body. For explanation of symbols on the diagrams see legend Figure S2. (JPG) [file pone.0044973.s003.jpg]

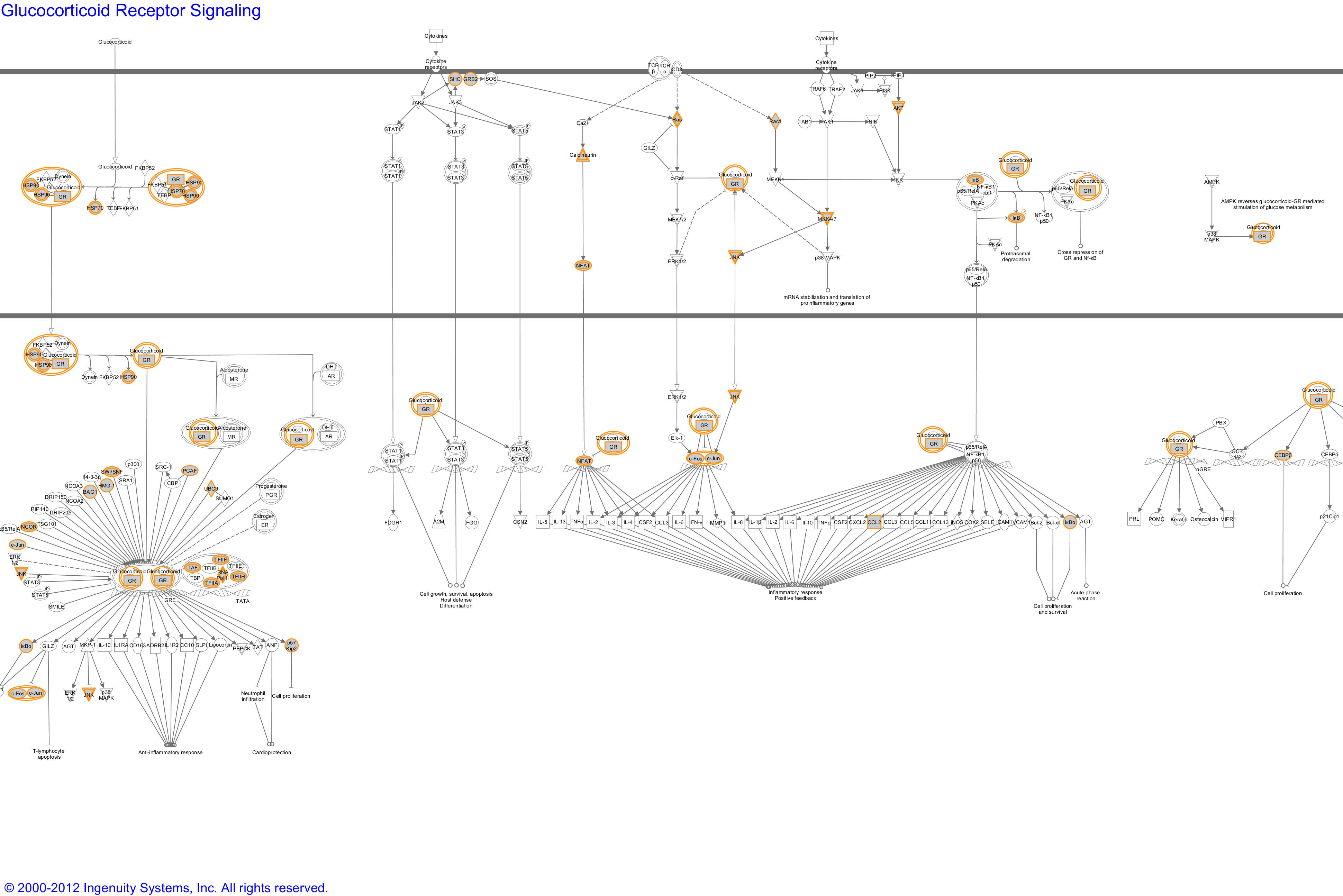

Supplement: Figure S4 — Glucocorticoid receptor signaling pathway identified by the Ingenuity software. This is one of the canonical pathways that contains statistically significantly more genes than expected by chance in the group of genes that are highly expressed in the non-pigmented epithelium (NPE) of the ciliary body. For explanation of symbols on the diagrams see legend Figure S2. (JPG) [file pone.0044973.s004.jpg]

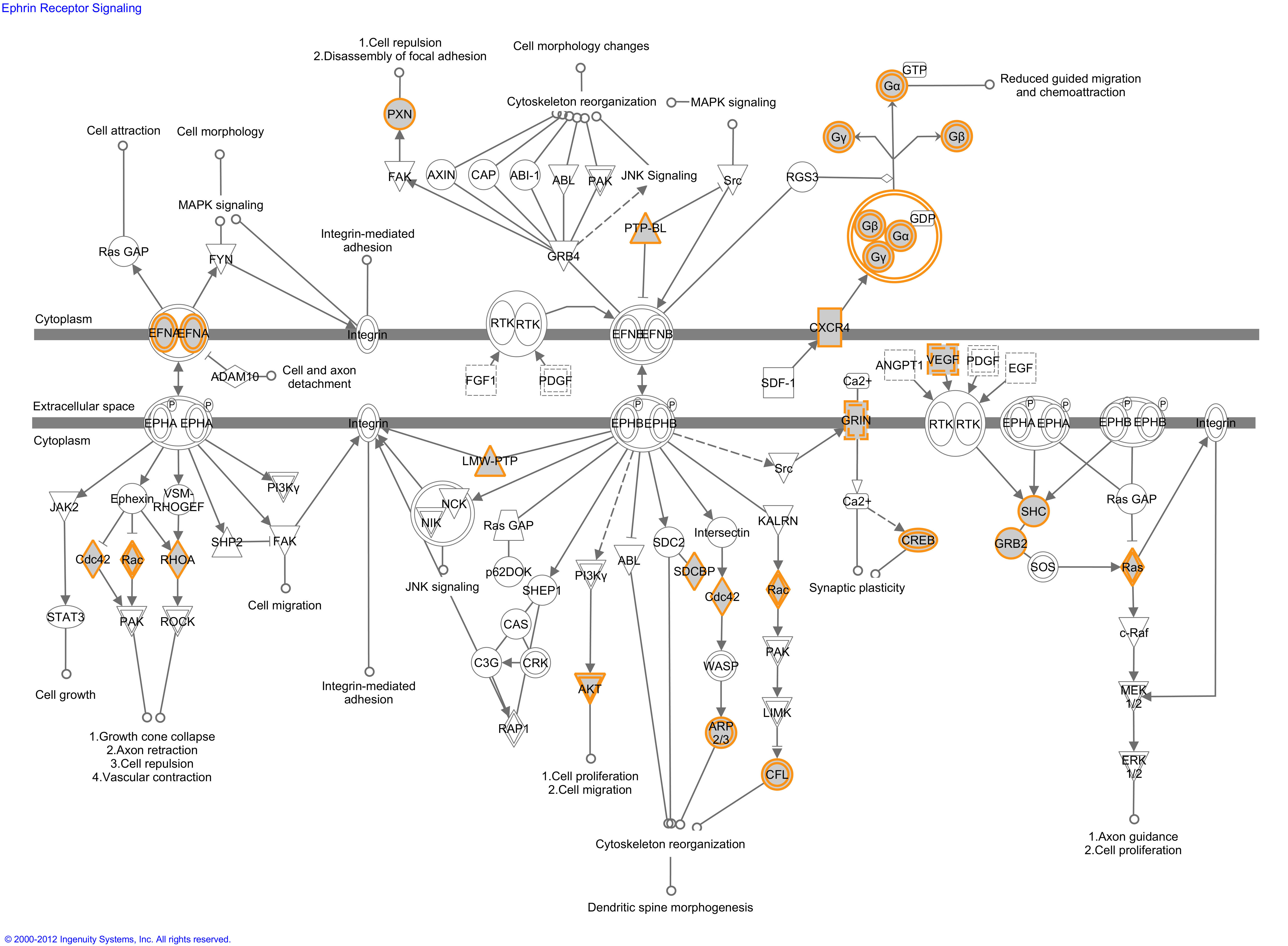

Supplement: Figure S5 — Ephrin receptor signaling pathway identified by the Ingenuity software. This is one of the canonical pathways that contains statistically significantly more genes than expected by chance in the group of genes that are highly expressed in the non-pigmented epithelium (NPE) of the ciliary body. For explanation of symbols on the diagrams see legend Figure S2. (JPG) [file pone.0044973.s005.jpg]

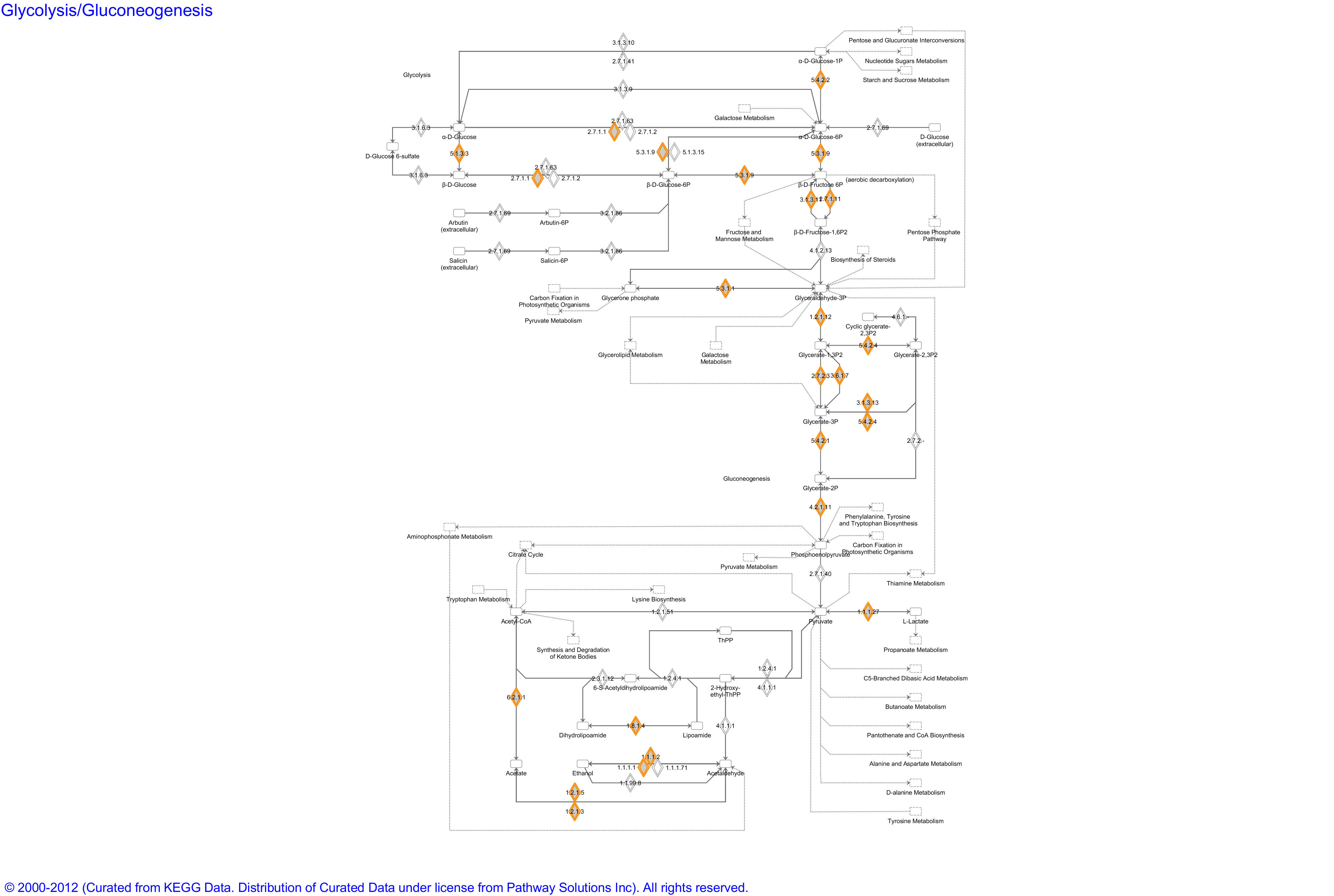

Supplement: Figure S6 — Glycolysis/gluconeogenesis pathway identified by the Ingenuity software. This is one of the canonical pathways that contains statistically significantly more genes than expected by chance in the group of genes that are highly expressed in the non-pigmented epithelium (NPE) of the ciliary body. For explanation of symbols on the diagrams see legend Figure S2. (JPG) [file pone.0044973.s006.jpg]

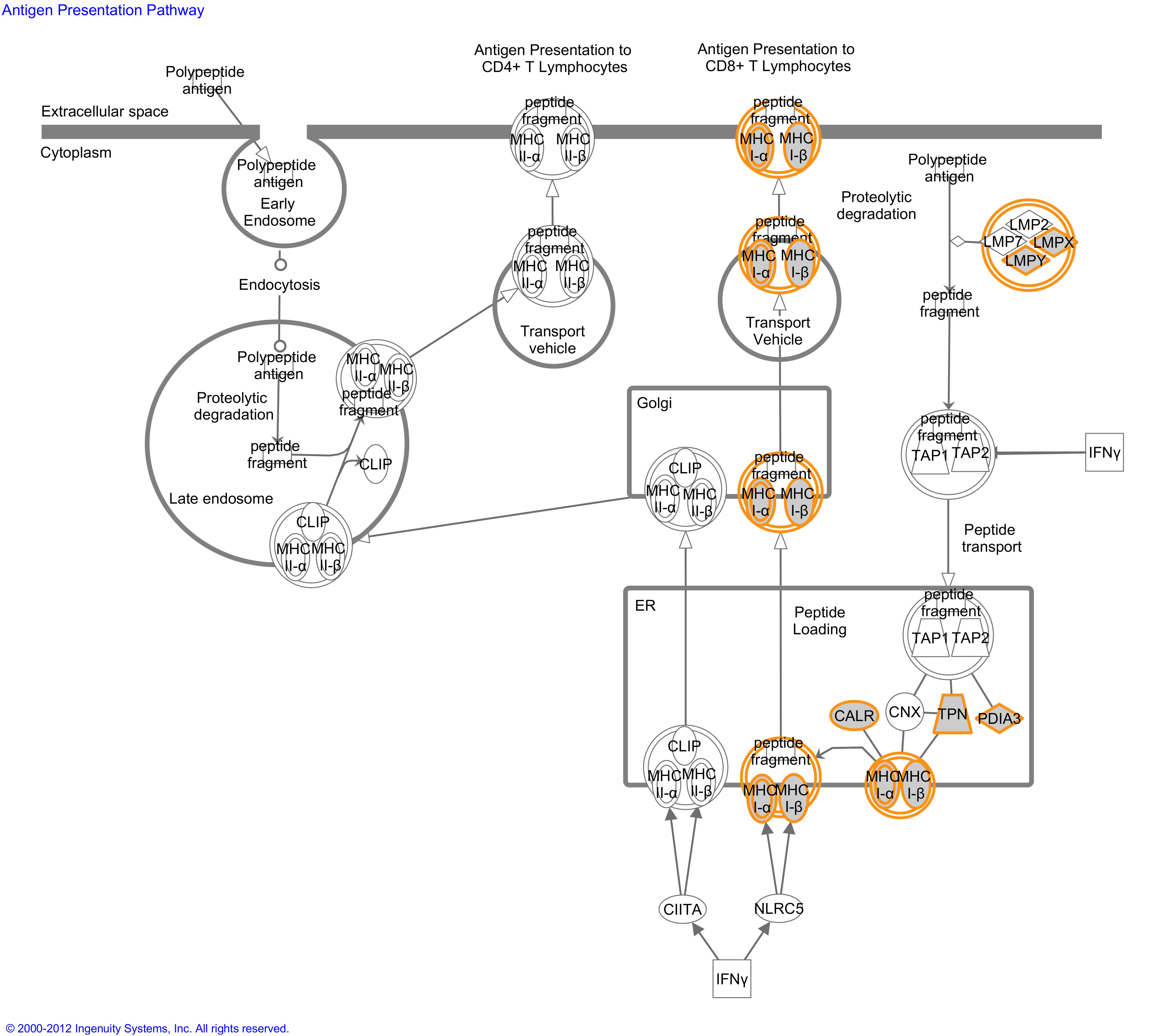

Supplement: Figure S7 — Antigen presentation pathway identified by the Ingenuity software. This is one of the canonical pathways that contains statistically significantly more genes than expected by chance in the group of genes that are highly expressed in the non-pigmented epithelium (NPE) of the ciliary body. For explanation of symbols on the diagrams see legend Figure S2. (JPG) [file pone.0044973.s007.jpg]

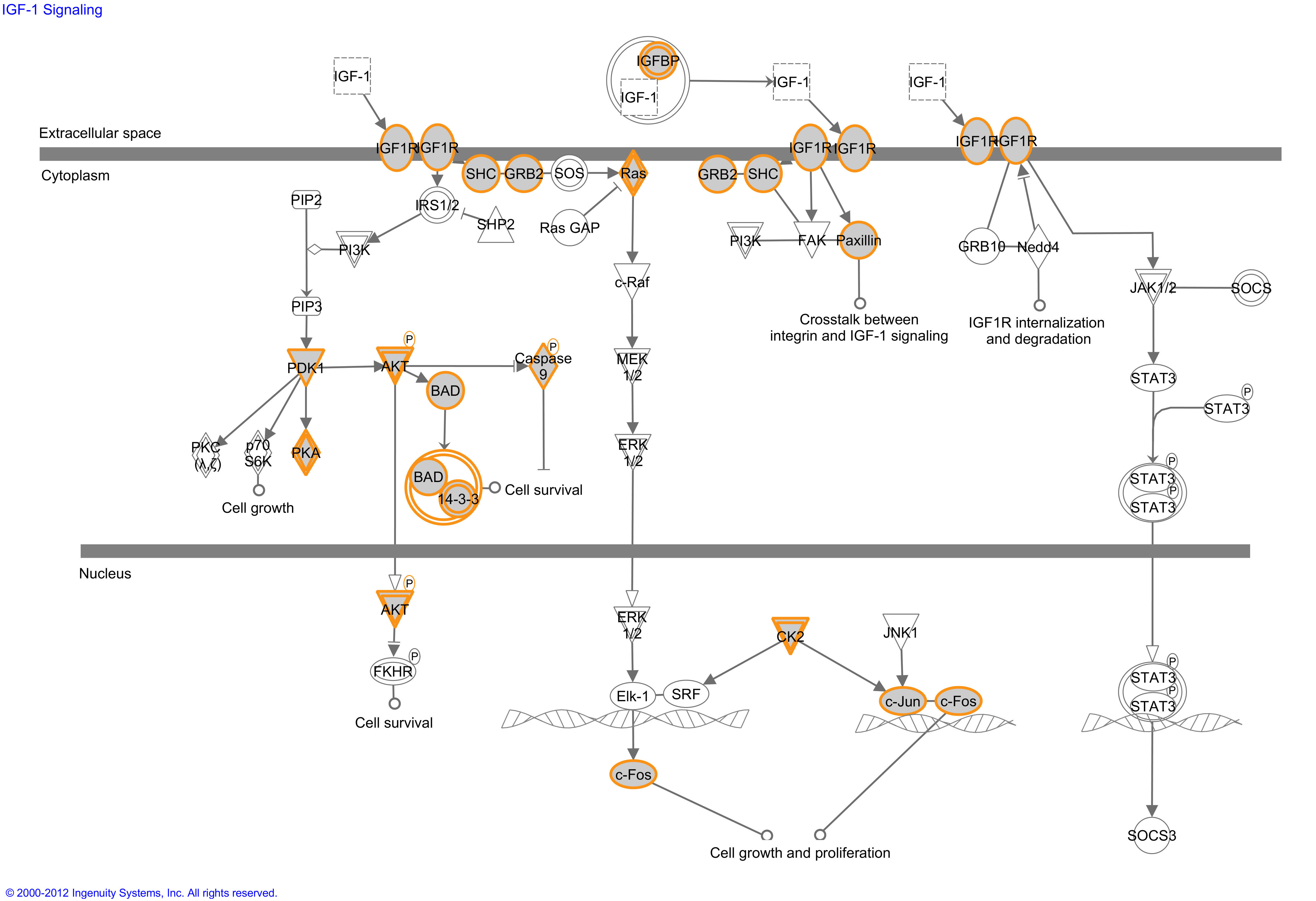

Supplement: Figure S8 — IGF-1 signaling pathway identified by the Ingenuity software. This is one of the canonical pathways that contains statistically significantly more genes than expected by chance in the group of genes that are highly expressed in the non-pigmented epithelium (NPE) of the ciliary body. For explanation of symbols on the diagrams see legend Figure S2. (JPG) [file pone.0044973.s008.jpg]

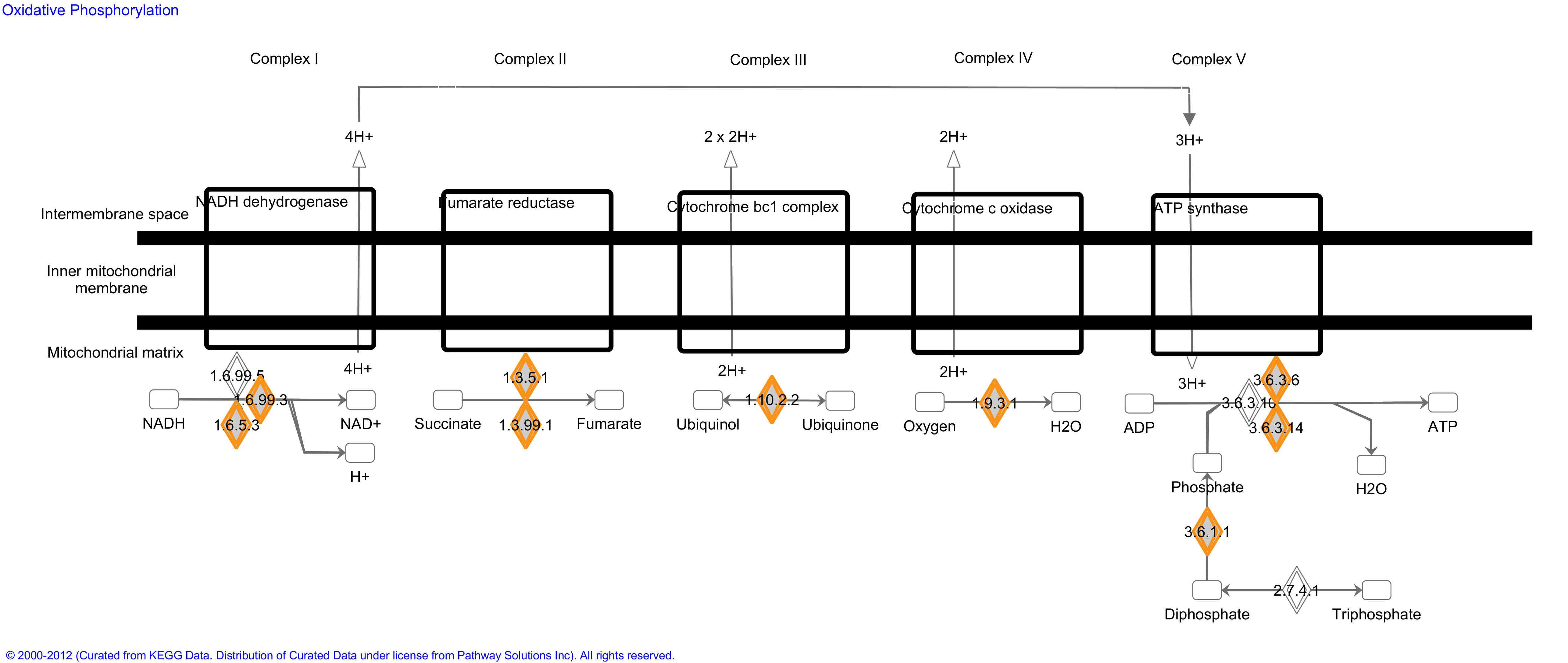

Supplement: Figure S9 — Oxidative phosphorylation pathway identified by the Ingenuity software. This is one of the canonical pathways that contains statistically significantly more genes than expected by chance in the group of genes that are highly expressed in the non-pigmented epithelium (NPE) of the ciliary body. For explanation of symbols on the diagrams see legend Figure S2. (JPG) [file pone.0044973.s009.jpg]

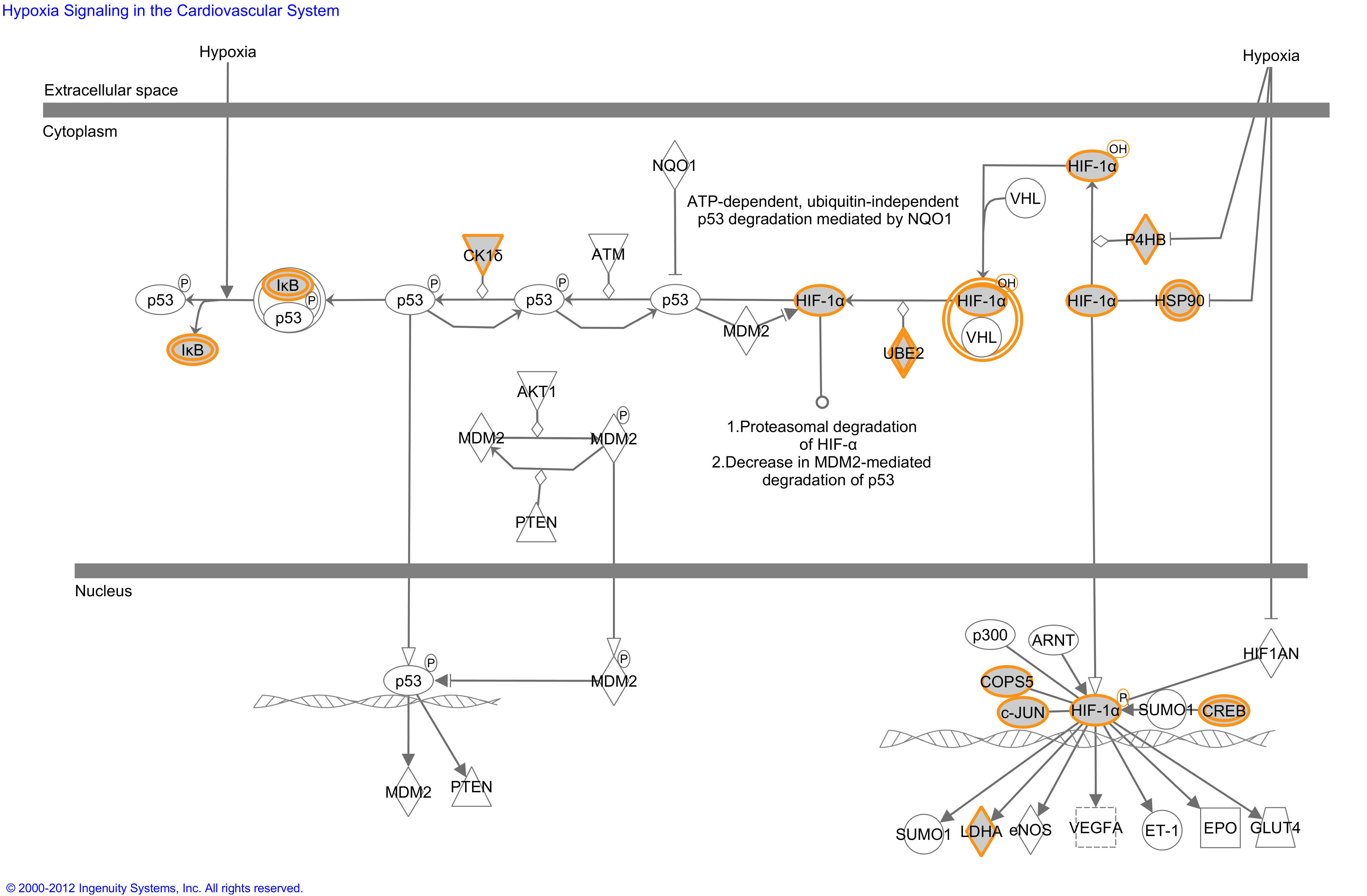

Supplement: Figure S10 — Hypoxia signaling in the cardiovascular system pathway identified by the Ingenuity software. This is one of the canonical pathways that contains statistically significantly more genes than expected by chance in the group of genes that are highly expressed in the non-pigmented epithelium (NPE) of the ciliary body. For explanation of symbols on the diagrams see legend Figure S2. (JPG) [file pone.0044973.s010.jpg]

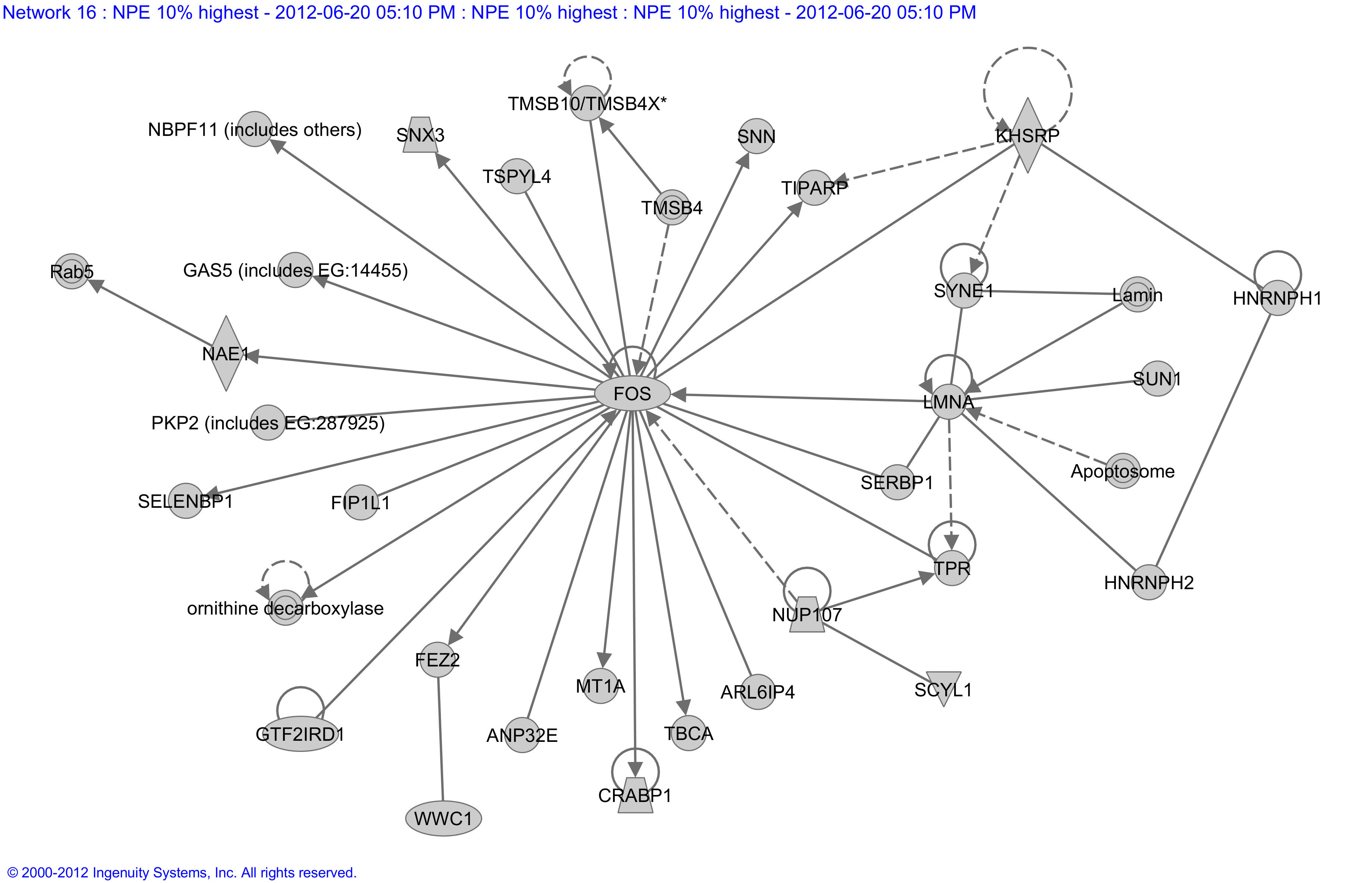

Supplement: Figure S11 — Molecular network generated by the Ingenuity software from the highest expressed genes of the NPE. Molecular network generated from our microarray data of the highest expressed genes of the non-pigmented epithelium (NPE). Grey symbols represent genes that are highly expressed in the NPE of the ciliary body. Transparent entries are molecules from the knowledge database, inserted to connect all relevant molecules in a single network. Solid lines between molecules indicate direct physical relationships between molecules (such as regulating and interacting protein domains); dotted lines indicate indirect functional relationships (such as co-regulation of expression of both genes in cell lines). Abbreviations of gene names are according to standard abbreviations used in Genbank. The main functionalities given by Ingenuity for this molecular network are ‘Cellular development, cellular growth and proliferation, renal proliferation’. Of interest is that this network is mainly build up around the gene FOS. FOS proteins have been implicated as regulators of cell proliferation, differentiation and transformation during development (JPG) [file pone.0044973.s011.jpg]

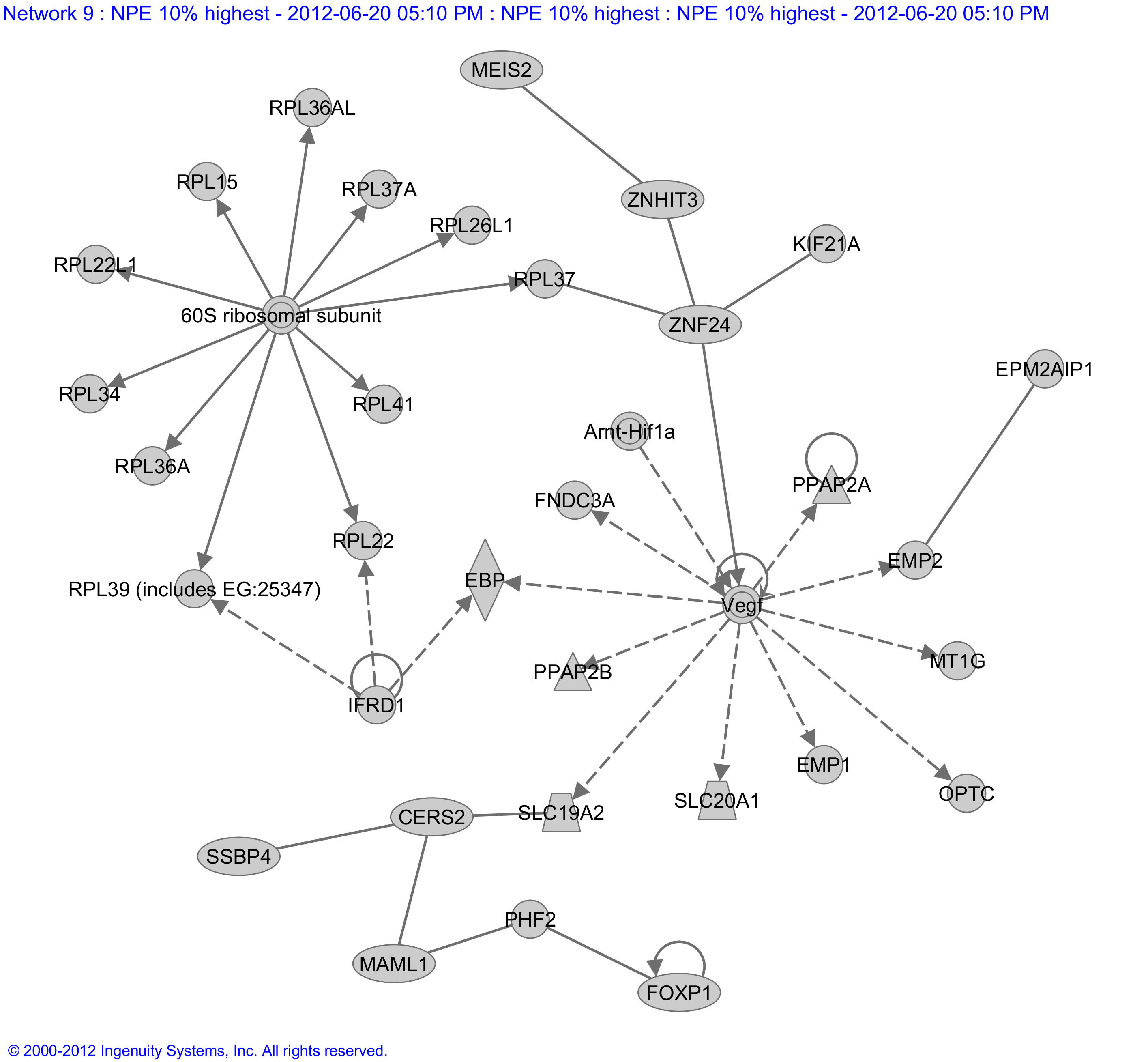

Supplement: Figure S12 — Molecular network generated by the Ingenuity software from the highest expressed genes of the NPE. Molecular network generated from our microarray data of the highest expressed genes of the non-pigmented epithelium (NPE). For explanation of symbols on the diagrams see legend Figure S11. The main functionalities given by Ingenuity for this molecular network are ‘Cancer, reproductive system disease, carbohydrate metabolism’. Highlights in this network are the molecules MEIS2, MAML1 and FOXP1 that are all involved in developmental processes. Moreover, this network contained the gene OPTC, which have been previously attributed to glaucoma. (JPG) [file pone.0044973.s012.jpg]

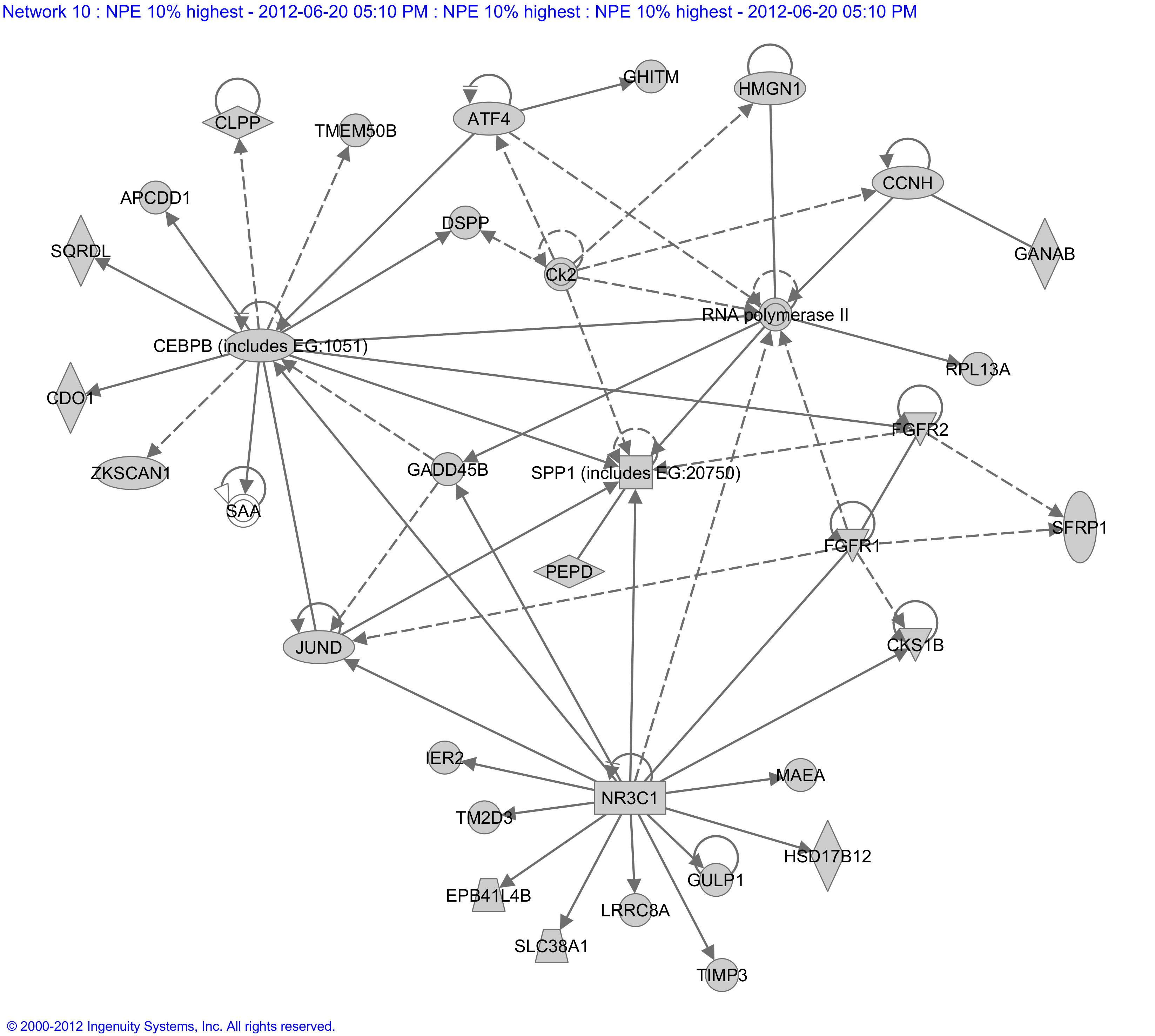

Supplement: Figure S13 — Molecular network generated by the Ingenuity software from the highest expressed genes of the NPE. Molecular network generated from our microarray data of the highest expressed genes of the non-pigmented epithelium (NPE). For explanation of symbols on the diagrams see legend Figure S11. The main functionalities given by Ingenuity for this molecular network are ‘Organismal development, tissue development, cellular development’. Highlights in this network are the molecules FGFR1, FGFR2, SFRP1 and CCNH that are all involved in developmental processes. (JPG) [file pone.0044973.s013.jpg]

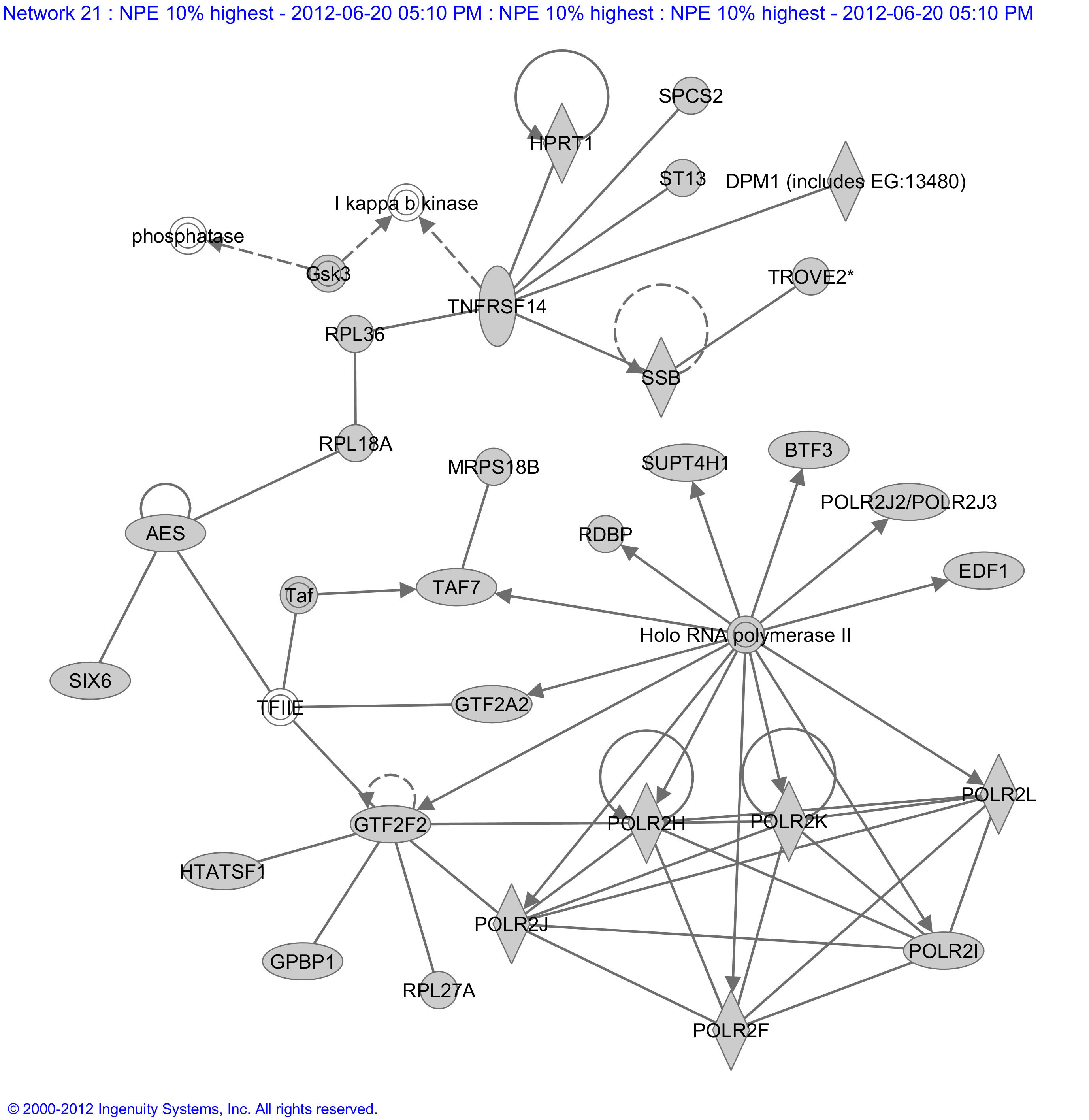

Supplement: Figure S14 — Molecular network generated by the Ingenuity software from the highest expressed genes of the NPE. Molecular network generated from our microarray data of the highest expressed genes of the non-pigmented epithelium (NPE). For explanation of symbols on the diagrams see legend Figure S11. The main functionalities given by Ingenuity for this molecular network are ‘Gene expression, infectious disease, embryonic development’. Highlights in this network are the molecules AES and SIX6 that are involved in developmental processes. Moreover, this network contained the gene TNFRSF14, which protein is involved in viral entry mechanisms. (JPG) [file pone.0044973.s014.jpg]

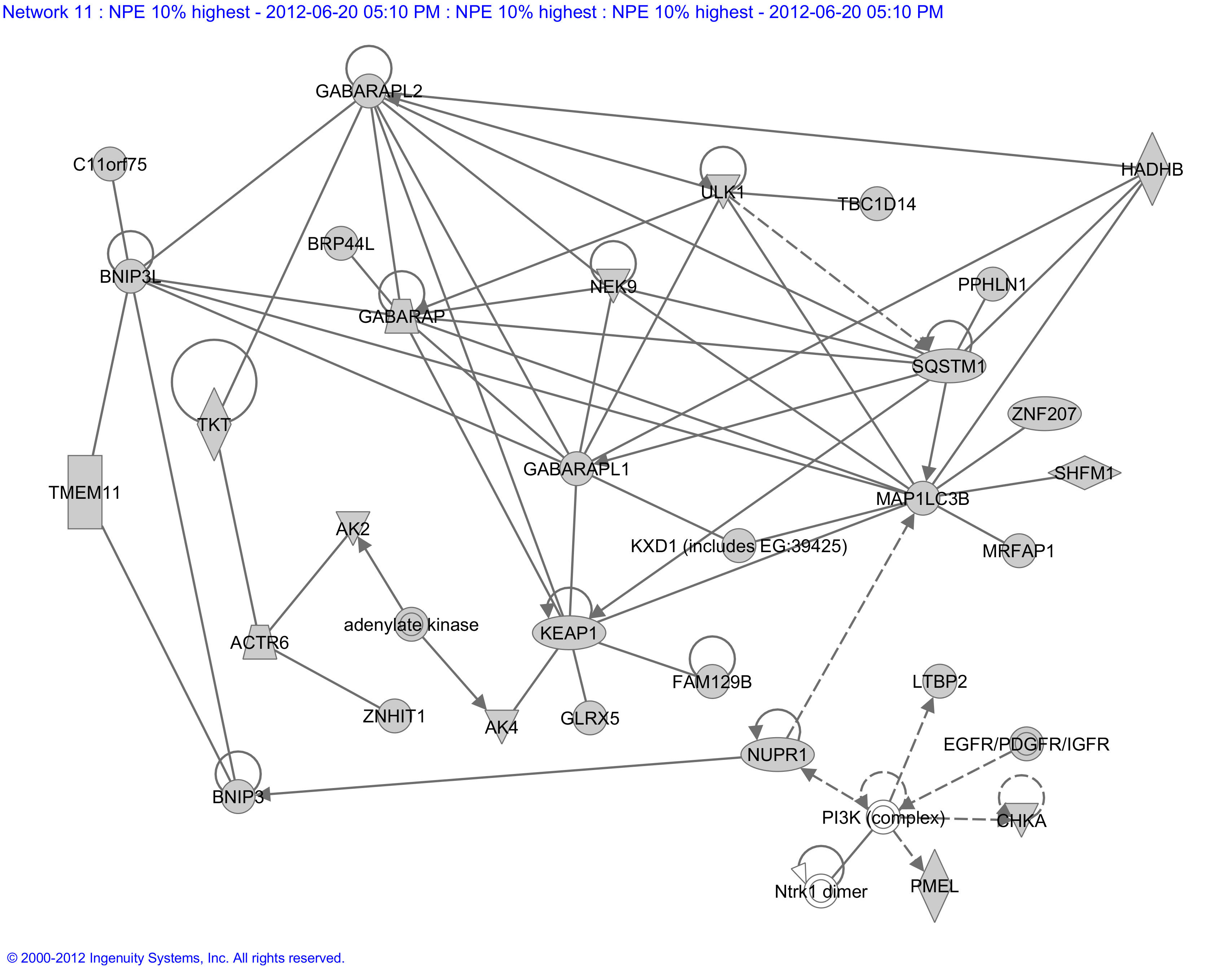

Supplement: Figure S15 — Molecular network generated by the Ingenuity software from the highest expressed genes of the NPE. Molecular network generated from our microarray data of the highest expressed genes of the non-pigmented epithelium (NPE). For explanation of symbols on the diagrams see legend Figure S11. The main functionalities given by Ingenuity for this molecular network are ‘Cell morphology, cellular function and maintenance, connective tissue development and function’. This network contained the gene MAP1LC3B, which protein product is involved in neurogenesis. Also three genes coding for GABA receptor associated proteins were present in this network, mediating inhibition of neurotransmission. This network is an example of the neural background we found in the NPE. (JPG) [file pone.0044973.s015.jpg]

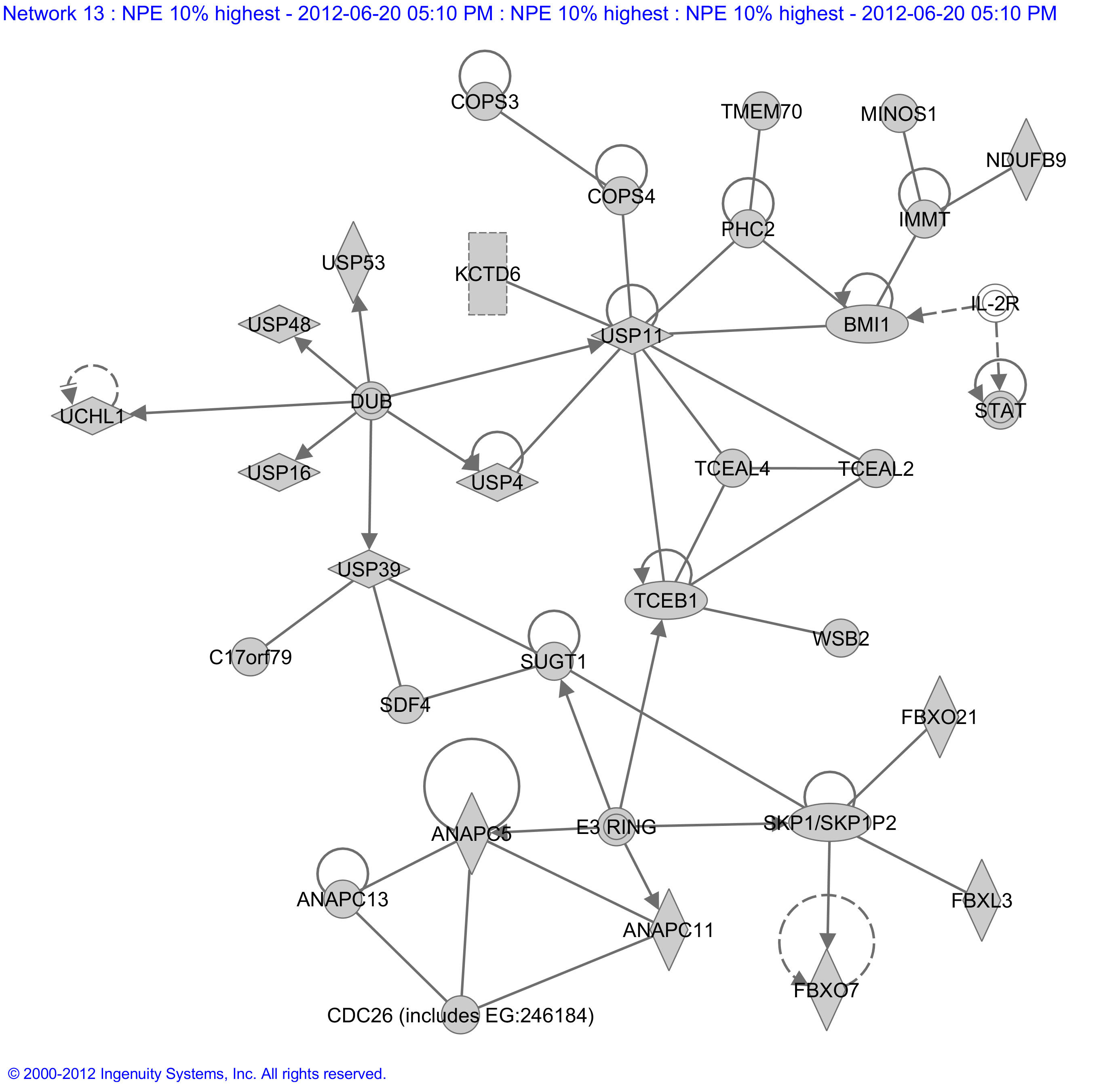

Supplement: Figure S16 — Molecular network generated by the Ingenuity software from the highest expressed genes of the NPE. Molecular network generated from our microarray data of the highest expressed genes of the non-pigmented epithelium (NPE). For explanation of symbols on the diagrams see legend Figure S11. The main functionalities given by Ingenuity for this molecular network are ‘Post-translational modification, connective tissue development and function, embryonic development’. In this network, we found the gene UCHL1, which is a neuron specific gene and previously associated with Parkinson’s disease. (JPG) [file pone.0044973.s016.jpg]

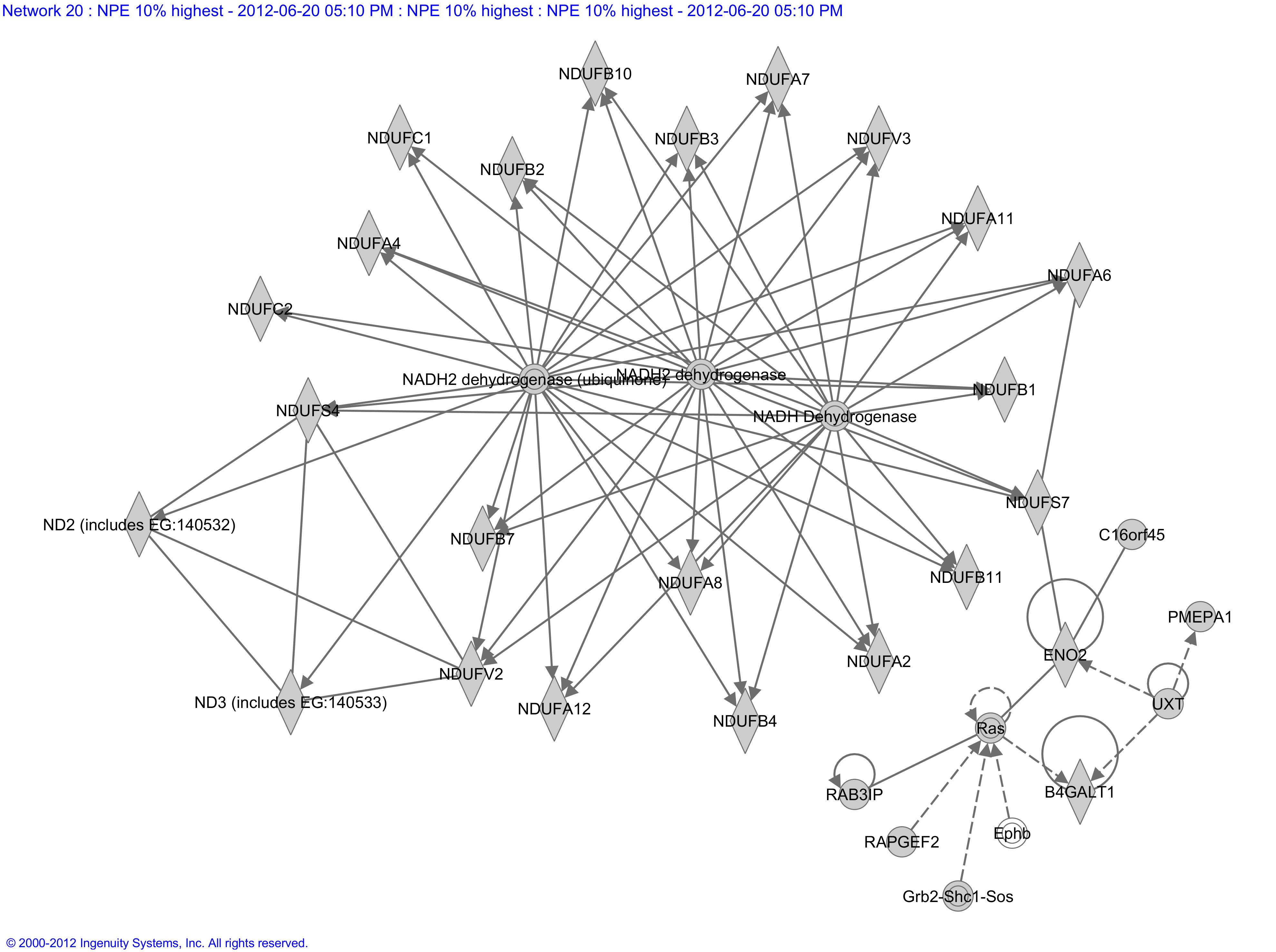

Supplement: Figure S17 — Molecular network generated by the Ingenuity software from the highest expressed genes of the NPE. Molecular network generated from our microarray data of the highest expressed genes of the non-pigmented epithelium (NPE). For explanation of symbols on the diagrams see legend Figure S11. The main functionalities given by Ingenuity for this molecular network are ‘Hereditary disorder, metabolic disease, cardiovascular disease’. This network contained several genes that have been associated with neurological diseases, for example Huntington’s disease (NDUFA7, NDUFC1, NDUFB2, NDUFB3, NDUFA8), atrophy of optic nerve (NDUFS4) and Leigh syndrome (NDUFS7, NDUFA2, NDUFS). This network is an example of the neural background we found in the NPE. (JPG) [file pone.0044973.s017.jpg]

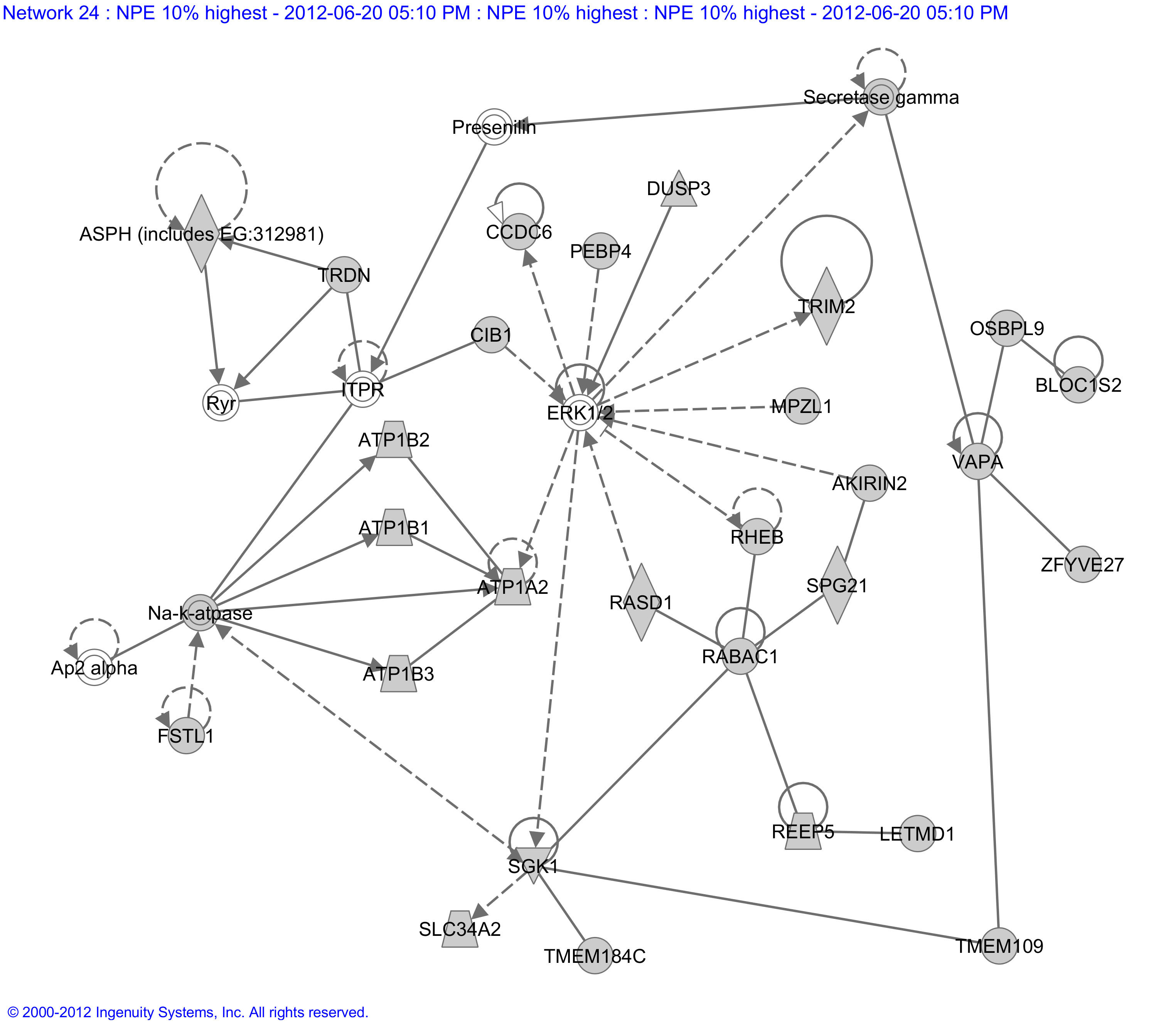

Supplement: Figure S18 — Molecular network generated by the Ingenuity software from the highest expressed genes of the NPE. Molecular network generated from our microarray data of the highest expressed genes of the non-pigmented epithelium (NPE). For explanation of symbols on the diagrams see legend Figure S11. The main functionalities given by Ingenuity for this molecular network are ‘Molecular transport, neurological disease, connective tissue disorders’. This network contained two genes previously associated with neurological diseases, namely ZFYVE27 (hereditary spastic paraplegia) and SPG21 (neurodegeneration of photoreceptors). Also, we found genes involved in nervous system development, for example ATP1B1 (involved in aggregation of neurons), TRIM2 (neuroprotection of cortical neurons) and VAPA, SGK1 and ZFYVE27 (involved in morphogenesis of neuritis). This network is an example of the neural background we found in the NPE. (JPG) [file pone.0044973.s018.jpg]

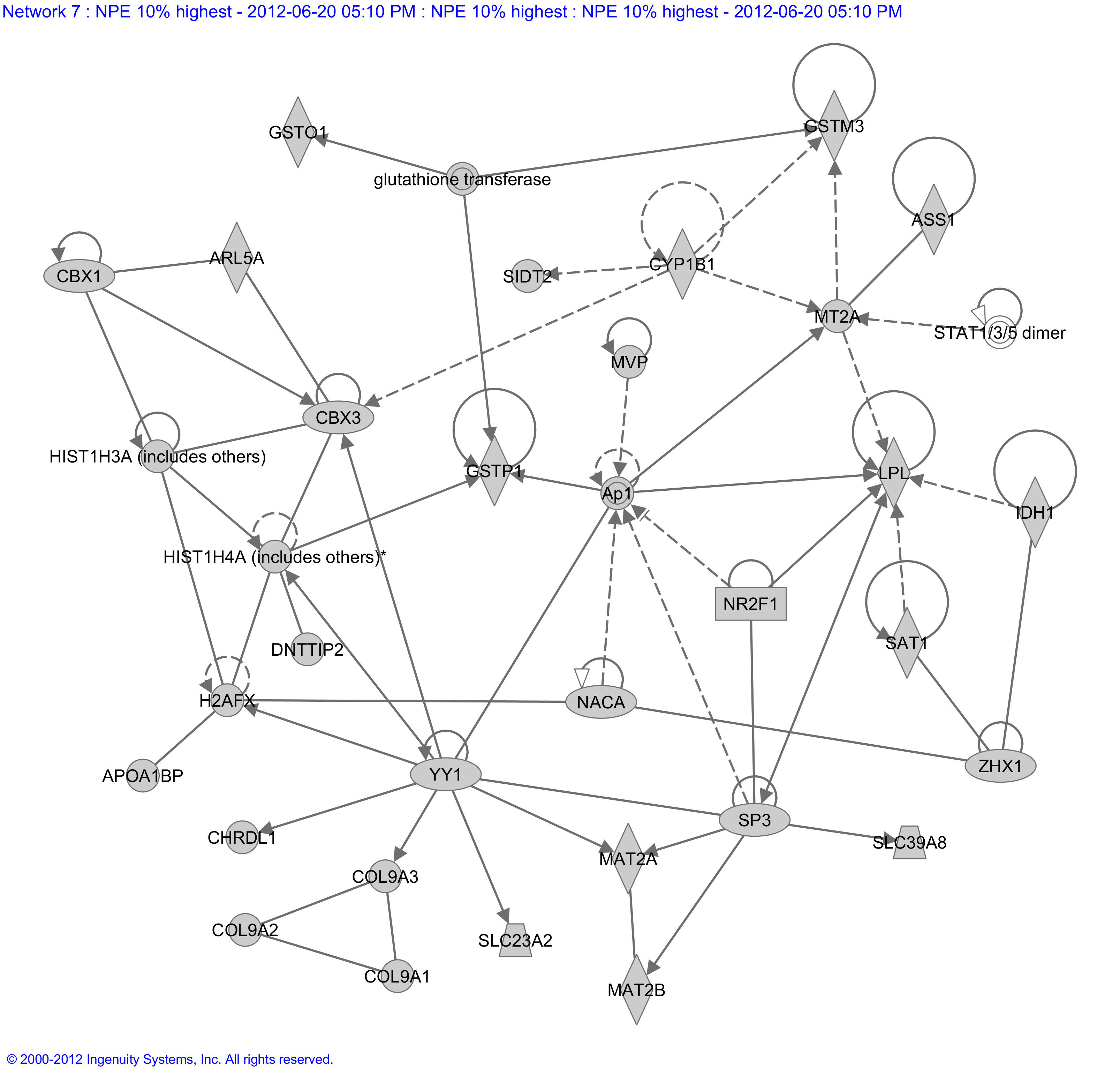

Supplement: Figure S19 — Molecular network generated by the Ingenuity software from the highest expressed genes of the NPE. Molecular network generated from our microarray data of the highest expressed genes of the non-pigmented epithelium (NPE). For explanation of symbols on the diagrams see legend Figure S11. The main functionalities given by Ingenuity for this molecular network are ‘Lipid metabolism, small molecule biochemistry, carbohydrate metabolism’. In this network we found genes involved in metabolic pathways, like LPL and CYP1B1 (lipid metabolism), IDH1 (energy production), GSTM3, GSTP1, GSTO1 and MT2A (detoxification and drug metabolism), SLC23A2 (transport of vitamin C) and SLC39A8 (zinc transport). This network is an example of the metabolic functionalities that we found in the NPE. (JPG) [file pone.0044973.s019.jpg]

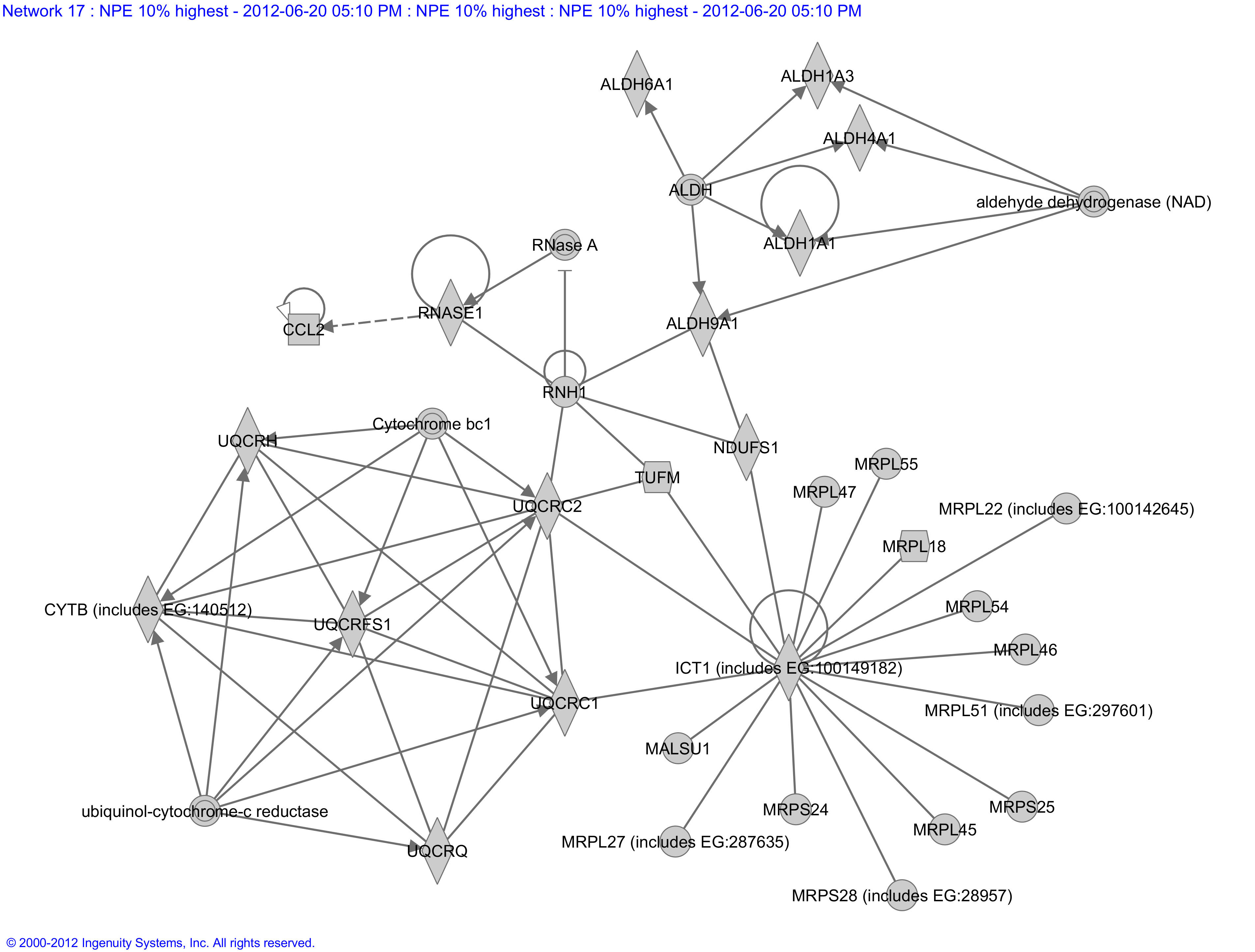

Supplement: Figure S20 — Molecular network generated by the Ingenuity software from the highest expressed genes of the NPE. Molecular network generated from our microarray data of the highest expressed genes of the non-pigmented epithelium (NPE). For explanation of symbols on the diagrams see legend Figure S11. The main functionalities given by Ingenuity for this molecular network are ‘Embryonic development, tissue morphology, organ development’. This network contained several subtypes of aldehyde dehydrogenase (ALDH1A1, ALDH1A3, ALDH4A1, ALDH6A1 and ALDH9A1) that are involved in alcohol, retinol and lipid metabolism. Therefore, this network is an example of the metabolic functionalities that we found in the NPE. (JPG) [file pone.0044973.s020.jpg]

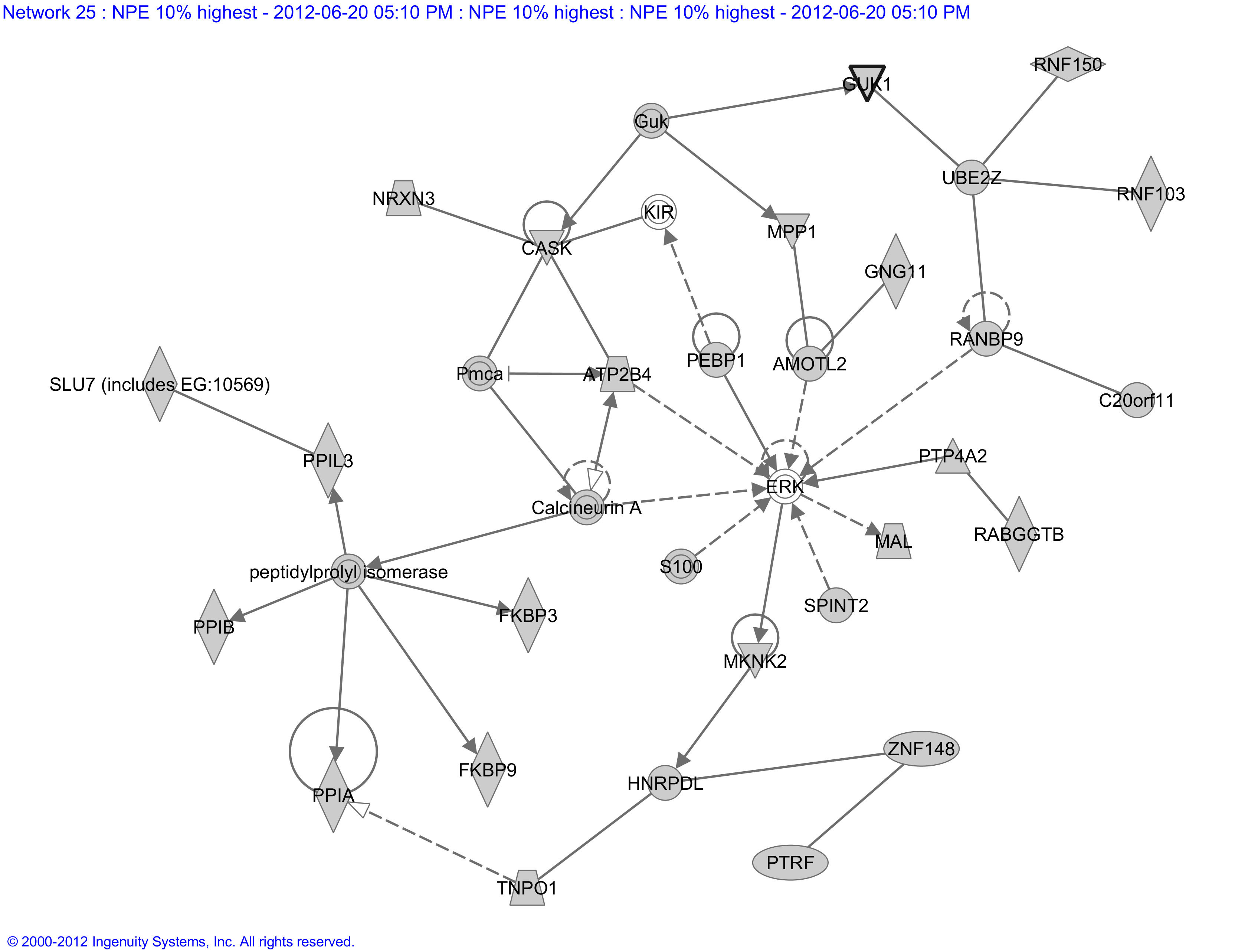

Supplement: Figure S21 — Molecular network generated by the Ingenuity software from the highest expressed genes of the NPE. Molecular network generated from our microarray data of the highest expressed genes of the non-pigmented epithelium (NPE). For explanation of symbols on the diagrams see legend Figure S11. The main functionalities given by Ingenuity for this molecular network are ‘Cell death, drug metabolism, small molecule biochemistry’. This network contained several genes which protein products are involved in immunosuppressive process and HIV infection, namely PPIA, PPIB, FKBP3 and FKBP9. Therefore, this network is an example for the immunological properties of the NPE. (JPG) [file pone.0044973.s021.jpg]

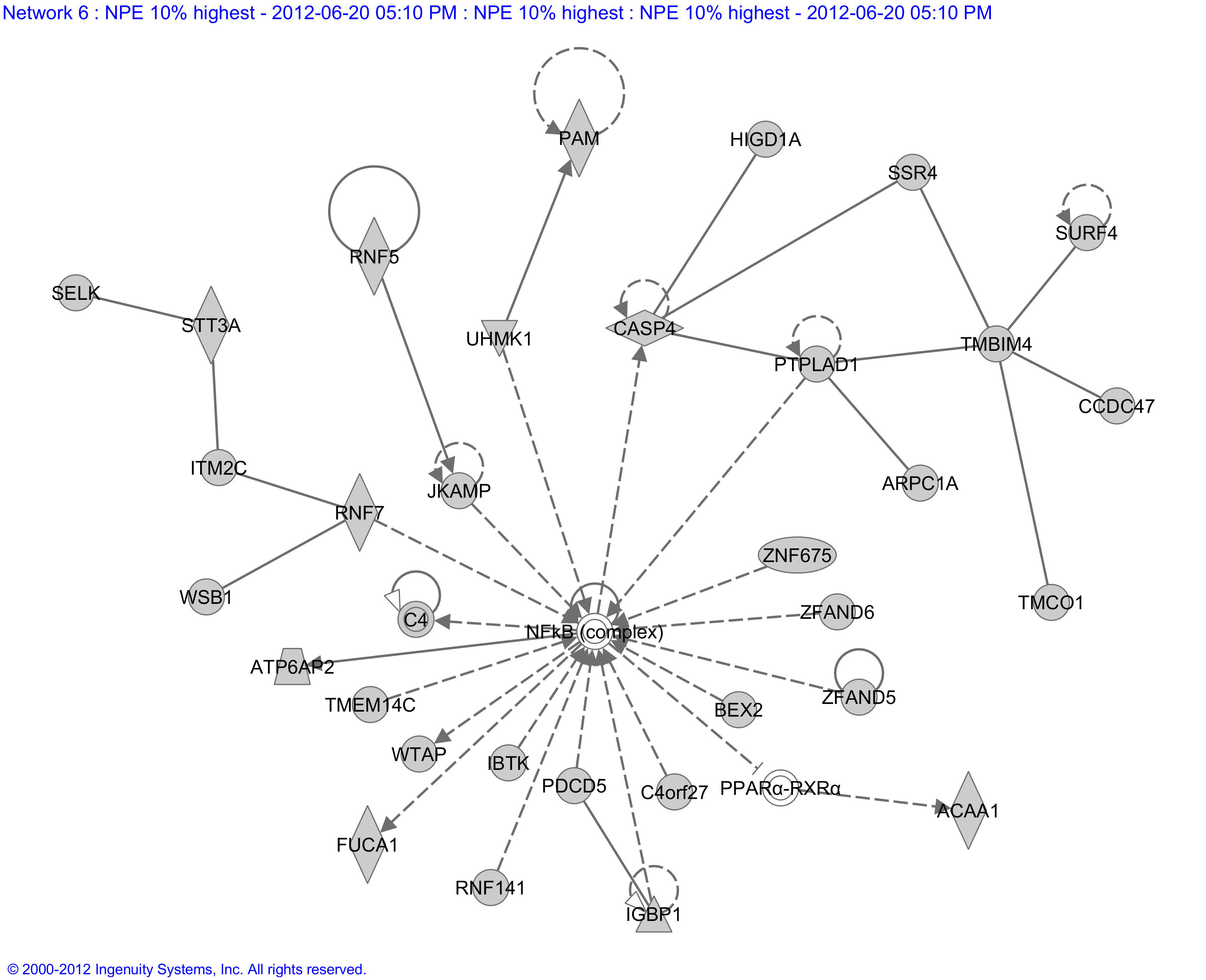

Supplement: Figure S22 — Molecular network generated by the Ingenuity software from the highest expressed genes of the NPE. Molecular network generated from our microarray data of the highest expressed genes of the non-pigmented epithelium (NPE). For explanation of symbols on the diagrams see legend Figure S11. The main functionalities given by Ingenuity for this molecular network are ‘Developmental disorder, hereditary disorder, neurological disease’. This network contained the gene TMCO1, which have been previously associated with POAG. (JPG) [file pone.0044973.s022.jpg]

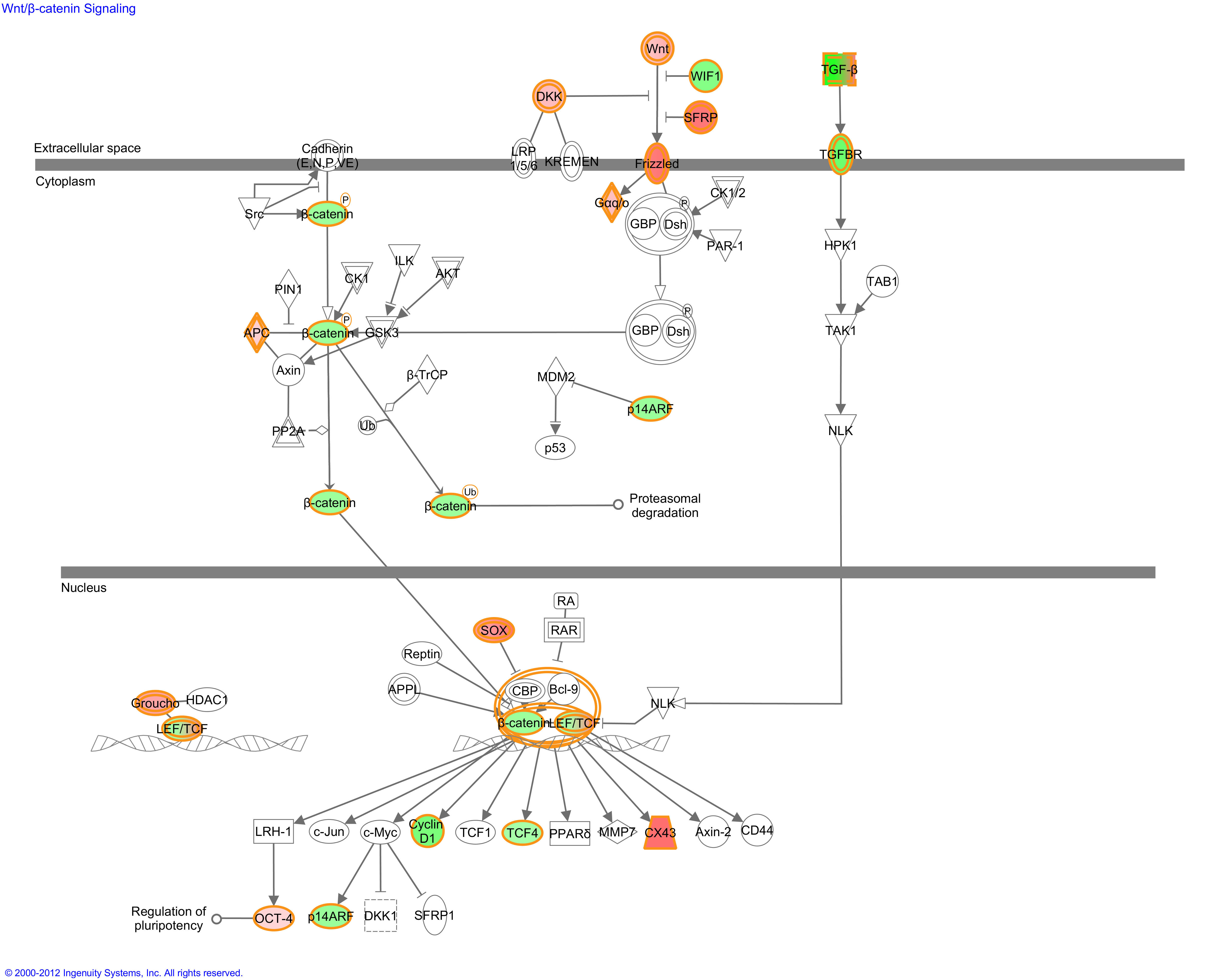

Supplement: Figure S23 — Wnt/β-catenin signaling pathway identified by the Ingenuity software. This is one of the canonical pathways that contains statistically significantly more genes than expected by chance in the group of genes that differ statistically significantly between non-pigmented (NPE) and pigmented epithelium (PE). Red fields indicate genes statistically significantly higher expressed in PE compared to NPE whereas green fields indicate genes statistically significantly higher expressed in NPE compared to PE. Fields with both green and red color represent gene groups, of which some genes are significantly higher expressed in NPE (green) and other in PE (red). Uncolored genes are added by the software to form the pathway. Solid lines between molecules indicate direct physical relationships between molecules (such as regulating and interacting protein domains); dotted lines indicate indirect functional relationships (such as co-regulation of expression of both genes in cell lines). Abbreviations of gene names are according to standard abbreviations used in Genbank. (JPG) [file pone.0044973.s023.jpg]

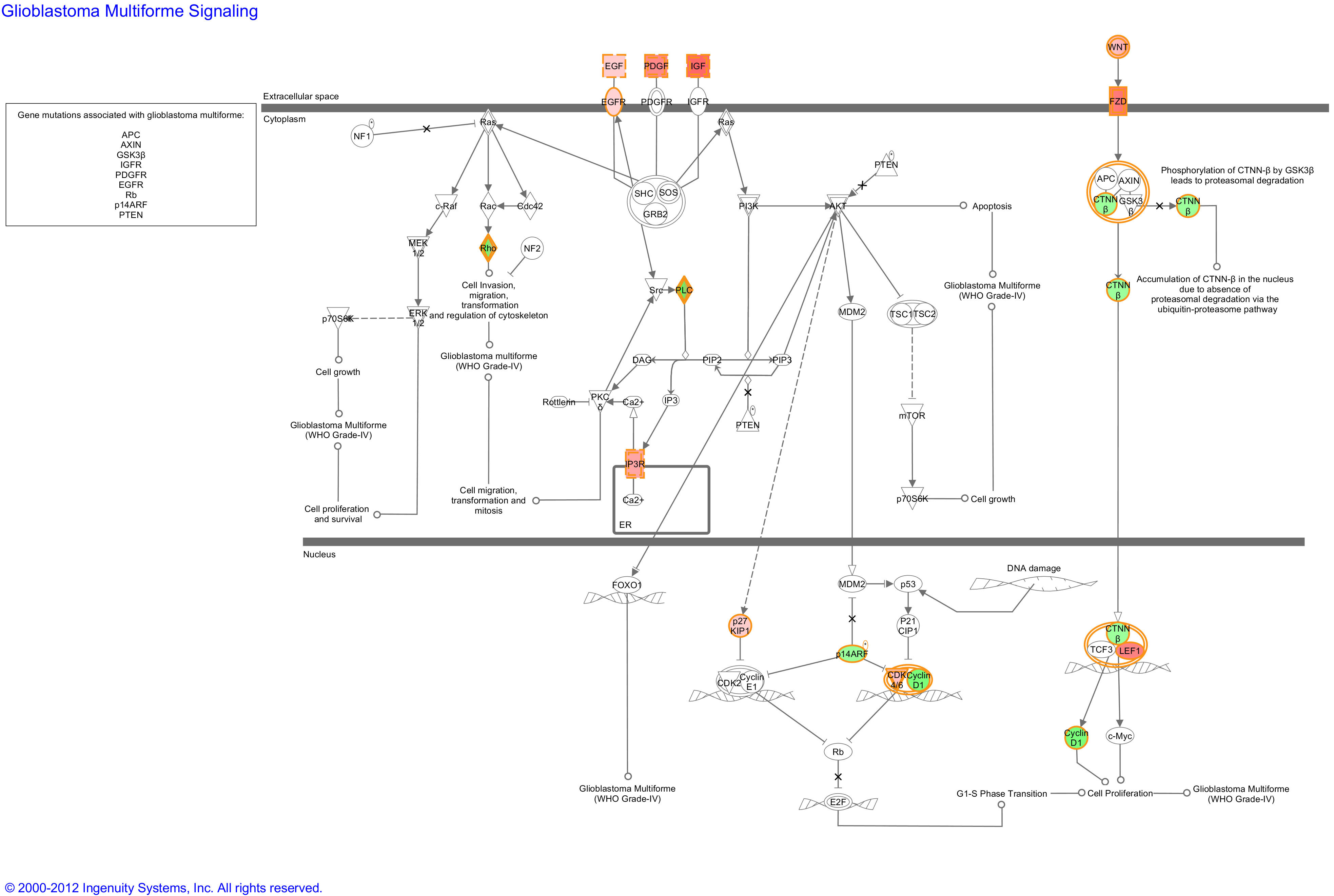

Supplement: Figure S24 — Glioblastoma multiforme signaling pathway identified by the Ingenuity software. This is one of the canonical pathways that contains statistically significantly more genes than expected by chance in the group of genes that differ statistically significantly between non-pigmented (NPE) and pigmented epithelium (PE). For explanation of symbols on the diagrams see legend Figure S23. (JPG) [file pone.0044973.s024.jpg]

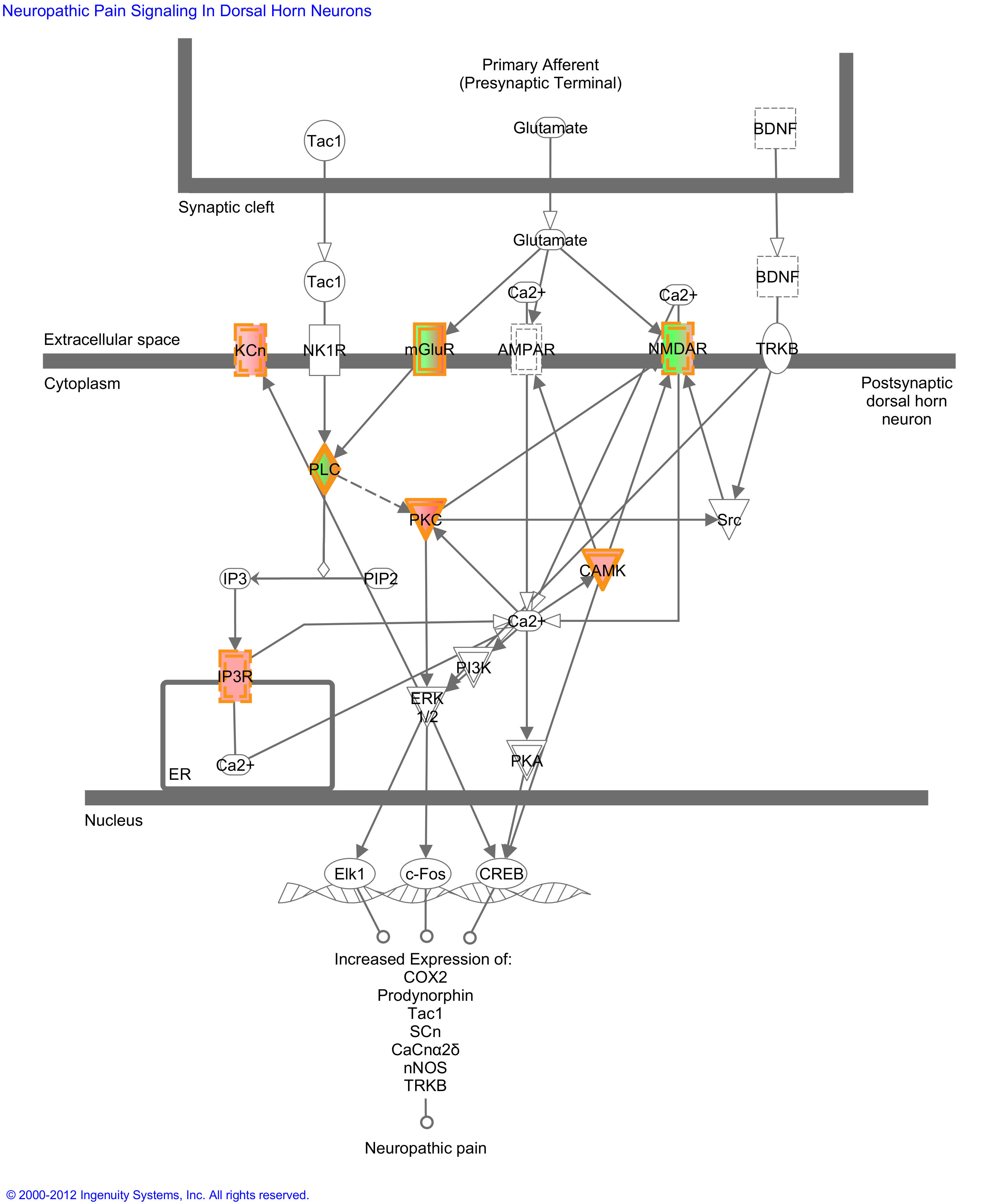

Supplement: Figure S25 — Neuropathic pain signaling in dorsal horn neurons pathway identified by the Ingenuity software. This is one of the canonical pathways that contains statistically significantly more genes than expected by chance in the group of genes that differ statistically significantly between non-pigmented (NPE) and pigmented epithelium (PE). For explanation of symbols on the diagrams see legend Figure S23. (JPG) [file pone.0044973.s025.jpg]

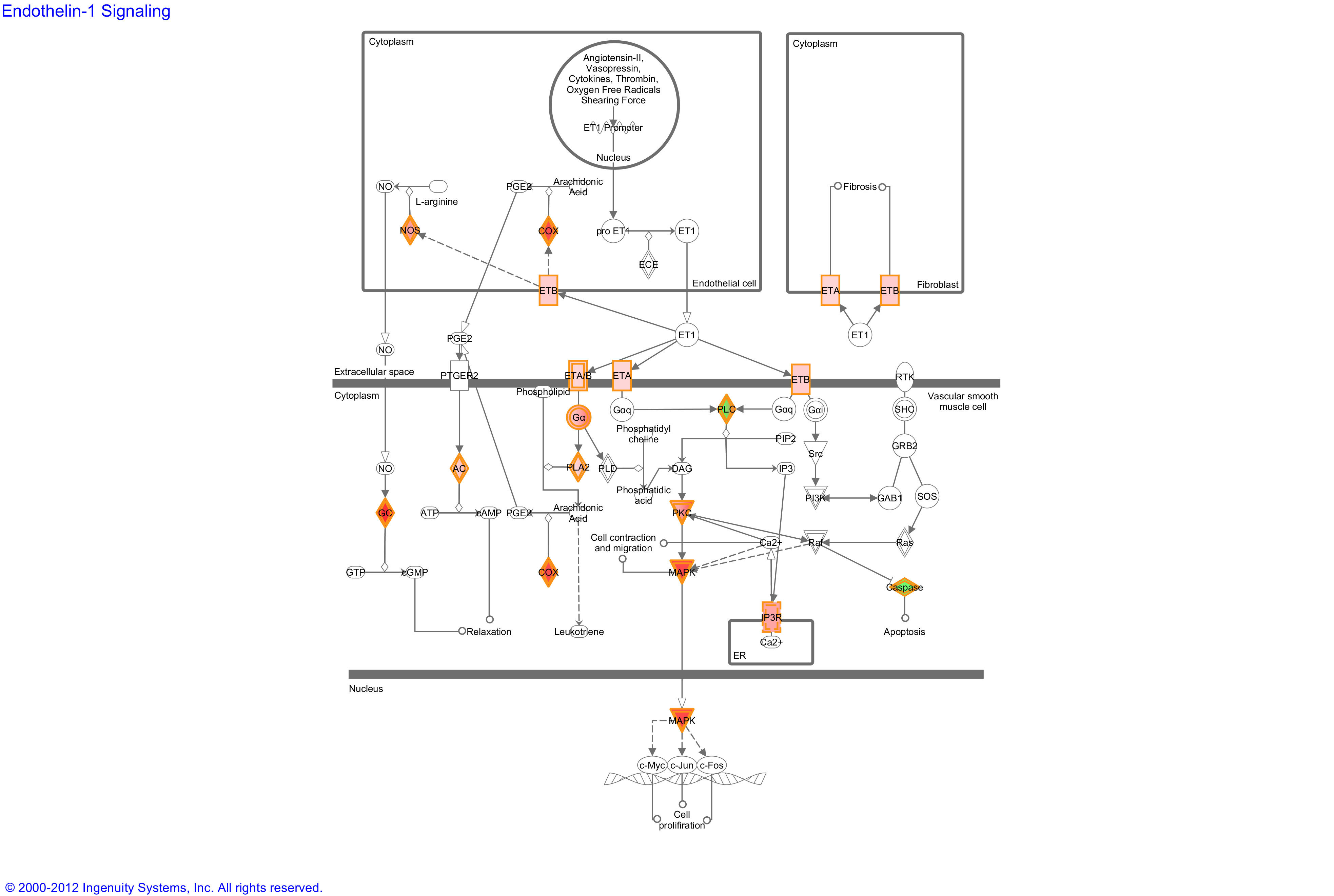

Supplement: Figure S26 — Endothelin-1 signaling pathway identified by the Ingenuity software. This is one of the canonical pathways that contains statistically significantly more genes than expected by chance in the group of genes that differ statistically significantly between non-pigmented (NPE) and pigmented epithelium (PE). For explanation of symbols on the diagrams see legend Figure S23. (JPG) [file pone.0044973.s026.jpg]

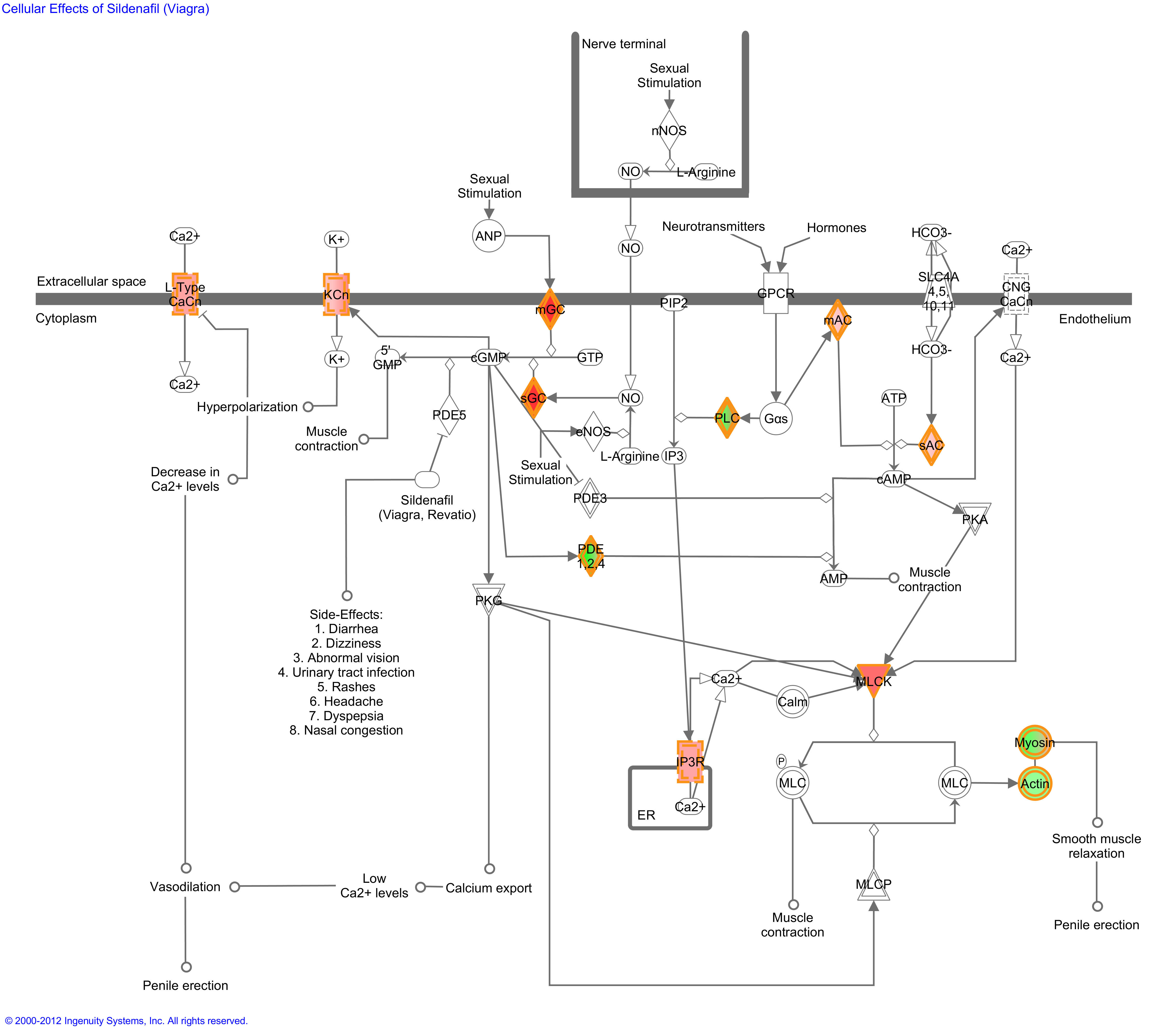

Supplement: Figure S27 — Cellular effects of Sildenafil (Viagra) pathway identified by the Ingenuity software. This is one of the canonical pathways that contains statistically significantly more genes than expected by chance in the group of genes that differ statistically significantly between non-pigmented (NPE) and pigmented epithelium (PE). For explanation of symbols on the diagrams see legend Figure S23. (JPG) [file pone.0044973.s027.jpg]

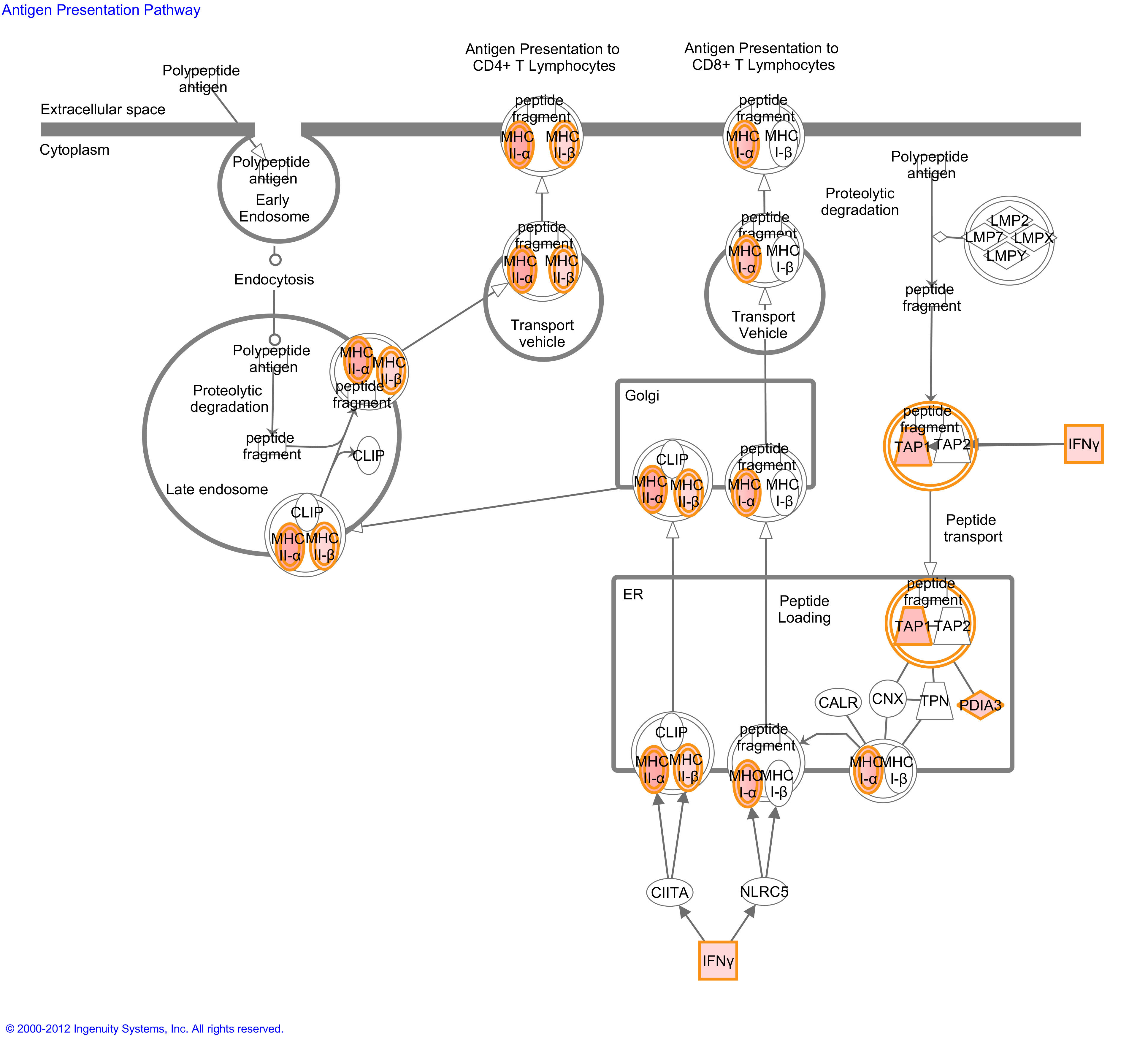

Supplement: Figure S28 — Antigen presentation pathway identified by the Ingenuity software. This is one of the canonical pathways that contains statistically significantly more genes than expected by chance in the group of genes that differ statistically significantly between non-pigmented (NPE) and pigmented epithelium (PE). For explanation of symbols on the diagrams see legend Figure S23. (JPG) [file pone.0044973.s028.jpg]

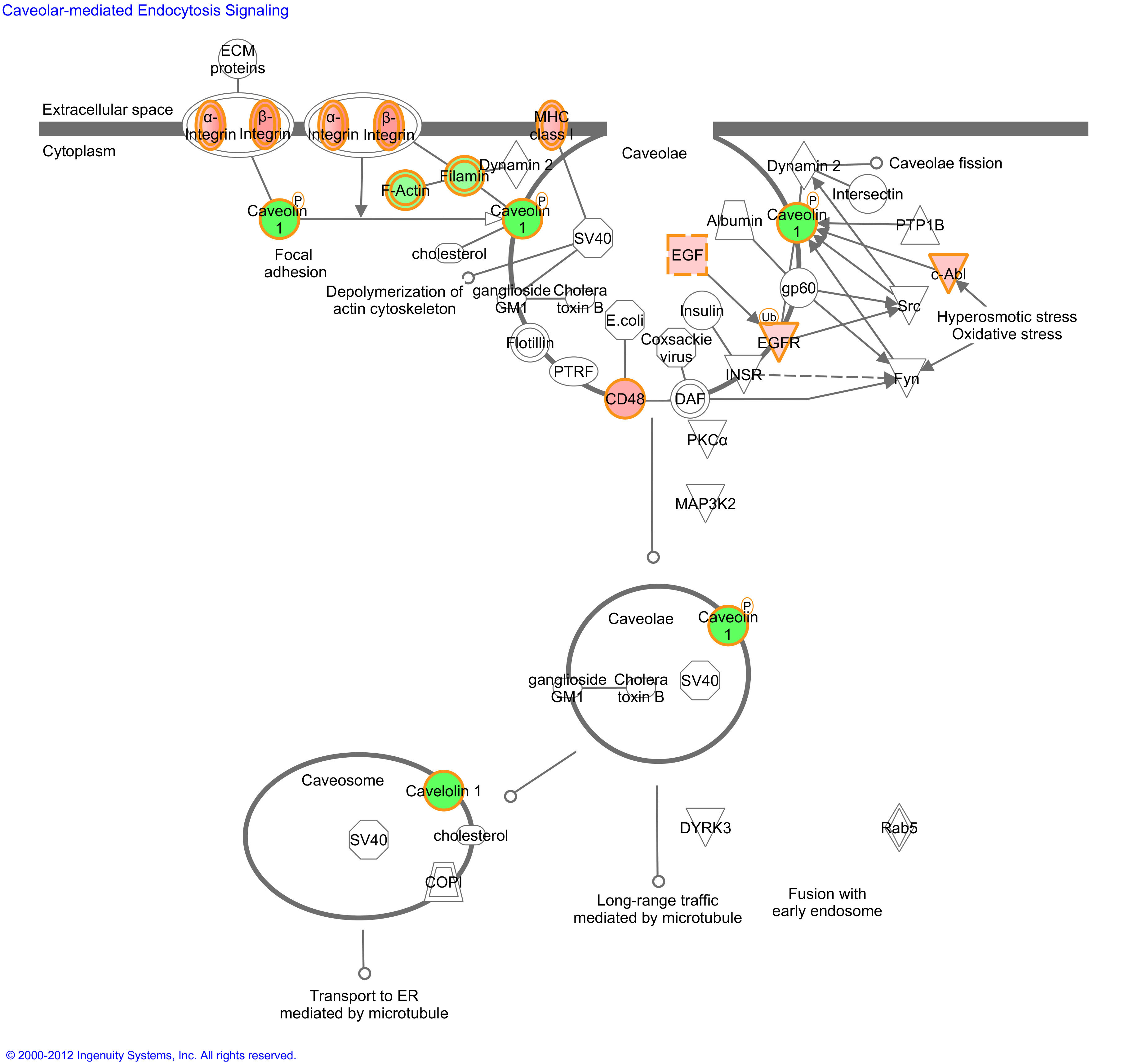

Supplement: Figure S29 — Caveolar-mediated endocytosis signaling pathway identified by the Ingenuity software. This is one of the canonical pathways that contains statistically significantly more genes than expected by chance in the group of genes that differ statistically significantly between non-pigmented (NPE) and pigmented epithelium (PE). For explanation of symbols on the diagrams see legend Figure S23. (JPG) [file pone.0044973.s029.jpg]

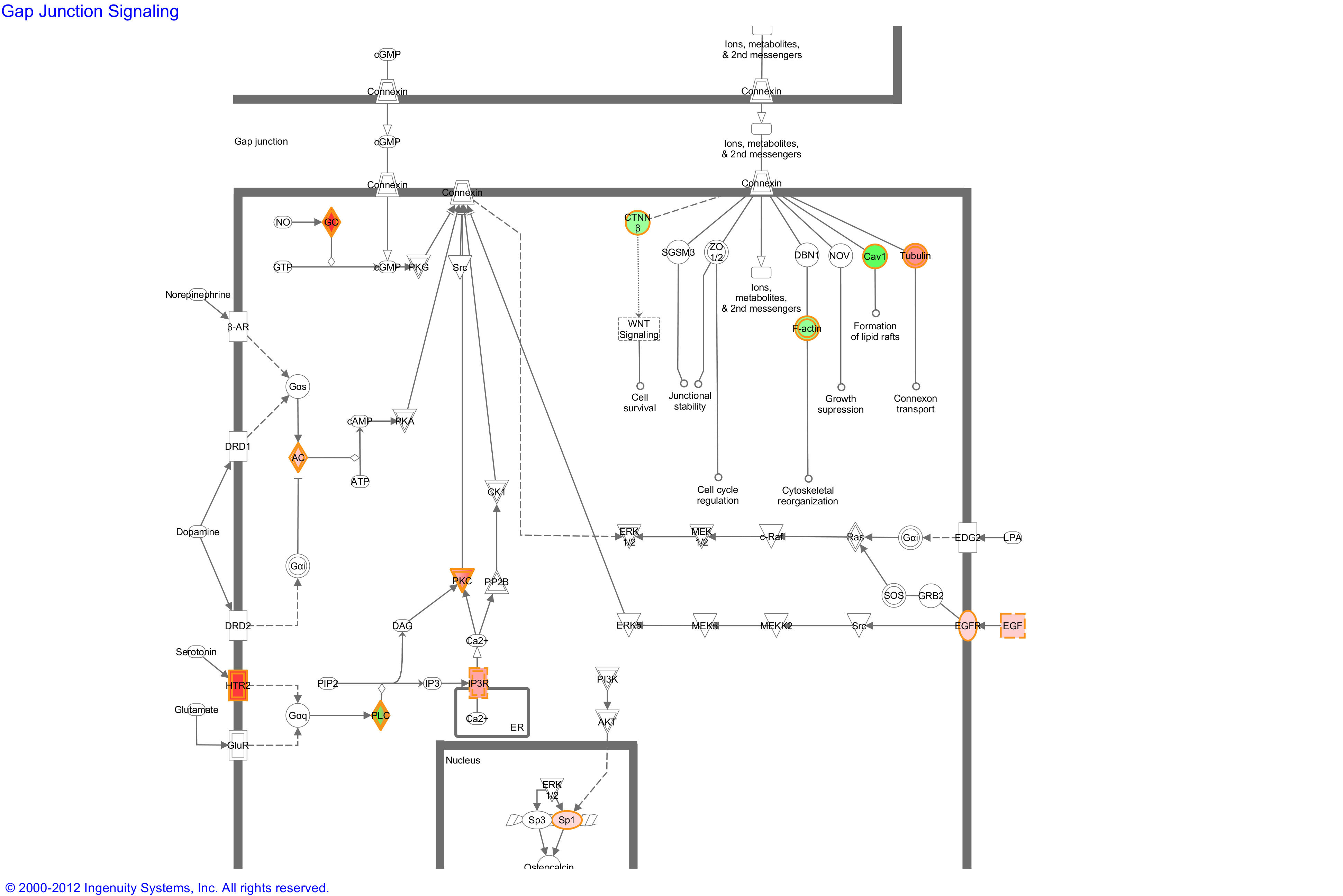

Supplement: Figure S30 — Gap junction signaling pathway identified by the Ingenuity software. This is one of the canonical pathways that contains statistically significantly more genes than expected by chance in the group of genes that differ statistically significantly between non-pigmented (NPE) and pigmented epithelium (PE). For explanation of symbols on the diagrams see legend Figure S23. (JPG) [file pone.0044973.s030.jpg]

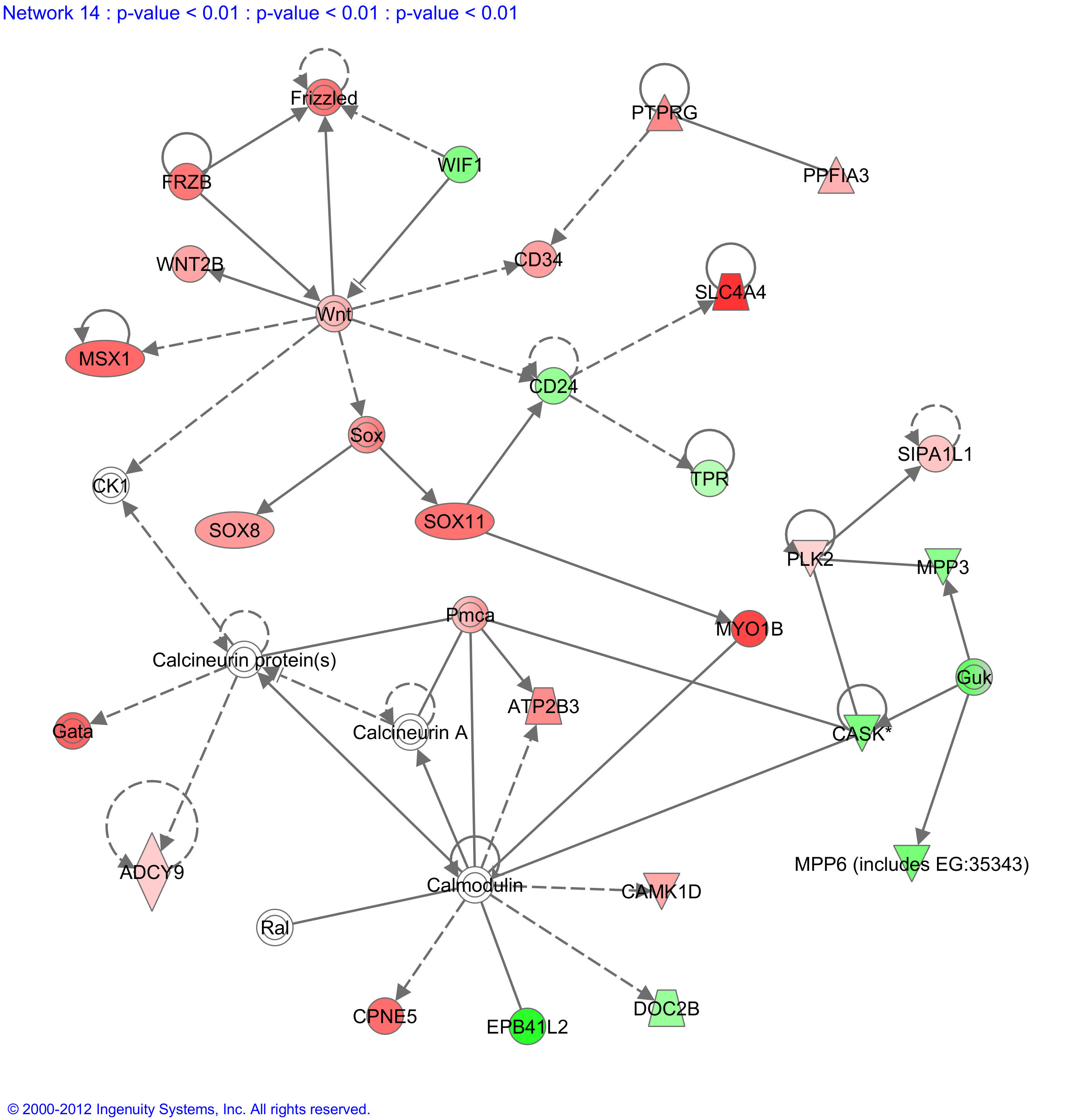

Supplement: Figure S31 — Molecular network generated by Ingenuity software from the statistically significantly different expressed genes. Molecular network generated from our microarray Significantly Different Expressed sub-dataset. Red fields indicate genes statistically significantly higher expressed in pigmented epithelium (PE) compared to non-pigmented epithelium (NPE) whereas green fields indicate genes statistically significantly higher expressed in NPE compared to PE. Transparent entries are molecules from the knowledge database, inserted to connect all relevant molecules in a single network. Solid lines between molecules indicate direct physical relationships between molecules (such as regulating and interacting protein domains); dotted lines indicate indirect functional relationships (such as co-regulation of expression of both genes in cell lines). Abbreviations of gene names are according to standard abbreviations used in Genbank. The main functionalities given by Ingenuity for this molecular network are ‘Connective tissue development and function, embryonic development, organ development’. This network contained several genes involved in embryonic development, namely genes SOX8, SOX11, MSX1, FRZB and WNT2B. (JPG) [file pone.0044973.s031.jpg]

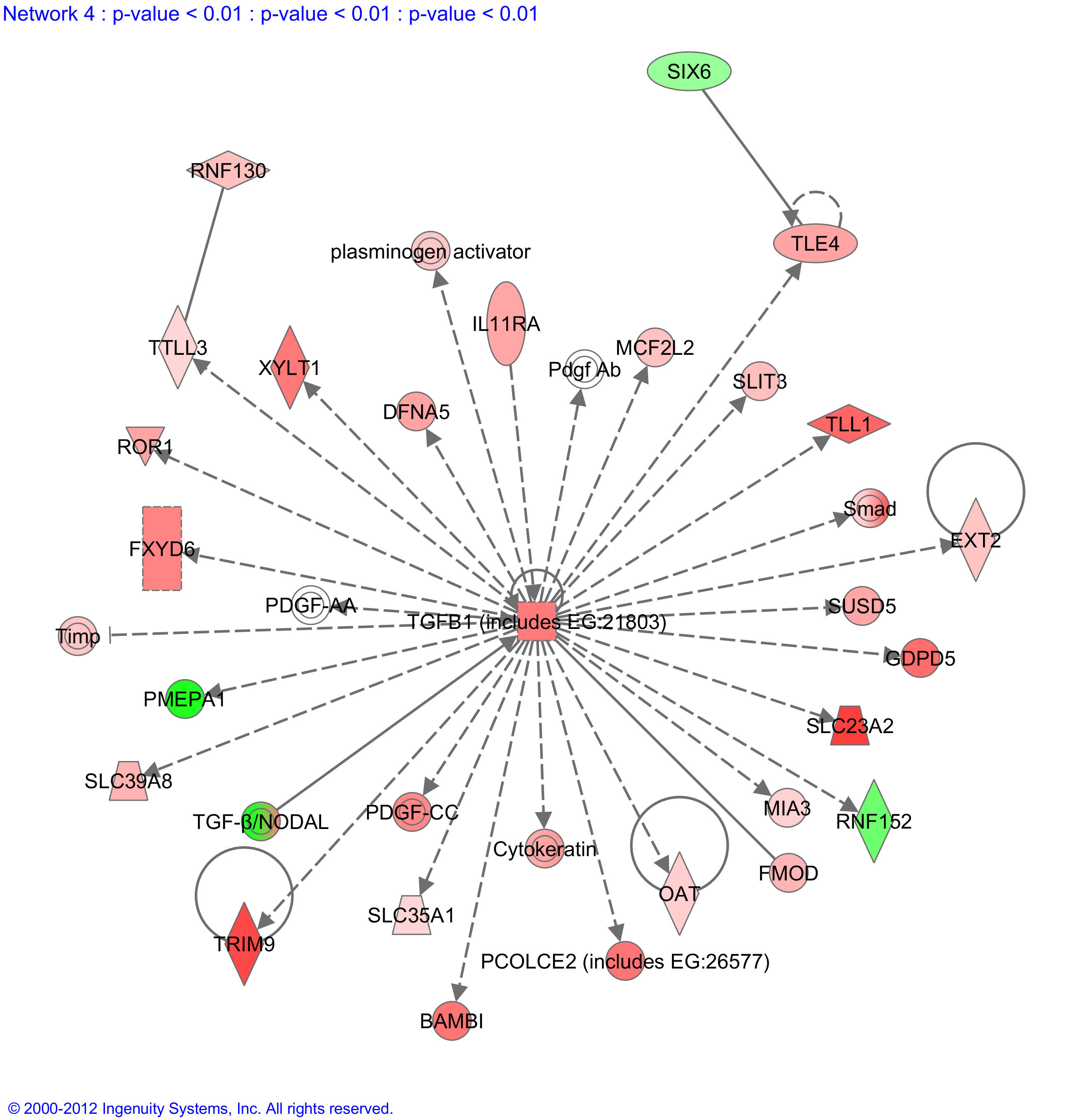

Supplement: Figure S32 — Molecular network generated by Ingenuity software from the statistically significantly different expressed genes. Molecular network generated from our microarray Significantly Different Expressed sub-dataset. For explanation of symbols on the diagrams see legend Figure S31. The main functionalities given by Ingenuity for this molecular network are ‘Carbohydrate metabolism, molecular transport, small molecule biochemistry’. Of interest, this network contained the gene SIX6, whose protein product is involved in eye development. (JPG) [file pone.0044973.s032.jpg]

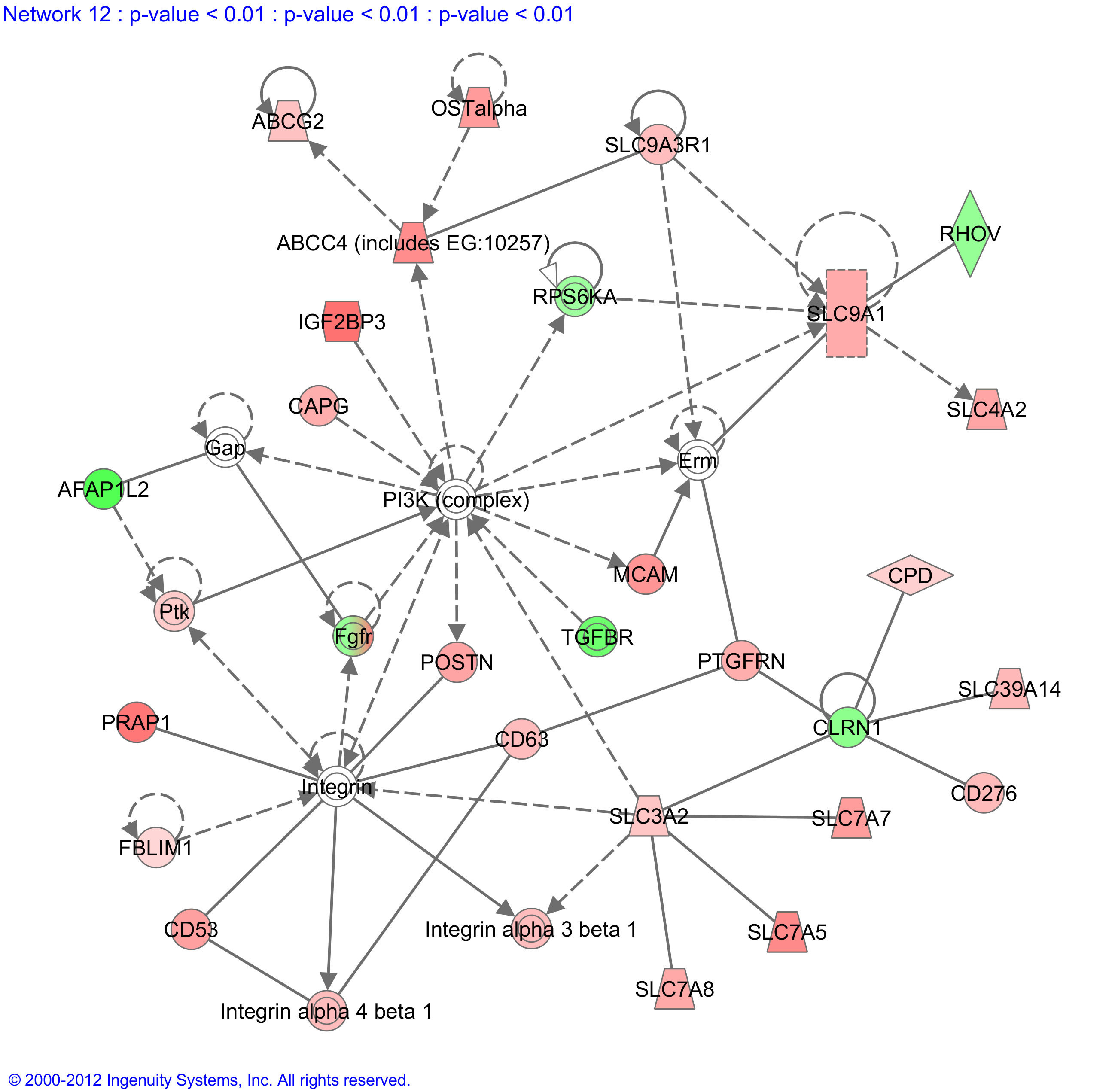

Supplement: Figure S33 — Molecular network generated by Ingenuity software from the statistically significantly different expressed genes. Molecular network generated from our microarray Significantly Different Expressed sub-dataset. For explanation of symbols on the diagrams see legend Figure S31. The main functionalities given by Ingenuity for this molecular network are ‘Amino acid metabolism, molecular transport, small molecule biochemistry’. Of interest, this network contained the gene CLRN1, whose protein product is involved in the development of retina. (JPG) [file pone.0044973.s033.jpg]

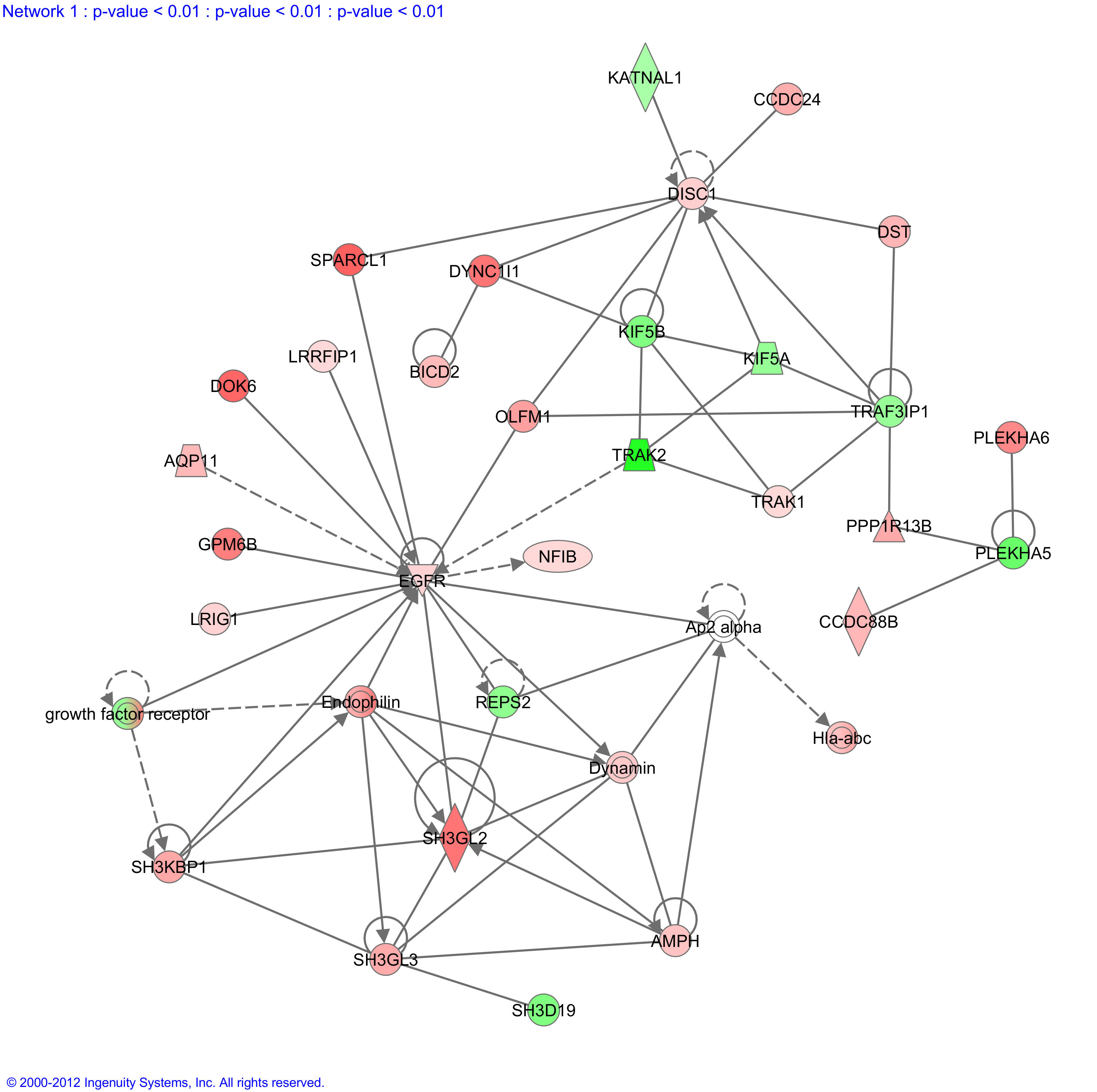

Supplement: Figure S34 — Molecular network generated by Ingenuity software from the statistically significantly different expressed genes. Molecular network generated from our microarray Significantly Different Expressed sub-dataset. For explanation of symbols on the diagrams see legend Figure S31. The main functionalities given by Ingenuity for this molecular network are ‘Cellular assembly and organization, cellular function and maintenance, cell death’. In this network we found centrally the gene DISC1. The protein of this gene is involved in neurite outgrowth and cortical development. Also, this gene is associated with schizophrenia. The gene DST is involved in transport of axons and coalignment of neurofilaments and the gene OLFM1 is neural tissue specific gene. (JPG) [file pone.0044973.s034.jpg]

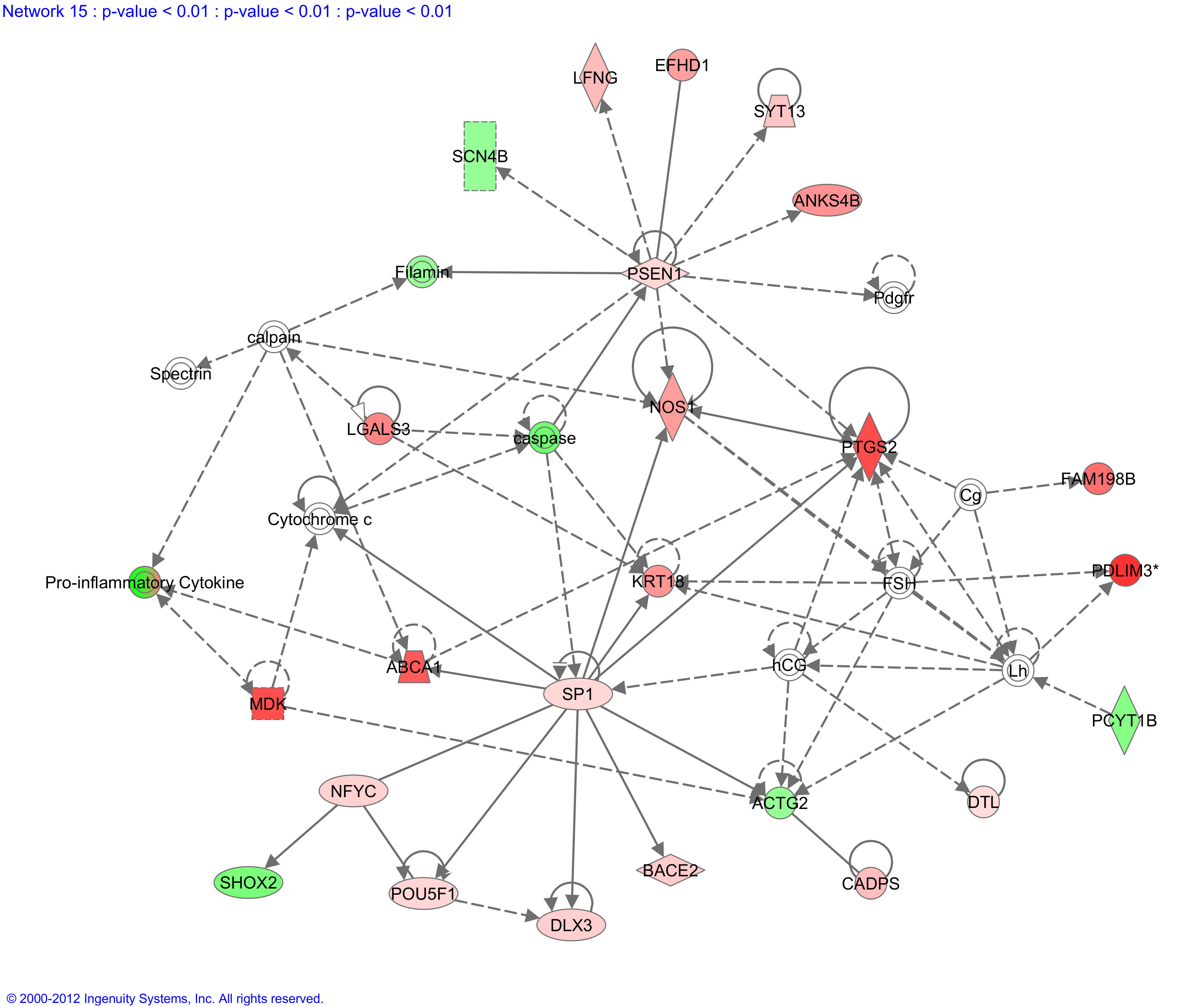

Supplement: Figure S35 — Molecular network generated by Ingenuity software from the statistically significantly different expressed genes. Molecular network generated from our microarray Significantly Different Expressed sub-dataset. For explanation of symbols on the diagrams see legend Figure S31. The main functionalities given by Ingenuity for this molecular network are ‘Connective tissue development and function, embryonic development, nervous system development and function’. In this network we found two genes that have been associated with neurodegeneration, namely PTGS2 and PSEN1. The protein products of PSEN1 and ABCA1 are involved in amyloid-beta metabolism. Moreover, the genes NOS1 and PTGS2 have been associated with glaucoma. (JPG) [file pone.0044973.s035.jpg]

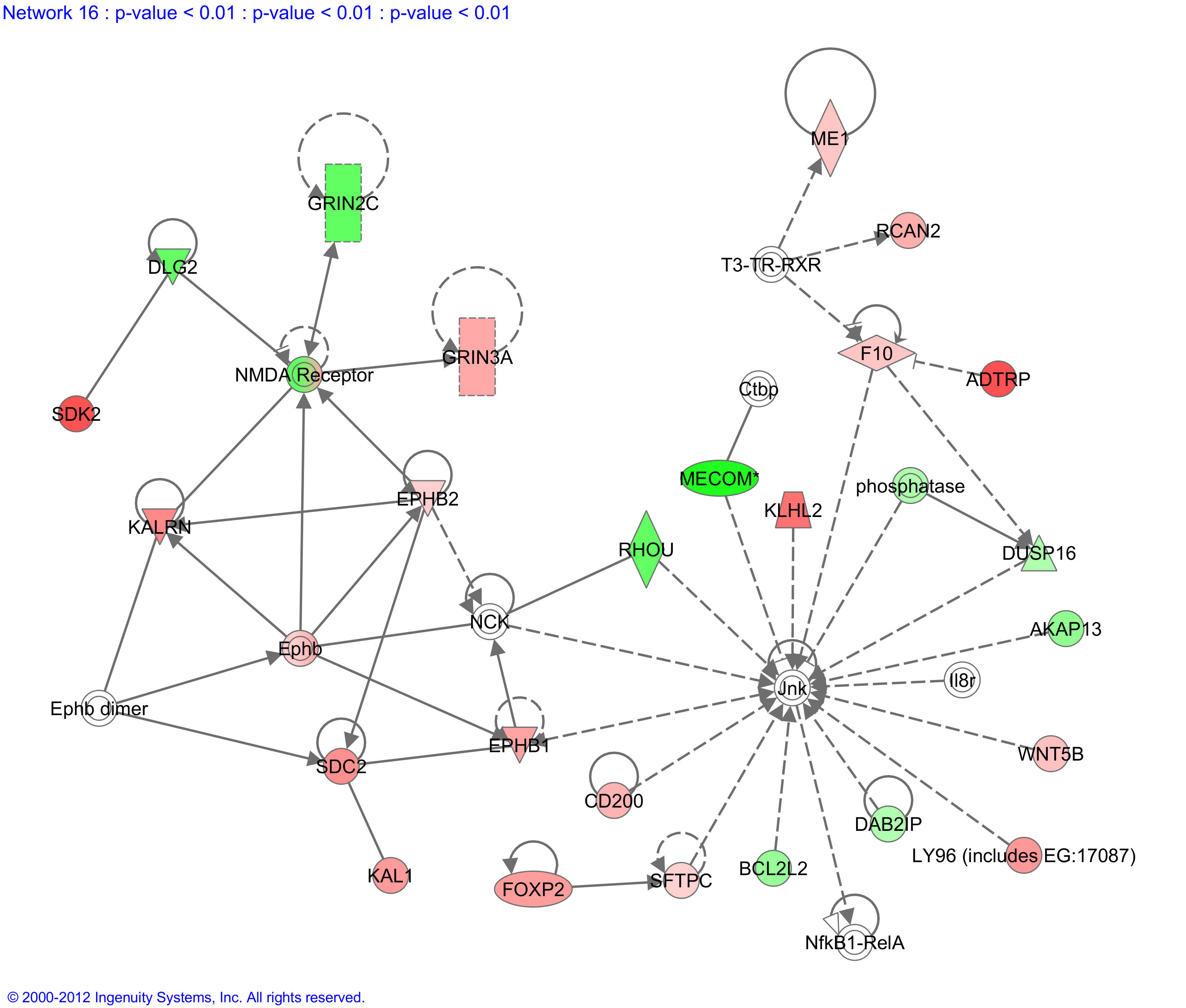

Supplement: Figure S36 — Molecular network generated by Ingenuity software from the statistically significantly different expressed genes. Molecular network generated from our microarray Significantly Different Expressed sub-dataset. For explanation of symbols on the diagrams see legend Figure S31. The main functionalities given by Ingenuity for this molecular network are ‘Cell morphology, cellular assembly and organization, cellular development’. This network contained the genes GRIN2C and GRIN3A, both coding for NMDA glutamate receptors that are important for neural signaling, and the genes EPH1, EPH2 and FOXP2, that are involved in morphogenesis of the eye. (JPG) [file pone.0044973.s036.jpg]

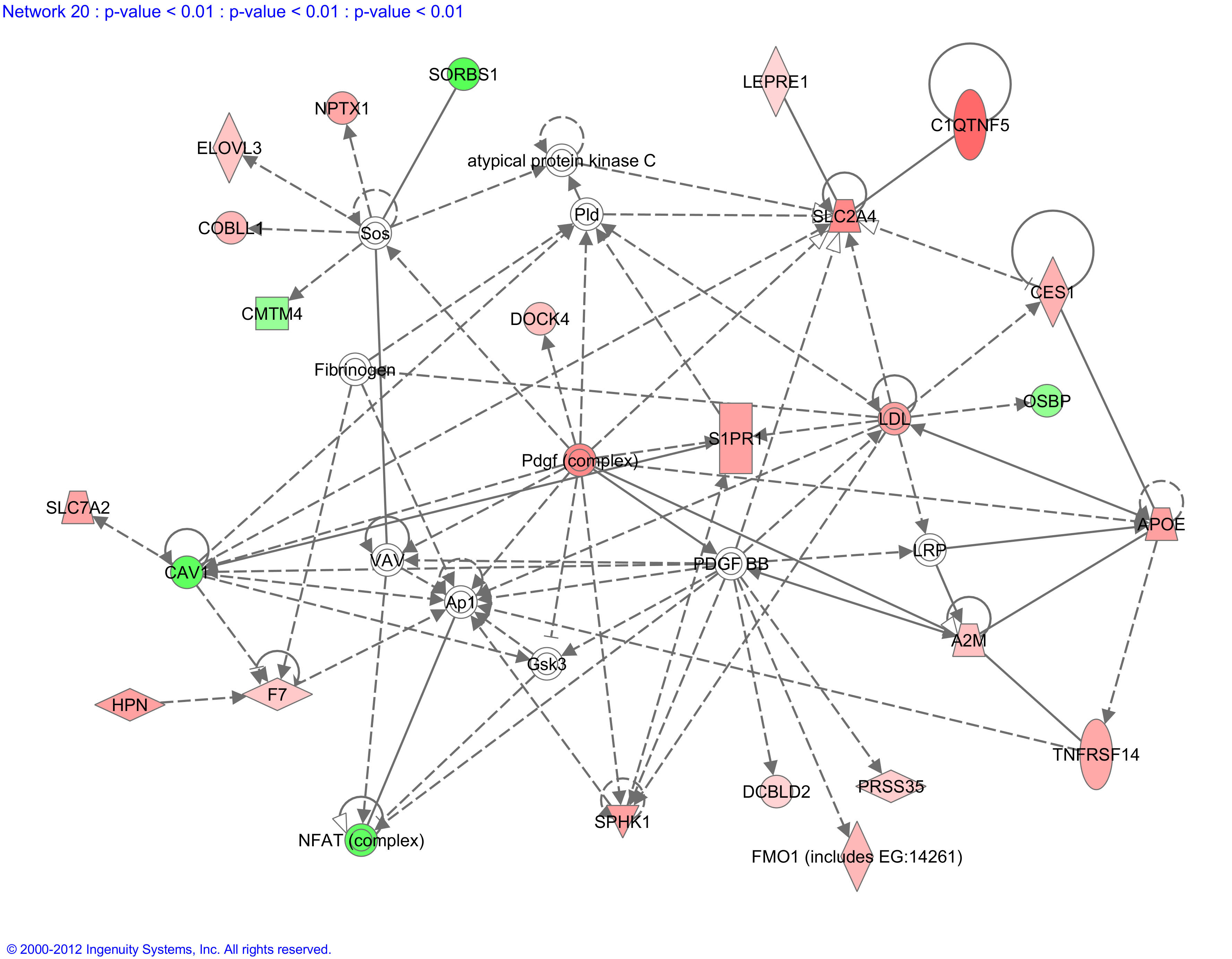

Supplement: Figure S37 — Molecular network generated by Ingenuity software from the statistically significantly different expressed genes. Molecular network generated from our microarray Significantly Different Expressed sub-dataset. For explanation of symbols on the diagrams see legend Figure S31. The main functionalities given by Ingenuity for this molecular network are ‘Lipid metabolism, molecular transport, small molecule biochemistry’. This network contained several molecules involved in lipid metabolism, namely APOE, LDL, OSBP and ELOVL3. Moreover, we found a gene in this network involved in drug metabolism (CES1) and glucose transport (SLC2A2). (JPG) [file pone.0044973.s037.jpg]

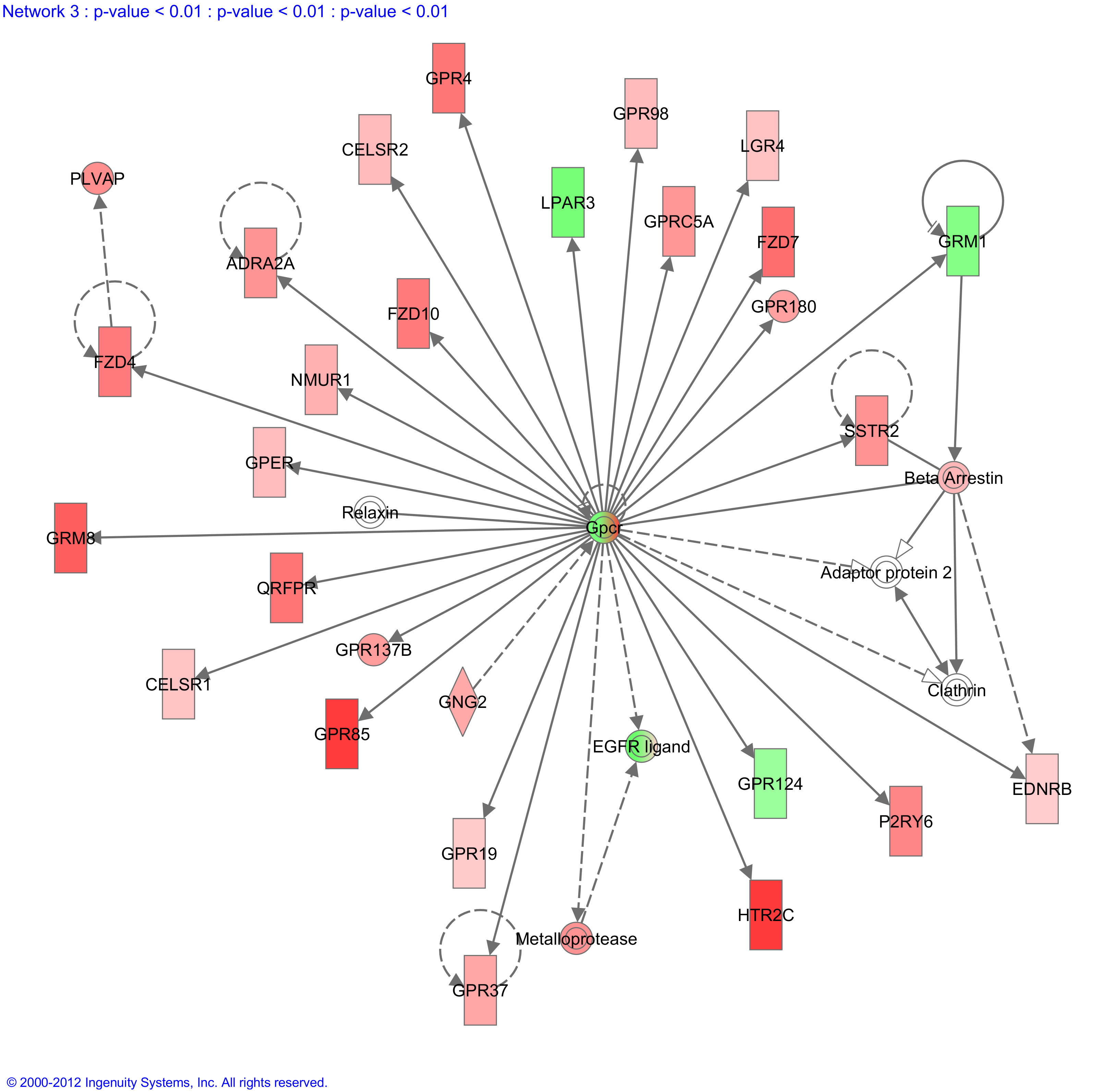

Supplement: Figure S38 — Molecular network generated by Ingenuity software from the statistically significantly different expressed genes. Molecular network generated from our microarray Significantly Different Expressed sub-dataset. For explanation of symbols on the diagrams see legend Figure S31. The main functionalities given by Ingenuity for this molecular network are ‘Carbohydrate metabolism, molecular transport, small molecule biochemistry’. This network was constructed with many G-coupled receptors that differed statistically significant between NPE and PE. G-coupled receptors are activated by extracellular stimuli, like neurotransmitters, hormones and light, and activate intracellular process. Apparently, some of these receptors differ between NPE and PE, for example GRM1 and GRM8 (glutamate receptor), ADRA2A (adrenergic alpha receptor), HTR2C (serotonin receptor), GPER (estrogen receptor) and EDNRB (endothelin B receptor). (JPG) [file pone.0044973.s038.jpg]

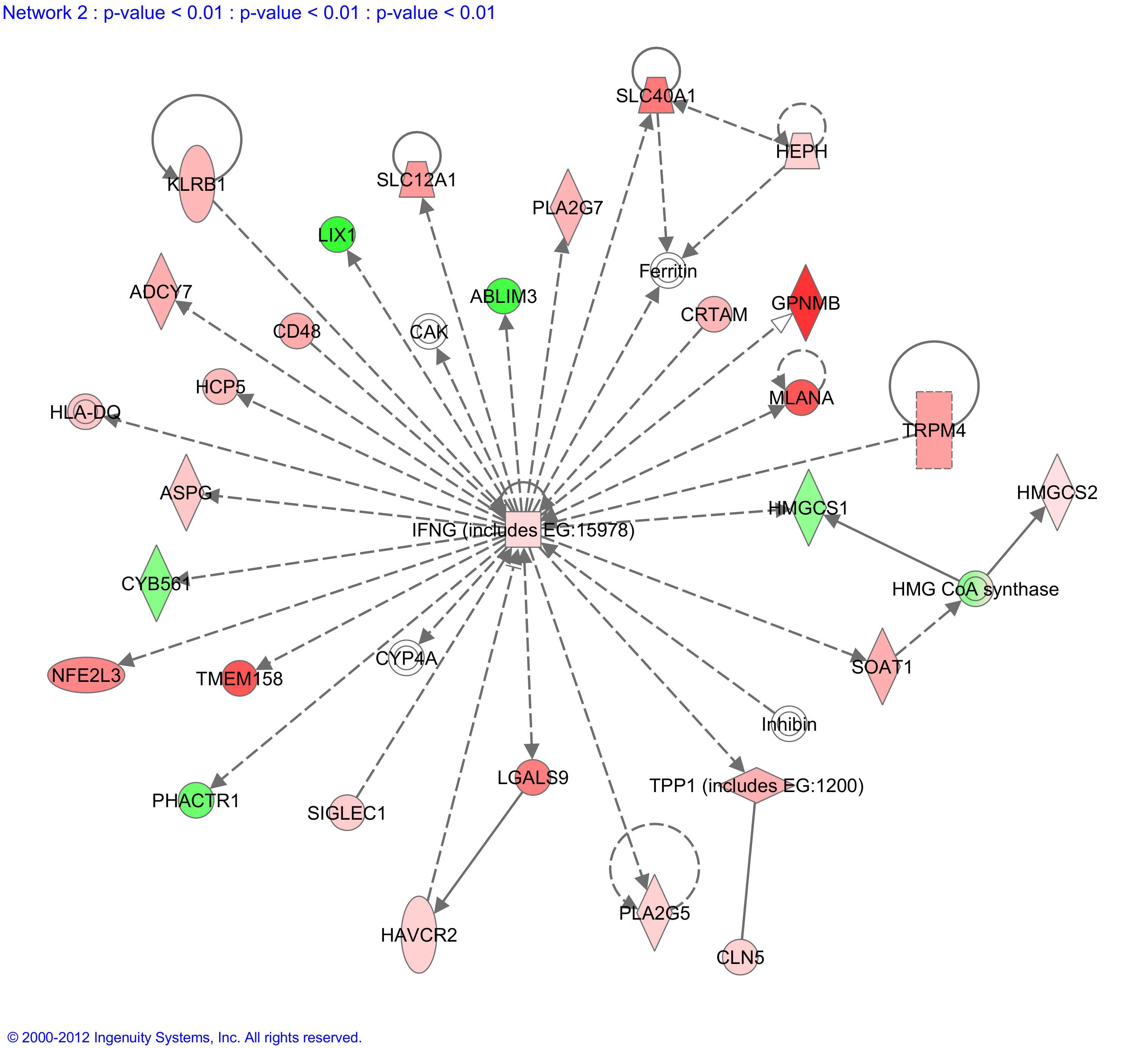

Supplement: Figure S39 — Molecular network generated by Ingenuity software from the statistically significantly different expressed genes. Molecular network generated from our microarray Significantly Different Expressed sub-dataset. For explanation of symbols on the diagrams see legend Figure S31. The main functionalities given by Ingenuity for this molecular network are ‘Cell-to-cell signaling and interaction, inflammatory response, carbohydrate metabolism’. In this network we found several genes whose protein products are involved in immunological process, including IFNG, HAVCR2 and KLRB1 (activation of macrophages and natural killer cells), CD48 and CRTAM (T-cell mediated immune response), HCP5 and HLA-DQ (HLA complex subtypes). (JPG) [file pone.0044973.s039.jpg]

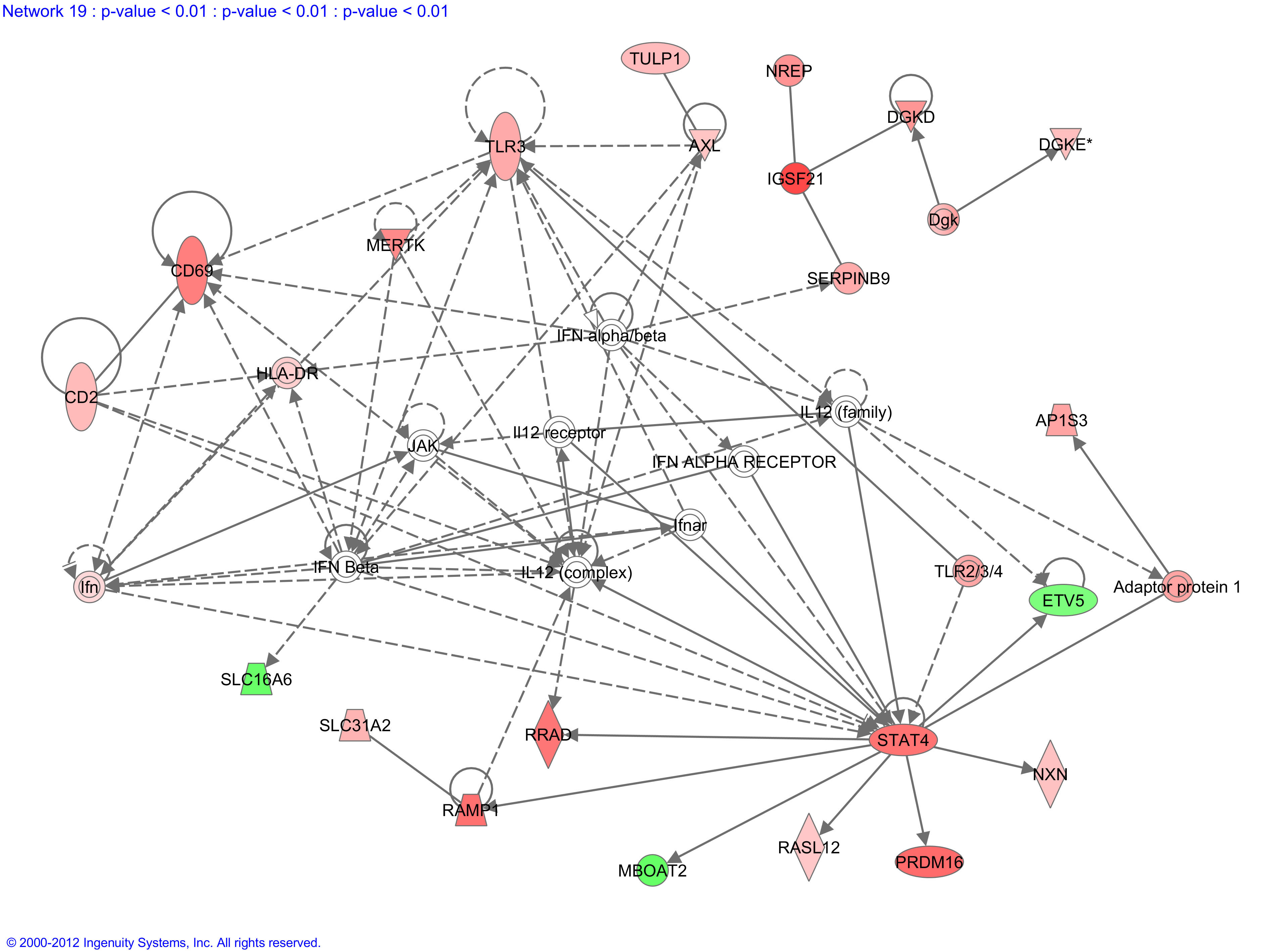

Supplement: Figure S40 — Molecular network generated by Ingenuity software from the statistically significantly different expressed genes. Molecular network generated from our microarray Significantly Different Expressed sub-dataset. For explanation of symbols on the diagrams see legend Figure S31. The main functionalities given by Ingenuity for this molecular network are ‘Cell-to-cell signaling and interaction, inflammatory response, cellular function and maintenance’. In this network we identified the genes STAT4 whose protein product mediates responses to IL12 lymphocytes and regulate the differentiation of T-helper cells. Moreover, this network contained the gene TLR3 (fundamental role in pathogen recognition and activation of innate immunity), IGSF21 (an immunoglobulin) and CD2 and CD69 (activation of T-lymphocytes). (JPG) [file pone.0044973.s040.jpg]

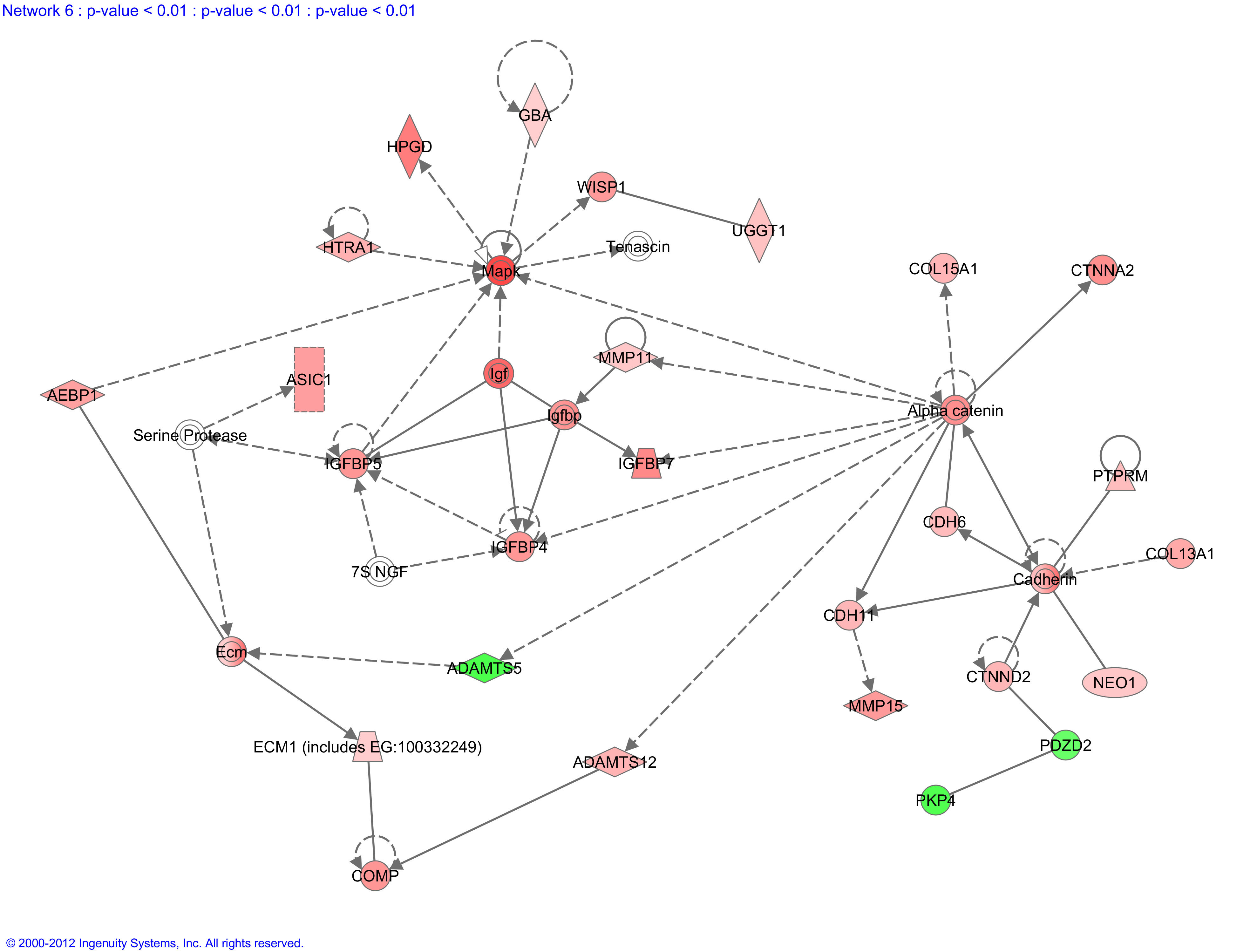

Supplement: Figure S41 — Molecular network generated by Ingenuity software from the statistically significantly different expressed genes. Molecular network generated from our microarray Significantly Different Expressed sub-dataset. For explanation of symbols on the diagrams see legend Figure S31. The main functionalities given by Ingenuity for this molecular network are ‘Connective tissue disorders, cellular movement, skeletal and muscular system development and function’. This network contained genes coding for cadherin (CDH6, CDH11), catenin (CTNNA2, CTNND2), collagen (COL13A1, COL15A1) and extracellular matrix protein (ECM1). (JPG) [file pone.0044973.s041.jpg]

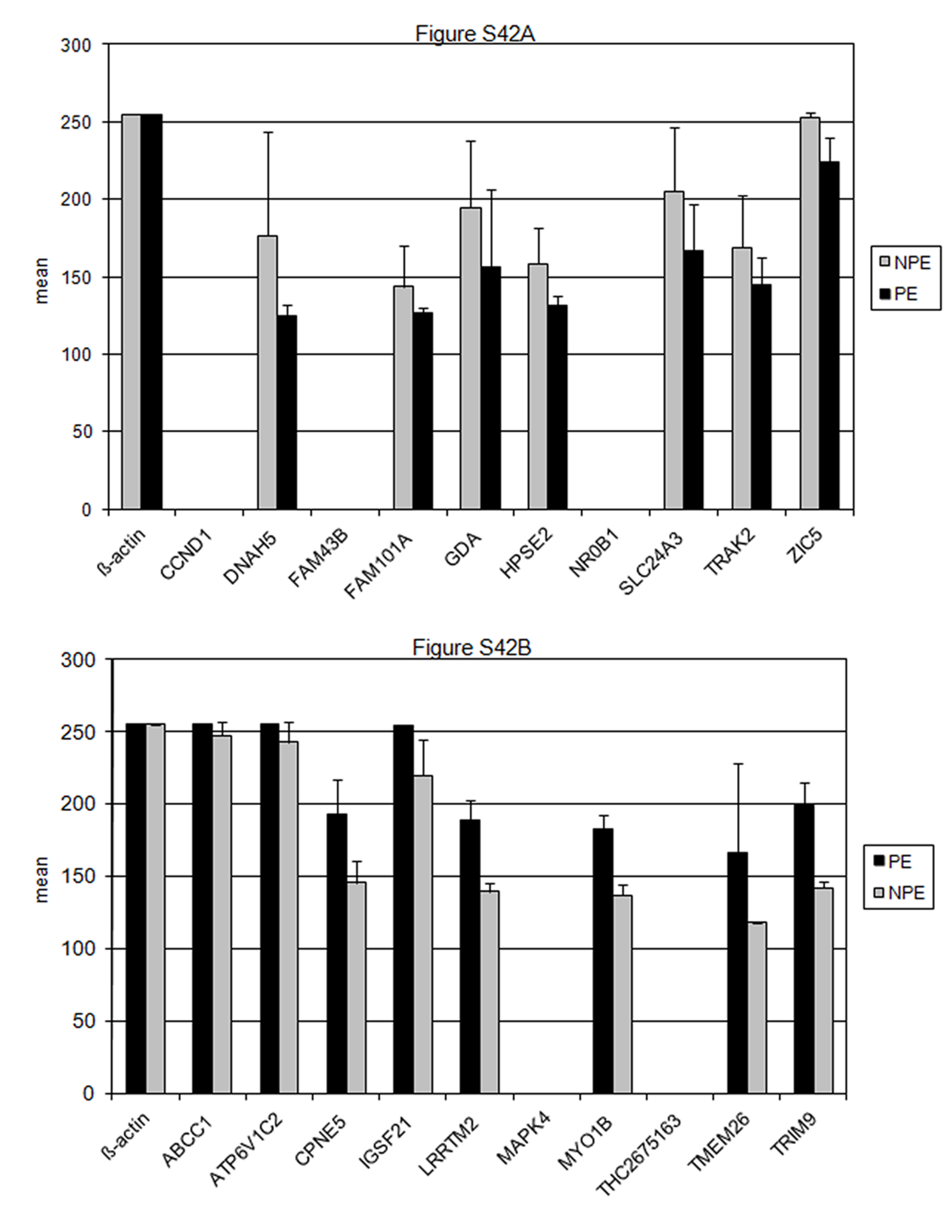

Supplement: Figure S42 — Confirmation of microarray results by s-QPCR. A: significantly different expressed genes with higher expression in NPE. Beta actin, a household gene, was used to normalize gene expression in the non-pigmented (NPE) and pigmented epithelium (PE) of the ciliary body. Seven genes that differed statistically significant between NPE and PE, with higher expression in NPE were confirmed by s-QPCR and outlined here. The grey bars indicate NPE expression and the black bars PE expression. Standard deviation is calculated. Three genes (CCND1, FAM43B, NR0B1) did not give any PCR products, even after repetitive attempts. B. Confirmation of microarray results by s-QPCR: significantly different expressed genes with higher expression in PE. Beta actin, a household gene, was used to normalize gene expression in the non-pigmented (NPE) and pigmented epithelium (PE) of the ciliary body. Eight genes that differed statistically significant between NPE and PE, with higher expression in PE were confirmed by s-QPCR and outlined here. The grey bars indicate NPE expression and the black bars PE expression. Standard deviation is calculated. Two genes (MAPK4, THC2675163) did not give any PCR products, even after repetitive attempts. (TIF) [file pone.0044973.s042.tif]
