# Supplementary material for: Gene Expression and Functional Annotation of the Human Ciliary Body Epithelia
Source: PLoS One. 2012 Sep 18;7(9):e44973. doi: 10.1371/journal.pone.0044973 (PMC3445623; doi:10.1371/journal.pone.0044973)
Supplement: Table S1 — Donor eye data. (DOC) [file pone.0044973.s043.doc]

**Table S1. Donor eye data**

|  | Age (y) | Gender | Cause of death | Medical history | Medication |
| --- | --- | --- | --- | --- | --- |
| 1. | 56 | M | Aortic dissection | None | None |
| 2. | 68 | M | Brain infarction | Hypertension | Carbasalaatcalcium, atorvastatine |
| 3. | 39 | M | Myocardial infarction | None | None |
| 4. | 73 | M | Myocardial infarction | None | None |
| 5. | 58 | M | Myocardial infarction | None | None |
| 6. | 48 | F | Myocardial infarction | None | None |
| 7. | 70 | F | Myocardial infarction | None | Unknown |

Abbreviations: y: years; M: male; F: Female
