# Supplementary material for: Gene Expression and Functional Annotation of the Human Ciliary Body Epithelia
Source: PLoS One. 2012 Sep 18;7(9):e44973. doi: 10.1371/journal.pone.0044973 (PMC3445623; doi:10.1371/journal.pone.0044973)
Supplement: Table S2 — Previously studied immune-localization of proteins in the ciliary body; A literature study. (DOC) [file pone.0044973.s044.doc]

**Table S2: Previously studied immune-localization of proteins in the ciliary body; A literature study**

| **LITERATURE** | | | | | **OUR DATA** |
| --- | --- | --- | --- | --- | --- |
| **Gene** | **NM-number** | **Author** | **Species** | **Localization** | **Intensity** |
|  |  |  |  |  | **NPE-PE** |
| ABCC2 | NM_000392 | Pelis et al.[115] | human+porcine | NPE+PE | L-L |
| ACTG1 | NM_001614 | Sonsino et al. [116] | human | NPE+PE | H-H |
| AQP1 | NM_198098 | Hasegawa et al [117] | human +rat | NPE+PE | H-H |
| ATP1A1 | NM_000701 | Wetzel and Sweadner [118] | mouse+rat | NPE+PE | H-H |
|  |  | Ghosh et al. [119] | bovine | NPE+PE |  |
| ATP1A2 | NM_000702 | Wetzel and Sweadner [118] | mouse+rat | NPE | H-H |
|  |  | Ghosh et al. [119] | bovine | NPE |  |
| ATP1A3 | NM_152296 | Ghosh et al. [119] | bovine | NPE | M-M |
|  |  | Wetzel and Sweadner [118] | mouse+rat | NPE+PE |  |
| ATP1B1 | NM_001677 | Wetzel and Sweadner [118] | mouse+rat | PE>NPE | H-H* |
| ATP1B2 | NM_001678 | Wetzel and Sweadner [118] | mouse+rat | NPE+PE | H-H |
| ATP1B3 | NM_001679 | Wetzel and Sweadner [118] | mouse+rat | NPE | H-H |
| BEST2 | NM_017682 | Zhang et al. [120] | human | NPE | H-H |
| CLU | NM_203339 | Zenkel et al. [121] | human | NPE+PE | H-H |
| COL4A1 | NM_001845 | Saito et al. [122] | mouse | NPE+PE | M-M |
| COL4A2 | NM_001846 | Saito et al. [122] | mouse | NPE+PE | M-M |
| COL4A3 | NM_000091 | Saito et al. [122] | mouse | PE | M-M |
| COL4A4 | NM_000092 | Saito et al. [122] | mouse | PE | M-M |
| COL4A5 | NM_033380 | Saito et al. [122] | mouse | NPE+PE | M-H* |
| COL4A6 | NM_033641 | Saito et al. [122] | mouse | NPE+PE | M-M* |
| CYGB | NM_134268 | Ostojic et al. [123] | human | NPE | L-L |
| CYP1B1 | NM_000104 | Bejjani et al. [124] | mouse | PE | H-H |
|  |  | Doshi et al. [125] | human | NPE |  |
| GJA1 | NM_000165 | Calera et al. [126] | mouse | NPE+PE | H-M |
|  |  | Coca-Prados et al. [127] | human | NPE+PE |  |
|  |  | Coffey et al. [128] | rat | NPE+PE |  |
| GJA5 | NM_005266 | Coffey et al. [128] | rat | NPE+PE | L-L |
| GJB2 | NM_004004 | Coffey et al. [128] | rat | NPE | L-L |
| GJB3 | NM_024009 | Coffey et al. [128] | rat | NPE | L-L |
| GLUT1 | NM_006516 | Tserentsoodol et al. [129] | mouse | NPE+PE | H-H |
| LAMA1 | NM_005559 | Wang et al. [130] | human | NPE+PE | L-L |
| LAMB2 | NM_002292 | Wang et al. [130] | human | NPE+PE | H-H |
| LAMB1 | NM_002291 | Wang et al. [130] | human | PE | H-H* |
| MITF | NM_198159 | Lord-Grignon et al. [4] | mouse | none | H-H* |
| MMP1 | NM_002421 | Lan et al. [131] | human | NPE+PE | L-L |
| MMP2 | NM_004530 | Lan et al. [131] | human | NPE+PE | M-M |
| MMP3 | NM_002422 | Lan et al. [131] | human | NPE+PE | VL-L |
| MMP9 | NM_004994 | Lan et al. [131] | human | NPE+PE | L-L |
| NES | NM_006617 | Lord-Grignon et al. [4] | mouse | none | L-L |
| NGB | NM_021257 | Ostojic et al. [123] | human | NPE | L-L |
| NLN | NM_020726 | Bertazolli-Filho et al. [132] | bovine+rat | NPE+PE | M-M |
| NT | NM_006183 | Ortego and Coca- Prados [133] | human | NPE+PE | L-L |
| OCLN | NM_002538 | Sonsino et al. [116] | human | NPE | M-M |
|  |  | Tserentsoodol et al. [129] | mouse | NPE |  |
|  |  | Wu et al. [134] | rabbit | NPE |  |
| PAX6 | NM_000280 | Lord-Grignon et al. [4] | mouse | NPE+PE | H-H |
| PEDF | NM_000934 | Ortego et al. [135] | human+bovine | NPE+PE | L-L* |
| PTGDS | NM_000954 | Gerashchenko et al. [136] | rat+mouse | NPE>PE | H-H |
| RHO | NM_000539 | Bertazolli-Filho et al. [137] | bovine | NPE | H-H |
| RHOK | NM_002929 | Bertazolli-Filho et al. [137] | bovine | NPE+PE | L-L |
| RLBP1 | NM_000326 | Martin-Alonso et al. [138] | bovine | NPE<PE | M-M |
|  |  | Salvador-Silva et al. [139] | bovine | PE |  |
| RAX | NM_013435 | Lord-Grignon et al. [4] | mouse | NPE+PE | H-H |
| SAG | NM_000541 | Bertazolli-Filho et al. [137] | bovine | NPE | H-H |
| SCG2 | NM_003469 | Ortego et al. [140] | rat | NPE | M-M |
| SCNN1A | NM_001038 | Rauz et al. [141] | human | NPE+PE | M-M |
| SCNN1B | NM_000336 | Rauz et al. [141] | human | NPE+PE | L-L |
| SGK | NM_005627 | Rauz et al. [141] | human | NPE+PE | H-H |
| SIX3 | NM_005413 | Lord-Grignon et al. [4] | mouse | NPE+PE | M-M |
| SIX6 | NM_007374 | Lord-Grignon et al. [4] | mouse | none | H-H |
| SLC12A1 | NM_000338 | Hochgesand et al. [142] | human | PE>NPE | L-L* |
| SLC12A2 | NM_001046 | Dunn et al. [143] | bovine | NPE+PE | M-M |
| SLC4A4 | NM_003759 | Bok et al. [144] | rat | PE | H-H* |
| SLCO1C1 | NM_017435 | Gao et al. [145] | human | NPE+PE | L-L |
| SLCO1A2 | NM_005075 | Gao et al. [145] | human | NPE | L-L |
| SLCO3A1 | NM_013272 | Gao et al. [145] | human | NPE+PE | H-H |
| SLCO4A1 | NM_016354 | Gao et al. [145] | human | NPE+PE | M-M |
| SLCO2B1 | NM_007256 | Gao et al. [145] | human | NPE | M-M |
| TF | NM_001063 | Bertazolli et al. [146] | rabbit | NPE+PE | H-H |
| TGFB2 | NM_003238 | Peress and Perillo [147] | human+rabbit | NPE+PE | H-M* |
| TGFB3 | NM_003239 | Peress and Perillo [147] | human+rabbit | NPE+PE | H-M* |
| TIMP-1 | NM_003254 | Lan et al. [131] | human | NPE+PE | M-M |
| TIMP-2 | NM_003255 | Lan et al. [131] | human | NPE+PE | M-M |
| TIMP-4 | NM_003256 | Lan et al. [131] | human | NPE+PE | L-L |
| ZO-1 | NM_003257 | Sonsino et al. [116] | human | NPE+PE | M-M |
|  |  | Tserentsoodol et al. [129] | mouse | NPE+PE |  |
|  |  | Wu et al. [134] | rabbit | NPE+PE |  |

We subdivided our expression data of the NPE and PE according to the mean expression intensity and percentiles. Subsequently, we put the data in different sub-groups according to the mean intensity: mean intensity >90^th^ percentile: high expression (H); mean intensity between 50^th^-90^th^ percentile: medium expression (M); mean expression intensity <50^th^ percentile: low expression (L). In another analysis of our data, we looked for genes that were statistically significantly different expressed between NPE and PE (see methods). These were indicated with an asterisk in the last column.

Abbriviations : NPE : non-pigmented epithelium ; PE : pigmented epithelium. Abbreviations of gene names are according to standard abbreviations used in Genbank.
