# Supplementary material for: Gene Expression and Functional Annotation of the Human Ciliary Body Epithelia
Source: PLoS One. 2012 Sep 18;7(9):e44973. doi: 10.1371/journal.pone.0044973 (PMC3445623; doi:10.1371/journal.pone.0044973)
Supplement: Table S3 — (DOC) Overview of the proteins and their protocols for immunohistochemistry. [file pone.0044973.s045.doc]

**Table S3. Overview of the proteins and their protocols for immunohistochemistry**

| **Gene** | **Function** | **Functionality of CB** | **Tissue fixation** | **Extra blocking step (yes/no)** | **1^st^ antibody** | **Abcam number** | **2^nd^ antibody** |
| --- | --- | --- | --- | --- | --- | --- | --- |
| FGFR3 | Mitogenesis and differentiation | Progenitor | PFA | no | α-rabbit polyclonal; 1:100 | Ab10649 | goat-α-rabbit Cy3; 1:200 |
| NOTCH1 | Developmental processes | Progenitor | PFA | yes | α-rabbit polyclonal; 1:50* | Ab52301 | goat-α-rabbit Cy3; 1:200 |
| ROBO1 | Axonal guidance and neuronal precursor cell migration | Neural | PFA | yes | α-rabbit polyclonal;1:100* | Ab7279 | goat-α-rabbit Cy3; 1:200 |
| GRIN2C | Glutamate receptor | Neural | acetone | yes | α-rabbit polyclonal;1:100* | Ab110 | goat-α-rabbit Cy3; 1:200 |
| EDNRB | Endothelin B receptor | Endocrine | acetone | no | α-rabbit polyclonal; 1:50* | Ab95911 | goat-α-rabbit Cy3; 1:200 |
| MNAR | Coactivator of estrogen receptor | Endocrine | PFA | no | α-rabbit polyclonal; 1:100 | Ab84094 | goat-α-rabbit Cy3; 1:200 |
| TLR3 | Pathogen recognition and activation of innate immunity | Immunological | acetone | no | α-mouse monoclonal; 1:50* | Ab13915 | goat-α-mouse Cy3; 1:200 |
| SERPING1 | Regulation of complement cascade | Immunological | PFA | yes | α-mouse monoclonal; 1:50* | Ab54898 | goat-α-mouse Cy3; 1:200 |

* Overnight incubation at 4°C
