# Supplementary material for: Gene Expression and Functional Annotation of the Human Ciliary Body Epithelia
Source: PLoS One. 2012 Sep 18;7(9):e44973. doi: 10.1371/journal.pone.0044973 (PMC3445623; doi:10.1371/journal.pone.0044973)
Supplement: Table S5 — Biological functions assigned by Ingenuity to the NPE and PE. (DOCX) [file pone.0044973.s047.docx]

**Table S5: Biological functions assigned by Ingenuity to the NPE and PE**

| **Developmental properties** | **Basic cellular (dis)functions** |
| --- | --- |
| Embryonic development | Gene expression |
| Cellular development | RNA post transcriptional modification |
| Tissue development | DNA replication, recombination and repair |
| Organ development | Protein synthesis |
| Organismal development | Protein folding |
| Hereditary disorder | Post-translational modification |
| Developmental disorder | Protein trafficking |
| Nervous system development and function | Protein degradation |
| Connective tissue development and function | Small molecule biochemistry |
| Skeletal and muscular system development and function | Molecular transport |
| Hepatic system development and function | Cell morphology |
| Visual system development and function | Cell cycle |
| Digestive system development and function | Cellular function and maintenance |
|  | Cellular assembly and organization |
| **Neurological function and disease** | Cellular growth and proliferation |
| Nervous system development and function | Cell-to-cell signaling and interaction |
| Neurological disease | Cellular movement |
| - Huntington’s disease | Cell death |
| - Leigh syndrome | Cellular compromise |
| - Dementia | Tissue morphology |
| - Alzheimer’s disease | Organ morphology |
| - Tauopathy | Organismal survival |
| - Neurodegenerative disorder | Cancer |
| - Gliosis | - Brain tumors |
| Psychological disorders | - Bone cancers |
|  | - Renal and urological cancers |
| **Endocrine and metabolic function** | - Gastrointestinal cancers |
| Endocrine system disorders | - Cancer of reproductive system |
| - Experimentally induced diabetes | - Thyroid cancer |
| Metabolic disease | - Lung cancer |
| - Mitochondrial complex I deficiency |  |
| - Iron overload | **Immunological functionalities** |
| Energy production | Inflammatory response |
| Nucleic acid metabolism | Inflammatory disease |
|  | Infectious disease |
| **Other** | Immunological disease |
| Hematological disease | - Autoimmune disease |
| - Waldenstrom’s macroglobulinemia | - Atopic dermatitis |
| - Hemosiderosis | - (Rheumatoid) arthritis |
|  | - Lichen planus and psoriasis |
