# Supplementary material for: Gene Expression and Functional Annotation of the Human Ciliary Body Epithelia
Source: PLoS One. 2012 Sep 18;7(9):e44973. doi: 10.1371/journal.pone.0044973 (PMC3445623; doi:10.1371/journal.pone.0044973)
Supplement: Table S6 — Canonical pathways assigned by Ingenuity to the NPE and PE. (DOCX) [file pone.0044973.s048.docx]

**Table S6: Canonical pathways assigned by Ingenuity to the NPE and PE**

| **Neurological function and disease** |
| --- |
| Huntington’s Disease Signaling |
|  |
| **Endocrine signaling and Metabolic function** |
| Estrogen Receptor Signaling |
| Glucocorticoid Receptor Signaling |
| Androgen Signaling |
| Ephrin B signaling |
| Ephrin receptor signaling |
| Protein Kinase A Signaling |
| EIF2 Signaling |
| Regulation of eIF4 and p70S6K Signaling |
| Aryl Hydrocarbon Receptor Signaling |
| mTOR Signaling |
| ILK Signaling |
|  |
| Glycolysis/Gluconeogenesis |
| Mitochondrial Dysfunction |
| Ubiquitone Biosynthesis |
|  |
| **Immunological functionalities** |
| Antigen Presentation Pathway |
| IGF-1 Signaling |
| Protein Ubiquitination Pathway |
|  |
| **Oxidative stress mechanism** |
| Oxidative Phosphorylation |
| NRF2-mediated Oxidative Stress Response |
| Hypoxia Signaling in the Cardiovascular System |
|  |
| **Cellular (dys)functions** |
| Polyamine Regulation in Colon Cancer |
| Nucleotide Excision Repair Pathway |
