# Supplementary material for: Gene Expression and Functional Annotation of the Human Ciliary Body Epithelia
Source: PLoS One. 2012 Sep 18;7(9):e44973. doi: 10.1371/journal.pone.0044973 (PMC3445623; doi:10.1371/journal.pone.0044973)
Supplement: Table S11 — Biological functions different between NPE and PE assigned by Ingenuity. (DOCX) [file pone.0044973.s053.docx]

**Table S11: Biological functions different between NPE and PE assigned by Ingenuity**

| **Developmental properties** | **Basic cellular (dis)functions** |
| --- | --- |
| Embryonic development | DNA replication, recombination and repair |
| Cellular development | Post-translational modification |
| Tissue development | Protein synthesis |
| Organ development | Amino acid metabolism |
| Organismal development | Cell signaling |
| Developmental disorder | Cell-to-cell signaling and interaction |
| Hereditary disorder | Cell cycle |
| Visual system development and function | Cellular growth and proliferation |
| Nervous system development and function | Cellular assembly and organization |
| Auditory and vestibular system development and function | Cellular function and maintenance |
| Connective tissue development and function | Cellular compromise |
| Skeletal and muscular system development and function | Cell morphology |
| Hair and skin development and function | Cellular movement |
| Hematological system development and function | Cell death |
| Cardiovascular system development and function | Free radical scavening |
| Respiratory system development and function | Molecule transport |
| Hepatic system development and function | Small molecule biochemistry |
| Digestive system development and function | Tissue morphology |
| Reproductive system development and function | Organ morphology |
| Renal and urological system development and function | Organismal functions |
|  | Organismal survival |
| **Endocrine and metabolic function** | Tumor morphology |
| Endocrine system disorders | Cancer |
| - Diabetes mellitus | - Tumorigenesis |
| Metabolic disease | - Metastasis |
| Lipid metabolism | - Adenocarcinoma |
| Carbohydrate metabolism | - Hemangioma |
| Vitamin and mineral metabolism | - Brain cancer |
| Nucleic acid metabolism | - Lung cancer |
| Drug metabolism | - Cancer of reproductive system |
|  | - Colorectal cancer |
| **Neurological function and disease** | - Skin tumor |
| Neurological disease |  |
| - Neurodegenerative disorder | **Immunological functionalities** |
| - Neuromuscular disease | Inflammatory disease |
| - Gliosis | - (Rheumatoid) arthritis |
| - Schizophrenia | - Inflammatory bowel disease |
| - Multiple sclerosis | - Primary biliary cirrhosis |
| - Tauopathy | - Encephalitis |
| Behavior | Immunological disease |
| Psychological disorder | - Autoimmune disease |
| Ophthalmic disease | Immune cell trafficking |
| - Microphthalmia | Inflammatory response |
| - Corneal dystrophy | Antigen presentation |
| Auditory disease | Infectious disease |
| - Deafness | - Entrance of virus |
|  | Lymphoid tissue structure and development |
| **Other** | Hematopoiesis |
| Cardiovascular disease | - Development lymphocytes |
| - Arteriosclerosis | - Development leucocytes |
| - Coronary artery disease |  |
| - Hypertension |  |
| - Hypertrophy |  |
| Hematological disease |  |
| - Cyanosis |  |
