# Supplementary material for: Gene Expression and Functional Annotation of the Human Ciliary Body Epithelia
Source: PLoS One. 2012 Sep 18;7(9):e44973. doi: 10.1371/journal.pone.0044973 (PMC3445623; doi:10.1371/journal.pone.0044973)
Supplement: Table S12 — Canonical pathways different between NPE and PE assigned by Ingenuity. (DOC) [file pone.0044973.s054.doc]

**Table S12. Canonical pathways different between NPE and PE assigned by Ingenuity**

| **Developmental properties** |
| --- |
| Human Embryonic Stem Cell Pluripotency |
| Wnt/β-catenin Signaling |
| Factors Promoting Cardiogenesis in Vertebrates |
|  |
| **Neurological function and disease** |
| Axonal Guidance Signaling |
| Neuropathic Pain Signaling In Dorsal Horn Neurons |
| Glioblastoma Multiforme Signaling |
|  |
| **Endocrine Signaling and Metabolic Function** |
| Endothelin-1 Signaling |
| Dopamine-DARPP32 Feedback in cAMP Signaling |
| LPS/IL-1 Mediated Inhibition of RXR Function |
| Protein Kinase A Signaling |
| Cellular Effects of Sildenavil (Viagra) |
| Phospholipid Degradation |
| Thrombin Signaling |
| Spingosine-1-phosphate Signaling |
|  |
| **Immunological properties** |
| Hepatic Fibrosis/Hepatic Stellate Cell Activation |
| Antigen Presentation Pathway |
|  |
| **Other functions** |
| Caveolar-Mediated Endocytosis Signaling |
| Role of NFAT in Cardiac Hypertrophy |
| Gap Junction Signaling |
|  |
| **Basic cellular (dys)functions** |
| Molecular Mechanisms of Cancer |
| Colorectal Cancer Metastasis Signaling |
| Ovarian Cancer signaling |
| Cell Cycle: G1/S Checkpoint regulation |
|  |
